# Prevention and control of malaria and sleeping sickness in Africa: Where are we and where are we going?

**DOI:** 10.1186/1756-3305-4-37

**Published:** 2011-03-16

**Authors:** Vincent Corbel, Marie-Claire Henry

**Affiliations:** 1 Centre de Recherche Entomologique de Cotonou (CREC), 06 BP 2604, Cotonou, Bénin; 2 Institut de recherche pour le développement (IRD), Maladies Infectieuses et Vecteurs, Ecologie, Génétique, Evolution et Contrôle (MIVEGEC), UM1-CNRS 5290-IRD 224, 01 BP 4414 RP Cotonou, Bénin; 3 Service de Coopération et d'Action Culturelle, Ambassade de France, Cotonou, Bénin

## Abstract

The International Symposium on *Malaria and Human African Trypanosomiasis: New Strategies for their Prevention & Control *was held 7-8 October, 2010 in Cotonou, Benin with about 250 participants from 20 countries. This scientific event aimed at identifying the gaps and research priorities in the prevention and control of malaria and sleeping sickness in Africa and to promote exchange between North and South in the fields of medical entomology, epidemiology, immunology and parasitology. A broad range of influential partners from academia (scientists), stakeholders, public health workers and industry attempted the meeting and about 40 oral communications and 20 posters were presented by phD students and internationally-recognized scientists from the North and the South. Finally, a special award ceremony was held to recognize efforts in pioneer work conducted by staff involved in the diagnostic of the Sleeping illness in West Africa with partnership and assistance from WHO and Sanofi-Aventis group.

## Background

For about ten years now the United Nations Millennium Declaration defined the Millennium Development Goals of which some are to promote the fight against poverty, gender equality, accessibility to education and improvement in health. For health, particularly in infectious diseases, one of the clearly enunciated goals is to halt and begin to reverse the incidence of malaria by 2015, and bring hitherto neglected tropical diseases under control in the same time-frame[1]. However, just four years before the deadline, both malaria and human African trypanosomiasis (HAT) are still overwhelming developing countries. The spread of resistance--both to drugs by the parasites and to insecticides by the vectors--is a major concern which constantly raises questions about the effectiveness of "current" control strategies.

In this context, it is the right time to assess the limitations and gaps in knowledge of the prevention and control of these diseases particularly in Africa. For example, what are the main causes of emergence and/or spread? Do we have effective screening methods/strategies for detection of parasites, especially for HAT? Do we have significant progress in malaria vaccination, notably in vaccines targeting the most vulnerable groups? Do we have new/alternative prophylactic drugs for pregnant women to counteract the growing resistance of malaria parasites to sulfadoxine-pyrimethamine (SP)? Are there any new 'weapons' to ensure effective control of malaria transmission and to manage the development of pyrethroid-resistance in malaria vectors? These issues are particularly relevant to address considering the mobilization of funds by international organisations such as the Bill & Melinda Gates Foundation and World Health Organization (WHO) that are planning ambitious programmes of elimination and even eradication for malaria and sleeping sickness.

## Report

The International Symposium on *Malaria and Human African Trypanosomiasis: New Strategies for their Prevention & Control *that was held in Cotonou, Benin on the 7^th ^and 8^th ^of October 2010 http://www.symposiumpalutha.org aimed at identifying the gaps and research priorities in the prevention and control of malaria and sleeping sickness in Africa and to promote exchange between North and South in the fields of entomology, epidemiology, immunology and parasitology (see additional files 1 &2).

The discussion in the first session of the symposium showed that significant advances in the prevention and control of sleeping sickness, made in West Africa, are opening the way to the possibility of eradication in the medium term [2,3]. Measures were proposed to help realize this ambition. It has been proposed to conduct geo-epidemiological surveys to identify the most important regions for action and rationalise expenditure on control. In order to cover more people, the diagnosis of HAT will have to be improved. For example, better understanding of what a positive CATT (Card Agglutination Test for Trypanosomiasis) result means, in the absence of parasitological confirmation, will be needed to guide the control strategy to adapt on a local basis [4]. In a context of eradication, in which prevalence may be very low, efficient surveillance systems will have to be established based on more sensitive tests for host exposure to the parasite (e.g. the trypanolysis test) or the vector (e.g. assaying antibodies against tsetse saliva). Treatment will be effectively dispensed and made accessible to all the sick, even those in the most remote areas. It will also be important to consolidate communities' capabilities in terms of advocacy, consciousness-raising, education and information.

Improving international coordination in HAT control and prevention will be essential, notably through the establishment of an integrated, multidisciplinary approach at the regional level but also by bringing in new collaborators, from both public and private sectors, from both hemispheres e.g. WHO, Pan African Tsetse and Trypanosomiasis Eradication Campaign (PATTEC), National Sleeping Sickness Control Programme, Institut de Recherche pour le Développement (IRD), etc. In West Africa, efforts will have to be geographically focused on Guinea, the West African country that is most severely affected by HAT. Moreover, efforts will have to cover Ivory Coast where the disease is still endemic despite massive control efforts in recent years as well as other countries i.e. Guinea-Bissau, Liberia, Sierra Leone and Senegal for which no epidemiological data are available. In countries where HAT is no longer a public health problem, epidemiological surveillance will have to be maintained because the risk of re-emergence still exists due to the presence of the vector and to increasing immigration within the region [5].

In the second session, the participants fruitfully discussed the consequences of malaria in pregnant women and foetal outcomes. An assessment of intermittent preventive treatment (IPT) for malaria in pregnant women conducted in various settings in Benin showed a sharp increase in IPT coverage as of the launch of follow-up in 2006, but coverage was still far below the objective of 80% recommended by WHO and the National Malaria Control Programme (NMCP) (D'almeida T, et al, Field evaluation of the intermittent preventive treatment of malaria during pregnancy (IPTp) in Benin: evolution of the coverage rate since its implementation, submitted) [6]. The results showed disparities, with better coverage in the north and in rural areas than in the south and in urban settings. Difficulties were also noted with drug handling and supply, especially in maternity units in southern towns. Taken together, these results point to the need for effective assessment at the national level, ideally through collaborations between research institutions and the NMCP. It has been recommended to improve the dispensing and supervision of IPT (two effective doses) by ensuring the drug supply and by consolidating the training of dispensary and hospital staff. Other findings showed that, for IPT, mefloquine (MQ) was as effective as SP at preventing low birth weight [7]. Moreover MQ was more effective than SP at preventing both placental malaria infection and maternal anaemia at delivery. Mefloquine can be seen as a promising alternative drug for the prevention of malaria in pregnant women although further data are required on safety and compliance. Given the development of drug resistance (e.g. Chloroquine, SP, artemeter and artesunate) in malaria parasites across the world, it has been emphasized that researchers and NMCPs will have to establish active surveillance systems to monitor resistance to antimalarials and investigate the underlying mechanisms of action. Research of alternative preventive methods, especially vaccines is also urgently required in the perspective of malaria elimination.

The results of a regional REFS-FSP project http://www.ambafrance-bj.org/france_benin confirm that the new-born babies of mothers infected with *Plasmodium falciparum *at delivery are particularly susceptible to malaria [8]. Important findings were reported on the role of genetic, immunological and environmental factors in malaria infection that will help in the development of a new candidate vaccine for young children in a few years. Moreover, an IRD team in Benin showed that a candidate vaccine during pregnancy, VAR2CSA, was able to induce a protective immunological response. More laboratory and field studies are needed to evaluate the performances and acceptability of this vaccine in pregnant women.

Session 3, dealing with innovative strategies for malaria vector control, emphasized the inexorable rise in the resistance of malaria vectors to insecticides and the consequences for malaria control in Africa based on treated mosquito nets (ITNs) and indoor residual spraying (IRS). Given the widespread resistance to pyrethroids in malaria vectors [9], these molecules seem unsuitable for indoor spraying operations. Recommendations were made to keep pyrethroids for mosquito net treatment only in order to minimize the risk of selection on existing resistance genes. Some scientists suggested including new vector control (VC) tools in the prevention of malaria, e.g. long-acting insecticide materials, tarpaulins or wall-coverings such as Durable Wall Lining (DL). The latter showed particularly promising results in Angola as substitutes to IRS because they required lowest investments in terms of human and financial resources. However, this type of material is currently available with pyrethroids only, so that there is a need to use other classes of insecticide for treatments, e.g. carbamates, pyrroles, neonicotinoids and/or growth regulators. Furthermore, there have been a number studies exploring the potential for using entomopathogenic fungi as novel biopesticide interventions against adult malaria mosquito vectors. Recent findings showed that fungal pathogens do not suffer cross resistance with chemical insecticides and can even exhibit insecticide resistance-breaking properties, highlight the potential for use of insect fungal pathogens within novel strategies of integrated vector management [10]. Beyond these innovative tools, it was also recommended to conduct more field evaluations on the performances of new VC strategies on malaria disease. In Benin, a randomised, controlled trial conducted in Southern Benin showed no benefit when combining carbamate IRS with LLIN on the reduction of transmission, prevalence of infection and malaria attacks in children aged less than six years [11,12]. Increased pyrethroid resistance, inconsistent ITN use and significant outdoor transmission were suspected to be responsible for the absence of mass community effects with combined residual interventions. Researches will thus have to focus on behavioural changes in malaria vectors after VC operations and to identify complementary methods to better target exophagous and exophilic mosquito populations (e.g. new attractant traps, push-pull strategies, treating of resting areas, larvicidal operations, etc.).

An important point raised by the scientists was to include also social sciences into all entomo-epidemiological evaluations of new VC tools. In the same way, bringing local people in when compiling "Study Protocols" and preparing VC operations is essential to guarantee local support and commitment for the project. It is obvious that consciousness-raising campaigns (radio, television, posters, etc.) are key points when it comes to improving the acceptance of VC tools such as LLIN which remains the main way of community-based prevention in Africa. Improvements may be useful as well on the criteria and indicators used to evaluate VC, especially to better reflect human-vector contact after implementation of an intervention. A newly developed immunological biomarker that measures specific exposure of subjects to the vector proved to be highly sensitive in the evaluation of a massive ITN distribution campaign in Angola at both the community and individual levels [13]. This new "salivary" biomarker could be ideal for evaluating control programmes and would cut down the need for human capture methods which pose both ethical and logistic problems.

Considering the amounts invested in malaria and HAT prevention and control in the last decade (by the World Bank, the Global Fund, the President's Malaria Initiative, the Bill & Melinda Gates Foundation, etc.), a rigorous, multidisciplinary evaluation of the public health programmes is indispensable. The evaluation will have to include both VC interventions and antimalarial drugs (CTA, SP, etc.), the monitoring of vector resistance to insecticides and parasite resistance to drugs, the estimates of compliance, safety and the acceptance of treatments by populations. Building the capacities in medical entomology, parasitology, epidemiology, immunology, at research institutions, universities and national control programmes will also be a priority. This could be achieved through collaborative research programmes, the founding of federative research laboratories (e.g. the *Centre de Lutte Intégrée contre le Paludisme *in Benin), effective training programmes and the establishment of International Master's Degrees (e.g. the International Master's Degree in Entomology http://www.miemv.org. IRD is, for example, supporting the creation of International Mixed Laboratories (LMI) and young associate teams (JEAI) to help scientists from the South to develop their research programmes http://www.ird.fr.

Before the world is rid of malaria and neglected diseases, research institutes and national control programmes will have a key role to play in the prevention and control of these diseases. It is therefore important to strengthen research and training capacities in the South, and foster synergistic interactions between the different actors to promote strategic decision-making in public health.

## Competing interests

The authors declare that they have no competing interests.

## Authors' contributions

VC and MCH led the organization and scientific committees of *the International Symposium on Malaria and Human African Trypanosomiasis: New Strategies for their Prevention & Control *and wrote the meeting report. All authors read and approved the final manuscript.

## Supplementary Material

Additional file 1 **Proceedings of the international symposium**. Click here for file

Additional file 2 **Scientific Committee**. Click here for file
