# Supplementary material for: Prevention and control of malaria and sleeping sickness in Africa: Where are we and where are we going?
Source: Parasit Vectors. 2011 Mar 16;4:37. doi: 10.1186/1756-3305-4-37 (PMC3065431; doi:10.1186/1756-3305-4-37)
Supplement: Additional file 1 — Proceedings of the international symposium. [file 1756-3305-4-37-S1.PDF]

Symposium international / International Symposium

PALUDISME ET TRYPANOSOMOSE HUMAINE AFRICAINE : NOUVELLES STRATEGIES DE PREVENTION ET DE CONTROLE

MALARIA AND HUMAN AFRICAN TRYPANOSOMIASIS: INNOVATIVE STRATEGIES FOR THEIR PREVENTION AND CONTROL

07 - 08 Octobre 2010, Palais des Congrès, Cotonou, Bénin

Cher(e) participant(e),

Le comité d'organisation vous souhaite la bienvenue à Cotonou et vous remercie pour votre présence au symposium international « ***Paludisme et Trypanosomose Humaine Africaine: nouvelles stratégies de prévention et de contrôle*** » qui se tiendra au Palais des Congrès les 07 et 08 octobre 2010. Ce symposium international a pour objectif d'identifier les nouveaux axes prioritaires de recherche et de formation dans la prévention et la lutte contre le paludisme et la maladie du sommeil (THA) et de favoriser les échanges scientifiques Nord / Sud dans les domaines de l'entomologie, de l'épidémiologie, de l'immunologie et de la parasitologie.

A ce jour, dix ans se sont écoulés après la signature au siège des Nations Unies du protocole d'accord définissant les Objectifs du Millénaire en matière de développement, de la lutte contre la pauvreté, d'égalité des sexes, d'accès à l'éducation et d'amélioration de la santé. Sur ce dernier plan, et plus particulièrement dans le domaine des maladies infectieuses, les objectifs clairement affichés par les grandes puissances visaient notamment à ramener à un taux proche de zéro le taux de mortalité lié au paludisme et de maîtriser les maladies tropicales négligées d'ici à 2015. Pourtant, à 5 ans du terme de ces objectifs, force est de constater que les maladies infectieuses dont le paludisme et la Trypanosome Humaine Africaine (THA), demeurent toujours un fléau dans les pays en développement. Le développement des résistances des parasites aux médicaments et des vecteurs aux insecticides est préoccupant et remet sans cesse en cause les stratégies « conventionnelles » de lutte contre ces maladies.

Dans ce contexte, il nous semble important de dresser un bilan sur les avancées obtenues dans le domaine de la prévention et du contrôle de ces maladies. A titre d'exemple, quels sont les facteurs responsables de l'émergence et/ou de l'extension de ces maladies en Afrique ? Quelles sont les nouvelles stratégies de dépistage de la maladie du sommeil ? Quelles sont les avancées obtenues sur le(s) vaccin(s) contre le paludisme, en particulier ceux ciblant les groupes les plus vulnérables ?

Quelles sont les molécules alternatives disponibles pour le traitement préventif de la femme enceinte ? Dispose-t-on de nouvelles « armes» pour lutter contre les vecteurs devenus résistants aux insecticides ?

Nous espérons que ce symposium apportera des éléments de réponses à ces questions. Ceci est particulièrement pertinent dans le cadre du lancement, par les grands bailleurs de fonds (Fondation Bill & Mellinda Gates notamment) et les organisations internationales (OMS, etc.), de programmes ambitieux d'élimination voire d'éradication du paludisme et de la THA. Avant de voir un monde « débarrassé » de ces maladies, il est certain que les instituts de recherche et les Programmes Nationaux de Lutte (PNL) auront un rôle fondamental à jouer dans l'amélioration de la prévention et la lutte contre ces maladies sur le continent. Il est donc impératif de renforcer les capacités de recherche et de formation au SUD et d'amplifier les synergies (réseaux) entre ces différents acteurs afin d'aider à la décision stratégique en matière de politique de santé.

Avant de poursuivre plus longuement le débat lors du symposium, nous espérons que vous passerez un très agréable séjour à Cotonou et que vous apprécierez les sessions et les conférences scientifiques qui y seront présentées.

Bien cordialement

Le Comité d'organisation

Dear Delegate(s),

The organising committee welcomes you to Cotonou and thanks you for attending this International Symposium on ***Malaria and Human African Trypanosomiasis: New Strategies for their Prevention & Control***, to be held at the *Palais des Congrès* on the 7th and 8th October 2010. The goal of this symposium is to identify novel research priorities in the prevention and control of malaria and sleeping sickness, and to promote exchange between North and South in the fields of entomology, epidemiology, immunology and parasitology.

It has been ten years since the signing of the United Nations Millennium Declaration defining the Millennium Development Goals to promote the fight against poverty, and promote sexual equality, access to education and improvement in health. For health—particularly in infectious diseases—the clearly enunciated goals were to bring malaria mortality down to close to zero by 2015, and bring

hitherto neglected tropical diseases under control in the same time frame. However, just five years from the deadline, both malaria and sleeping sickness are still devastating developing countries. The spread of resistance—both to drugs by the parasites and to insecticides by the vectors—is a major concern and one that is constantly raising questions about "conventional" control strategies.

In this context, it is an opportune moment to review the progress made in preventing and controlling these diseases. By way of example, "What factors underlie the emergence and spread of these diseases in Africa?" "What new screening methods/strategies are available for detection of sleeping sickness?" "What progress has been made in malaria vaccination, notably in vaccines targeting the most vulnerable groups?" "What prophylactic drugs are available for pregnant women?" "Are there any new 'weapons' for the control of insecticide-resistant vectors?"

We hope that this symposium will go some way to answering these questions. This is particularly relevant with the mobilisation of funds by charitable organisations—notably the —Bill & Melinda Gates Foundation—and international organisations like the WHO, for ambitious programmes aiming to eliminate and even eradicate malaria and sleeping sickness. Before the world is rid of these diseases, it is sure that research institutes and National Control Programmes will have a key role to play in prevention and control of these diseases in Africa. Therefore, it is important to strengthen research and training capacities in the South, and foster synergistic interactions between the various protagonists to promote strategic decision-making in public health.

Before we start on the in-depth debates of the symposium, we wish you a pleasant stay in Cotonou, and hope that you will profit from these scientific presentations and exchanges.

Yours sincerely,

The Organising Committee

OBJECTIFS

Ce Symposium international a pour objectif d'identifier les nouveaux axes prioritaires de recherche et de formation dans la prévention et la lutte contre le paludisme et la maladie du sommeil (THA) et de favoriser les échanges scientifiques Nord / Sud dans les domaines de l'entomologie, de l'épidémiologie, de l'immunologie et de la parasitologie. Ce Symposium permettra également de présenter les avancées scientifiques réalisées dans le cadre du projet régional REFS (http://www.ambafrance-bj.org/france_benin/) qui a impliqué les chercheurs de 6 instituts et universités d'Afrique de l'ouest (Bénin, Burkina Faso, Guinée) et de France ainsi que les Programmes Nationaux de Lutte pendant 3 ans (2007-2010).

Le Symposium couvrira les thématiques suivantes :

- Nouvelles stratégies de prévention et de contrôle des Trypanosomoses Africaines.

- **Conséquences du Paludisme chez la femme enceinte et le nouveau né dans la perspective de développement d'un vaccin pour la femme enceinte**

- Approches innovantes de lutte et de gestion des résistances chez les vecteurs du paludisme

Le contenu des communications orales et des posters présentés durant le symposium mettra l'accent sur les thèmes suivants ;

- **Les outils génétiques, géographiques et épidémiologiques utilisés dans la lutte contre les glossines (Tsé Tsé) et le dépistage de la maladie du sommeil chez l'homme**

- **Les marqueurs épidémiologiques, immunologiques et génétiques (humaine et parasitaire) de la physiopathogie du paludisme associée à la grossesse et du jeune enfant**

- **La recherche d'alternative au Traitement Préventif Intermittent basé sur la sulfadoxine-pyriméthamine (SP).**

- **Les nouvelles stratégies de contrôle des vecteurs et de gestion de la résistance aux insecticides et leur évaluation entomologique, épidémiologique et immunologique.**

Il s'adresse à :

- la communauté scientifique (chercheurs, étudiants, milieu universitaire, etc.) concernée par les maladies à vecteurs
- les opérateurs de santé publique (managers, médecins, et directeurs des Programmes Nationaux de Lutte contre le Paludisme et la THA)
- les décideurs politiques
- Les industriels

OBJECTIVES

This is a symposium to serve as a forum for discussing the perspectives and priorities in terms of prevention and control of malaria and HAT in Africa. It will also promote exchanges of ideas among Entomologists, Epidemiologists, Parasitologists and Immunologists working on these major Diseases in Africa. We will also promote scientific advances achieved in parasite detection, prevention and control of malaria and sleeping sickness in West Africa through the regional REFS project (http://www.ambafrance-bj.org/france_benin/). This project conducted in close collaboration with the National Malaria and sleeping Sickness Control Programmes between 2007 and 2010 involved scientists from 6 institutions and universities from France and West Africa (Benin, Burkina Faso, Guinea, and Cote d'Ivoire)
The symposium will focus on the following topics:

- **New Strategies for the prevention and control of Trypanosomiasis in Africa.**
- **Consequences of malaria during pregnancy and associated foetal outcomes with the prospect of development of specific vaccine for pregnant women**
- **Innovative strategies for controlling and managing insecticide-resistant malaria vectors**

The content of posters and oral communications presented during the symposium should focus on:

- **Genetic, geographic and epidemiological tools for Tse-Tse fly control and diagnostic methods for sleeping sickness**
- **Epidemiological, immunological and genetic factors (human and parasite) involved in the physiopathology of malaria in pregnant women and young children**

- **Alternative medicines to sulfadoxine-pyrimethamine (SP) for Intermittent Preventive treatments (IPT)**
- **Entomological, epidemiological and immunological evaluation of innovative malaria vector control strategies**

This symposium is of particular interest for:

- The scientific community (scientists, students, academia) involved in control of vector borne diseases
- Public health sectors (managers, physician, directors of National Malaria and Sleeping Sickness Control programmes, etc)
- Stakeholders
- Industry

INFORMATION GENERALE

◆ **Inscriptions**

Les inscriptions se dérouleront le Jeudi matin entre 08H00 et 09H00 devant la salle de conférence (« salle Bleue »).

◆ **Accès au Palais des Congrès**

Le Palais des Congrès de Cotonou se situe sur la route de l'Aéroport International de Cotonou (Boulevard de la Marina, B.P. 147 Gbèdégbé Cotonou – Bénin. Tél. : (229) 21.30.91.10 / 21.30.91.29). Un bus (limité à 15 places) assurera le transport des participants de leurs hôtels (Ibis, Cocotiers, Hôtel du Port) vers le Palais des Congrès (et vice versa).

Matin : entre 07H45 et 09H00

Midi: ente 12H00 et 14H30

Soir: entre 18H00 et 19H30.

Le bus assurera également le transport des participants lors du cocktail dînatoire prévu le vendredi 08 au soir.

◆ **Présentation orale**

Toutes les sessions et conférences scientifiques se dérouleront dans la « salle bleue » du Palais des Congrès (à gauche en arrivant sur l'esplanade). Les orateurs sont invités à fournir à un membre du Comité d'organisation (COS) leurs présentations en version PPT au moins 30 minutes avant le début de chaque session. Les communications dureront 15 minutes maximum (10 minutes de présentation et 5 minutes de question) tandis que les conférences dureront 30 minutes (20 minutes de présentation et 10 minutes de question).

◆ **Poster**

Trois sessions de Posters sont prévues pendant les pauses café. La première se déroulera le Jeudi 07 Octobre entre 16H15 et 16H45 (session 1). Les deuxième et troisième sessions se dérouleront le Vendredi 08 Octobre entre 10H45 et 11H15 (session 2) et entre 16H15 et 16H45 (session 3). Des panneaux seront prévus pour afficher les posters. Merci de rester près de vos posters pour répondre aux éventuelles questions. Ils seront exposés dans le hall extérieur du Palais des Congrès durant les deux jours de conférence. Vous êtes priés de prendre contact avec un membre du COS en cas de problème d'affichage.

◆ **Pause Déjeuner**

La pause déjeuner se déroulera entre 13H00 et 14H00 dans le « Hall des cocktails » au premier étage du Palais des Congrès.

◆ **Pause café**

Les pauses café auront lieux deux fois par jour dans le hall extérieur du Palais des Congrès.
Le jeudi 07 octobre entre 11H00 et 11H30 et entre 16H15 et 16H45
Le vendredi 08 octobre entre 10H45 et 11H15 et entre 16H15 et 16H45.

◆ **Réception / Cocktail**

Un cocktail dînatoire aura lieu le Vendredi 08 au soir au restaurant le « Berlin » (Terrasse le Titanic, dernier étage) situé au 06 BP 2535 Cotonou (quartier Ganhi), à partir de 19H30 (tel 21 31 74 78, restaurantberlin@yahoo.fr). Veuillez vous prémunir de votre badge de conférence qui fera office de ticket d'entrer à la réception.
L'artiste béninois « Petit Miguelito », parrain du symposium, et son groupe « Les étoiles du Bénin » assureront l'animation musicale pendant la soirée.

Une réception, sur invitation, aura lieu le jeudi soir à la représentation de l'IRD à partir de 19H30.

◆ **Contacts utiles**

En cas de problèmes, vous pouvez joindre les personnes suivantes ;

Comité d'organisation du Symposium (COS)

- Lin Atigli : 95 12 16 82, constantlin@yahoo.fr
- Marie Claire Henry : 97 39 58 17, Marie-Claire.Henry@ird.fr
- Vincent Corbel : 95 91 50 92, vincent.corbel@ird.fr

Service Presse IRD

- Rita saudegbee, 21.30.03.54, rita.saudegbee@ird.fr
- Christelle Duos, 96 00 06 56, cristelle.duos@ird.fr

Institut de Recherche pour le Développement

- Secrétariat ; 21 30 03 54, Bellinda.Hounmasse@ird.fr
- Représentant : 21 30 03 52, Bruno.Bordage@ird.fr

Ambassade de France à Cotonou

- Secrétariat : 21.30.02.25 ou 21.30.02.26, ambafrance.cotonou@diplomatie.gouv.fr

Consulat de France à Cotonou

- Secrétariat; 21 31 26 80, consulat.cotonou-fslt@diplomatie.gouv.fr

Police

- Brigade Anti Crimanilté: 4545

General Information

◆ Registration

Registration will take place between 8 and 9 a.m. in front of the Conference Hall (the "Blue Room").

◆ Access to the *Palais des congrès*

The *Palais des Congrès de Cotonou* is on the road from the International Airport: Boulevard de la Marina, B.P. 147 Gbèdégbé Cotonou - Bénin - Tel.: (229) 21.30.91.10 / 21.30.91.29). A shuttle-bus (limited to 15 places) will be provided to pick up delegates from their hotels (Ibis, Cocotiers, Hôtel du Port) and bring them to the *Palais des Congrès* (and take them back).

Morning: between 7.45 and 9.00 a.m.

Midday: between 12.00 a.m. and 2.30 p.m.

Evening: between 6.00 and 7.30 p.m.

The bus will also bring delegates for the cocktail party on the evening of Friday 8th October.

◆ Talks

All the scientific sessions and talks will be presented in the "Blue Room" of the *Palais des Congrès* (to the left when you arrive via the esplanade). Speakers are asked to give the support material (in Powerpoint format) for their presentation to a member of the Organising Committee at least thirty minutes before the beginning of the session. Brief presentations should last no more than fifteen minutes (a ten-minute talk followed by five minutes of questions and answers) and lectures for thirty minutes (a twenty-minute talk followed by ten minutes of questions and answers)

◆ **Posters**

Three Poster Sessions are planned for the coffee breaks. The first will be on Thursday 7 October between 4.15 and 4.45 p.m. (Session 1). The second and third sessions will be on Friday 8 October, between 10.45 and 11.15 a.m. (Session 2), and between 4.15 and 4.45 p.m. (Session 3). Panels will be provided to display the posters. Please stay by your poster to answer any questions. Posters will be displayed in the lobby of the conference centre over both days of the meeting. Please contact a member of the Organising Committee if you have any problems putting your poster up.

◆ **Lunch breaks**

A break for lunch will be made between 1.00 and 2.00 p.m. in the "Cocktail Hall" on the first floor of the *Palais des congrès.*

◆ **Coffee breaks**

There will be two coffee breaks each day in the lobby outside the *Palais des congrès*:

On Thursday 7 October between 11.00 and 11.30 a.m., and between 4.15 and 4.45 p.m.

On Friday 8 October between 10.45 and 11.15 a.m., and between 4.15 and 4.45 p.m.

◆ **Reception / Cocktail party**

There will be a cocktail party on the Friday evening in the Berlin restaurant (the Titanic Terrace on the top floor) at 06 BP 2535 Cotonou (in the Ganhi neighbourhood), starting at 7.30 p.m. (Tel: 21 31 74 78, restaurantberlin@yahoo.fr). Please bring your conference badge to get into the reception.

The Beninese musician "*Petit Miguelito*", the symposium's official patron, will play during the evening with his group "*Les Étoiles du Bénin*".

On the Thursday evening, there will be a reception on invitation to a presentation of the *Institut de Recherche pour le Développement* (IRD: Development Research Institute), starting at 7.30 p.m.

◆ **Proceedings**

The Editor-in-Chief of *Parasites & Vectors* is supporting the symposium by devoting three articles in the journal to results presented at the scientific sessions. The Organising Committee has decided to reserve these articles for young scientists and physicians (from both North and South). The best scientific communication from each session will be selected and submitted for publication.

◆ Useful contacts

If you have any questions, you may join any of the following:

Organising Committee

- Lin Atigli: 95 12 16 82, constantlin@yahoo.fr
- Marie Claire Henry: 97 39 58 17, Marie-Claire.Henry@ird.fr
- Vincent Corbel: 95 91 50 92, vincent.corbel@ird.fr

IRD Press Department

- Rita Saudegbee: 21 30 03 54, rita.saudegbee@ird.fr
- Christelle Duos: 96 00 06 56, cristelle.duos@ird.fr

Institut de Recherche pour le Développement

- Secretariat: 21 30 03 54, Bellinda.Hounmasse@ird.fr
- Representative: 21 30 03 52, Bruno.Bordage@ird.fr

French Embassy at Cotonou

- Secretariat: 21 30 02 25 or 21 30 02 26, ambafrance.cotonou@diplomatie.gouv.fr

French Consulate at Cotonou

- Secretariat: 21 31 26 80, consulat.cotonou-fslt@diplomatie.gouv.fr

Police

- Criminal Brigade: 4545

MESSAGE ADRESSE PAR LE PARAIN DU SYMPOSIUM : « PETIT MIGUELITO », MUSICIEN ET COMPOSITEUR BENINOIS

Le paludisme est un problème grave en Afrique en particulier dans mon Pays, le Bénin. Nous sommes heureusement « épargnés » par la maladie du sommeil mais je sais qu'elle peut causer de sérieux problèmes dans les pays voisins. Etant déjà ambassadeur UNICEF pour l'aide aux réfugiés, je suis particulièrement touché par les problèmes liés à la pauvreté et je souhaite à mon niveau, encourager toutes les actions qui pourraient aider mon pays à mieux se protéger contre le paludisme.

Je suis fier d'être le parrain officiel de ce symposium sur la lutte contre le paludisme et la THA. J'ai décidé d'y apporter ma modeste contribution en composant une chanson qui je l'espère nous aidera à mieux sensibiliser les populations à la lutte contre cette maladie.

La chanson s'intitule «**Tu le sais** ».

Affectueusement

A MESSAGE FROM THE SYMPOSIUM'S PATRON, THE BENINESE ARTIST "PETIT MIGUELITO".

Malaria is a terrible problem in Africa, and especially in my country, Benin. Happily, we are free of sleeping sickness but I know that it causes serious problems in neighbouring countries. As a UNICEF ambassador for help to refugees, I am particularly touched by the problems of poverty, and I am committed to doing everything I can to encourage any activity that could help my country rid itself of malaria.

I am proud to be the official patron of this Symposium on malaria and sleeping sickness. I have decided to make my modest contribution by composing a song which I hope will help raise people's consciousness about the fight against this devastating disease.

The song is called "**Tu le sais**".

Affectionately,

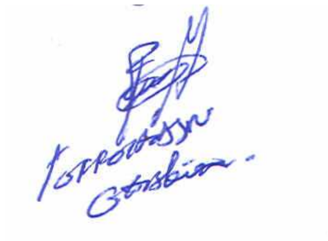

Petit Miguelito

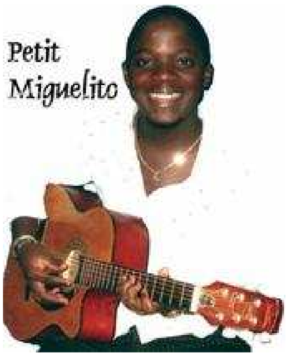

Palais des Congrès – Cotonou

07/10/2010

Time	Scientific programme	Chairmans
08:00-09:00	**Registration for symposium**	
09:00-10:15	**Official opening ceremony**	L. Atigli (COS)
10:15-11:00	**Prize-giving ceremony to veterans of sleeping sickness control**	P. Cattant
11:00-11:30	Coffee break	
11:30-12:30	**Past, present, and future of Human African Trypanosomiasis control in West Africa : Contribution of WHO and his partners**	P. Cattant
11:30-11:50	Evolution historique de la THA en Afrique occidentale depuis le début du 20ième siècle.<br>**F. Courtin**	
11:50-12:10	Situation actuelle de la THA.<br>**A. Diarra**	
12:10-12:30	Contribution des partenaires à la lutte contre la THA.<br>**B. Blayney**	
12:30-13:00	Recherche et Formation dans la lutte contre les maladies à transmission vectorielle : quelle place dans la prise de décision en santé publique ?<br>**M.-C. Henry**	

13:00-14:00 Lunch

14:00-18:30	**<u>Session 1</u>**<br><br>**NEW STRATEGIES FOR THE PREVENTION AND CONTROL OF TRYPANOSOMIASIS IN AFRICA**	
**14:00-15:00**	**Scientific Conferences**	P. Simarro & I. Sidibé
14:00-14:30	Le diagnostic de la Trypanosomose Humaine Africaine : statu quo ou temps du changement ?<br>**V. Lejon**	
14:30-15:00	La lutte anti-vectorielle dans les stratégies de prévention et de contrôle des Trypanosomoses Africaines<br>**P. Solano**	
**15:00-16:15**	**Scientific Session 1**	V. Jammoneau & M. Camara
15:00-15:15	A geographical approach to identify sleeping sickness risk factors in a mangrove ecosystem.<br>**F. Courtin**	
15:15-15:30	Population genetic structure of Trypanosoma brucei gambiense in coastal Guinea, control implication<br>**J. Kaboré**	
15:30-15:45	Use of immune trypanolysis to optimize epidemiological surveillance and control of sleeping sickness<br>**V. Jamonneau**	
15:45-16:00	*Sodalis glossinidius* and vector competence of field tsetse flies in two historical Human African Trypanosomiasis foci, Southern Cameroun<br>**O. Farikou**	
16:00-16:15	Caractérisation immunologique de la diversité clinique de la trypanosomose humaine africaine en Guinée.<br>**H. Ilboudo**	
16:15-16:45<br><br>**16:45-18:00**	Coffee break<br>*Poster session 1*<br>**Scientific Session 2**	B. Bucheton & R. Kambiré
16:45-17:00	Campagne d'éradication des glossines dans les îles de Loos (République de Guinée): résultats actuels<br>**M. Kagbadouno**	
17:00-17:15	A new transmission Risk Index of Human African Trypanosomiasis and its evaluation in the Fontem focus<br>**G.R. Njitchouang**	
17:15-17:30	Importance de la morphométrie géométrique dans le choix de la stratégie de lutte contre les tsétsés<br>**D. Kaba**	
17:30-17:45	Prospects of fungus-contaminated device for the control of tsetse fly<br>**N.K. Maniania**	
17:45-18:00	Test de compétitivité de *Glossina palpalis gambiensis* mâles irradiés vis-a-vis des mâles sauvages<br>**A. Sow**	

**18:00-18:30**	**General discussion**	P. Solano & V. Lejon
**19:30-22:00**	**Reception at IRD representative's house**	

08/10/2010

Time	Scientific programme	Chairmans/animateurs
**08:30-13:00**	**<u>Session 2</u>**<br>**CONSEQUENCES OF MALARIA IN PREGNANT WOMEN AND ASSOCIATED FŒTAL OUTCOMES**	
**08:30-9:30**	**Scientific Conferences**	D. Kinde Gazard & A. Garcia
08:30-09:00	Quels outils pour éradiquer le paludisme?<br>**A. Massougbodji**	
09:00-09:30	Malaria vaccines: now or never?<br>**A. Lutty**	
**09:30-10:45**	**Scientific Session 1**	B. Fayomi & F. Migot Nabias
09:30-09:45	Traitement préventif intermittent (TPI) pendant la grossesse : intérêt de la méfloquine et alternative<br>**V. Briand**	
09:45-10:00	Evaluation du TPI chez la femme enceinte – Mise en œuvre sur le terrain et impact sur le poids de naissance depuis 2005<br>**T. D'Almeida**	
10:00-10:15	Cohorte de Tori-Bossito : caractérisation moléculaire des clones plasmodiaux infectant la mère et l'enfant<br>**C. Dechavanne**	
10:15-10:30	Survenue des premières infections palustres au Bénin : rôle de l'infection placentaire et de facteurs environnementaux<br>**A. Le Port**	
10:30-10:45	Conséquence du paludisme gestationnel sur les réponses cytokiniques à des ligands des TLRS chez les nouveau-nés<br>**K. Gbédandé**	

10:45-11:15 Coffee break
Poster session 2

**11:15-13:00**	**Scientific Session 2**	D. Ogobara & N. Fiévet
11:15-11:30	Antibodies that inhibit binding of *Plasmodium falciparum* infected erythrocytes to CSA correlate with improved pregnancy outcomes<br>**J. Doritchamou**	
11:30-11:45	Prévention du paludisme chez la femme enceinte infectée par le VIH au Bénin : ESSAI PACOME.<br>**L. Denoeud**	
11:45-12:00	Etudes pangénomiques de susceptibilité au paludisme : discordances et conséquences<br>**A. Garcia**	
12:00-12:15	L'infection par *Schistosoma haematobium* affecte les réponses IgG anti-Plasmodium falciparum associées à la protection palustre<br>**D. Courtin**	
12:15-12:30	Stratégies d'Elimination du paludisme basées sur la dynamique saisonnière de transmission dans les pays sahéliens de la CEDEAO: "transmission targeted malaria elimination strategies" in ECOWACS region<br>**O.K. Doumbo**	
**12:30-13:00**	**General discussion**	A. Massogbodji & O. Gaye

13:00-14:00 Lunch

**14:00-18:30**	**<u>Session 3</u>**<br>**INNOVATIVE STRATEGIES FOR CONTROLLING AND MANAGING INSECTICIDE RESISTANT MALARIA VECTORS**	
**14:00-15:00**	**Scientific Conferences**	P. Carnevale & C. Rogier
14:00-14:30	Place de la lutte antivectorielle dans le projet d'élimination du paludisme<br>**M. Akogbéto**	
14:30-15:00	Prospects for use of fungus-based biopesticides in novel strategies of integrated vector management<br>**M. Thomas**	
**15:00-16:15**	**Scientific Session 1**	F. Chandre & L. Djogbénou
15:00-15:15	Situation de la résistance aux pyréthrinoïdes et aux carbamates chez *Anopheles gambiae* dans les sites cotonniers du Bénin en fonction des stratégies de protection appliquées par les producteurs<br>**A. Yadouléton**	

15:15-15:30	Impact des stratégies de protection du coton sur l'homme et l'environnement: sélection de la résistance des vecteurs du paludisme aux insecticides<br>**A. Diabaté**	
15:30-15:45	The complexity of DDT-pyrethroid reistance in *Anopheles gambiae* Giles : an investigation of alternative insecticides for use on bednets and the development of long lasting formulations for indoor residual spraying in malaria endemic settings<br>**R. N'Guessan**	
15:45-16:00	Première évaluation de l'efficacité parasitologique de moustiquaires imprégnées et/ou des bâches plastiques imprégnées vs les aspersions intra domiciliaires à Balombo (Angola)<br>**P. Besnard**	
16:00-16:15	Phase III. Evaluation of insecticide treated Durable Lining in Rural African Houses for control of malaria vectors in western Kenya<br>**R. Allan**	
16:15-16:45	Coffee break<br>*Poster session 3*	

**16:45-18:00**	**Scientific session 2**	T. Baldet & R. N'Guessan
16:45-17:00	Evaluation épidémiologique de la lutte anti vectorielle contre le paludisme<br>**C. Rogier**	
17:00-17:15	Evaluation communautaire de stratégies de lutte anti vectorielle contre le paludisme combinant deux traitements insecticides : étude pré intervention<br>**G. Damien**	
17:15-17:30	Evaluation communautaire de stratégies de lutte anti vectorielle contre le paludisme combinant deux traitements insecticides : étude post intervention<br>**A. Djénontin**	
17:30-17:45	Modéliser le risque de transmission du paludisme dans un contexte de lutte anti vectorielle : enjeux et méthode<br>**N. Moiroux**	
17:45-18:00	Réponse anticorps anti-protéines salivaires d'Anophèles : nouveau biomarqueur évaluant l'efficacité de la lutte contre les vecteurs du paludisme<br>**P. Dramé**	
**18:00-18:30**	**General discussion**	R. Allan & V. Corbel
**18:30-18:45**	**Official closure**	M. Akogbeto
**19:30-00:00**	**Buffet dinner at the Berlin Restaurant in the presence of "Petit Miguelito" and "Les Etoiles du Bénin"**	

RESUMES / ABSTRACTS SESSION 1

Nouvelles stratégies de prévention et de lutte contre les trypanosomoses africaines

New strategies for the prevention and control of trypanosomiasis in Africa

Conférences scientifiques/Scientific conferences

SI_C1. Le diagnostic de la Trypanosomose Humaine Africaine : statu quo ou temps de changement.

V. Lejon

Mots clés : Trypanosomose Humaine Africaine; diagnostic; revue; recherche

Adresse : vlejon@itg.be

Le diagnostic précoce des patients atteints de la Trypanosomose Humaine Africaine (THA) est une stratégie cruciale pour le contrôle de la maladie. Le diagnostic classique de la THA consiste en plusieurs étapes. La détection d'anticorps spécifiques est utilisée comme préliminaire pour retrouver facilement des cas à forte suspicion de Trypanosomes. Ensuite, des tests parasitologiques sont utilisés pour confirmer l'infection. Si l'infection est confirmée, une étape suivante sera de diagnostiquer l'atteinte du système nerveux central, ce qui déterminera le type de traitement. Finalement, pour vérifier si le traitement a été efficace, les patients sont suivis pendant 2 ans, en utilisant les mêmes techniques.

Lors des dernières décennies, les techniques utilisées sur le terrain pour dépister la THA ont à peine changés. Pourtant, elles ont montré certaines limites : les sensibilités et spécificités des tests restent insuffisantes, et la ponction lombaire est considérée comme une procédure invasive. Entretemps, beaucoup d'efforts ont été faits pour développer de meilleurs tests diagnostiqués pour la THA. Pour arriver au terrain, ces nouveaux tests doivent être fiables, simples à faire avec un minimum de matériel spécialisé, rapides et robustes, et d'un coût supportable. L'exposé fait une revue des techniques actuellement utilisées pour diagnostiquer la THA, des nouveaux développements et des recherches en cours sur le sujet.

SI_C2. La lutte anti-vectorielle dans les stratégies de prévention et de contrôle des Trypanosomiases Africaines

P. Solano, M. Camara, I. Sidibé, J.B. Rayaisse, D. Kaba, J. Bouyer, S. Ravel, T. de Meeûs, M. Kagbadouno & G. Cuny

Mots clés : glossines ; lutte anti vectorielle ; génétique éradication ; contrôle

Adresse : philippe.solano@ird.fr

Les glossines, ou mouches tsé-tsé, sont les vecteurs des Trypanosomiases Humaines (THA ou maladie du sommeil) et Animales (TAA) africaines, qui constituent encore un fardeau énorme au niveau de la santé publique et en terme de pertes économiques pour l'Afrique subsaharienne. Les travaux effectués depuis une dizaine d'années permettent maintenant de comprendre la structure des populations de *Glossina palpalis*, vecteur majeur des trypanosomiases en Afrique de l'Ouest, ainsi que les interactions spatiales entre groupes taxonomiques de trypanosomes et espèces de vecteur. *G. palpalis* s'organise

sous forme de sous-populations vivant chacune dans un espace confiné, génétiquement distinctes, de petite taille, échangeant un flux limité de gènes entre elles. Le degré de « fragmentation » de ses populations semble lui-même lié au degré de fragmentation de leur habitat, riverain ou forestier. Cette glossine s'adapte par ailleurs très bien à un habitat fortement urbanisé et est capable de se maintenir en faibles densités suite à des opérations de lutte, ce qui en fait un vecteur toujours redoutable malgré l'explosion démographique africaine et les changements climatiques censés en réduire l'habitat. Les programmes actuels d'élimination de la THA (par l'OMS) et des TAA (sous la bannière de l'Union Africaine par le biais du PATTEC) tiendront compte de ces nouvelles données génétiques, qui contribuent au choix de la stratégie de lutte anti-vectorielle (suppression ou éradication) et des méthodes utilisées (piégeage, SIT). Un état des lieux des actuels projets d'élimination des tsé-tsé et des Trypanosomiases sur le continent africain sera présenté en fonction des objectifs affichés et des premiers résultats obtenus. Il s'agira dans les prochaines années de comprendre la présence et l'absence des Trypanosomiases sur le terrain pour pouvoir prédire leur extension ou régression en tenant compte des interactions tsé-tsé/trypanosome et des prévisions démographiques et climatiques.

Communications orales/ Oral communications

SI_O1. A geographical approach to identify sleeping sickness risk factors in a mangrove ecosystem

F. Courtin, V. Jamonneau, M. Camara, O. Camara, B. Coulibaly, A. Diarra, P. Solano & B. Bucheton

Key words: Sleeping sickness, geography, mobility, settlement, Guinea
Address: courtinfabrice@yahoo.fr

Guinea is the most affected country of sleeping sickness in West Africa. In the littoral of Guinea, from north to south, the active Boffa, Dubreka, and Forecariah foci are found in typical mangrove areas, with prevalence reaching 5 % in some villages. However, only a few studies have been done on sleeping sickness in this particular ecosystem. To provide a better understanding of sleeping sickness transmission and spread in mangrove areas to optimize its control, we have done a spatial followed up of 19 sleeping sickness cases and 19 matched controls in their living areas (at home, in fields and at water points), in the Forecariah focus. All occupational sites and pathways were mapped and then placed in their environmental context. The sleeping sickness cases displayed a significantly broader and more diverse spatial occupation than the controls. They covered double the daily walking distances of controls and had on average two more occupational sites, most of which were located in mangrove forests. Activities with a higher transmission risk (rice culture, attendance of pirogue jetties) were identified as well as high-risk areas and pathways. An entomological control strategy targeting transmission risk areas is proposed. Its implementation in a control programmer would reduce by 86% the efforts needed for a classical vector control programmer throughout the area. Medical surveys set up at specific locations, such as pirogue jetties and high-risk paths, should also enable better targeting of the population at highest risk.

SI_O2. Population genetic structure of *Trypanosoma brucei gambiense* in coastal guinea, control implication.

J. Kaboré, A. McLeod, T. De Meeûs, B. Bucheton, C. Duffy, H.Ilboudo, M. Camara, O.Camara & V. Jamonneau

Key words: *Trypanosoma brucei*; population genetic; human reservoir; tissues; tropism; Guinea
Address : jacqueskabore@yahoo.fr

Little is known on the biological, human and environmental factors that determine the epidemiological feature of human African trypanosomoses (HAT), its relation to animal reservoirs, the variability of clinical manifestations, the complex relationships with its insect vectors (tsetse flies) and human activities. It is for instance unknown why some patients
stay seropositive for several years without any clinical or parasitological manifestation, while others quickly develop the acute phase of sleeping sickness. The causes that determine the anatomical tropism of trypanosomes (blood, lymph node or cerebrospinal fluid) also remain mysterious as some patients can stay in phase 1 (blood and lymphnodes) of the disease for years while other patients quickly enter in phase 2 (cerebrospinal fluid). In the work presented we will try to unravel the role played by the different anatomical locations (blood, lymph node or cerebrospinal fluid) where the parasite can be found on the population
genetic structure of *Trypanosom brucei gambiense* in Coastal Guinea. We will also test for the influence of the serological/parasitological status of the patient: seropositive without parasitological confirmation and confirmed infected (ill) patients. These factors will be studied in relation to geographical location of the three foci of Coastal Guinea (Boffa, Dubreka and Forecariah) from where isolates come from. Taking into account these different relevant factors will also allow us estimating the precise reproductive strategy used by *T b gambiense* (purely clonal or not), which is a key information for understanding the population biology of this pathogenic agent.

SI_O3. Use of immune trypanolysis to optimize epidemiological surveillance and control of sleeping sickness.

V. Jamonneau, H. Ilboudo, J. Kaboré, B. Bucheton, M. Camara, R. Kambire, K. Lingue, V. Lejon & P. Büscher

Key words: *Trypanosoma brucei;* diagnosis; epidemiological surveillance; elimination; West Africa
Address: vincent.jamonneau@ird.fr

Control of HAT in West Africa heavily relies on mass screening of the population at risk for specific antibodies by the Card Agglutination Test for Trypanosomiasis (CATT) where after positive cases are further examined for the presence of trypanosomes in the body fluids. However, a fraction of CATT positive persons cannot be confirmed by parasitological tests and remain untreated. Whereas part of these aparasitemic seropositive individuals may be false positive due to cross-reaction originating from other infections, others are suspected to
be infected with *Trypanosoma brucei gambiense.* CATT is not specific enough to evaluate whether *T. b. gambiense* is still circulating in a given area. The objective of the study was to evaluate the value of the immune trypanolysis test (TL) for characterization of CATT positive subjects and for monitoring HAT elimination in West Africa. TL was performed on plasma collected from all CATT positive persons identified in the frame of medical surveys in several West African HAT foci in Guinea, Côte d'Ivoire and Burkina Faso (REFS study areas) with diverse epidemiological status (active, latent, or historical). All HAT cases were TL+. All subjects living in a non-endemic area were TL-. A significantcorrelation was found between the percentage of TL positive individuals in non parasitologically confirmed CATT positive persons and HAT prevalence. This was not the case with CATT prevalence. TL appears to be a marker for contact with *T.b. gambiense.* TL represents a tool (i) at an individual level to identify among non parasitologically confirmed CATT positive subjects, those who should be followed up, (ii) at a population level, to identify priority areas for intervention, and (iii) in the context of HAT elimination to identify areas free of HAT.

SI_O4. Caractérisation immunologique de la diversité clinique de la THA en Guinée.

H. Ilboudo, R. Bras-Gonçalves, M. Camara, J. Kabore, O. Camara, V. Jamonneau & B. Bucheton

Mots clés : THA ; Diversité clinique ; séropositifs ; Guinée
Adresse : hamidou_ilboudo@hotmail.com

La Trypanosomose Humaine Africaine (THA) ou maladie du sommeil est due à un protozoaire flagellé du genre *Trypanosoma* dont la transmission est assurée par les glossines. Comme pour d'autres maladies parasitaires et, plus généralement, maladies infectieuses, il est observé une importante variabilité de réponses de l'hôte à l'infection. En effet, certains individus développent des pathologies sévères alors que l'infection peut demeurer totalement asymptomatique chez d'autres. La THA évolue chez l'homme en deux périodes. Durant la première phase, le parasite se multiplie dans le sang et la lymphe sans signes cliniques spécifiques associés. La deuxième phase, est déterminée par le passage du parasite dans le liquide céphalo-rachidien (LCR) et se caractérise par des troubles neurologiques qui entraînent la mort du patient en l'absence de traitement. Cependant, certains individus vivant en zone d'endémie, présentent une réactivité immunologique (stable dans le temps) vis-à-vis des antigènes de trypanosomes sans que les parasites puissent être mis en évidence malgré des recherches répétées. Chez ces individus on peut suspecter un phénomène de trypanotolérance comme cela est décrit chez les bovins. L'objectif de cette étude a été de caractériser la réponse immune de ces individus. Les taux d'interleukines IL-12, IL-2, IL-4, IL-5, TNF-α, INF-γ, IL-8, IL-1b, IL-6, IL-10 ont été déterminés dans des plasmas représentatifs de la diversité clinique observée en Guinée (patients en phase 1, patients en phase 2, séropositifs et contrôles sains). Les taux plasmatiques des cytokines IL-1b, IL-10 étaient significativement plus élevés dans le groupe des patients que chez les contrôles. Les séropositifs se caractérisent par des concentrations significativement plus élevées d'IL-8, d'IL-6, d'IL-5 et de TNF-α et par des concentrations significativement plus basse d'IL-1b, d'IL-12 et d'IL-10 comparés au groupe des malades. Les implications sur l'orientation de la réponse immune en termes de contrôle de la maladie seront discutées.

SI_O5. Campagne d'éradication des glossines dans les îles de Loos (République de Guinée) : résultats actuels.

M. Camara, M. Kagbadouno, J. Bouyer, F. Courtin, M.F. Onikoyamou, D. Kaba & P. Solano

Mots clés : *Glossina palpalis* ; lutte anti vectorielle ; isolement génétique ; île
Adresse : moisake65@yahoo.fr

La Guinée, en particulier littorale, est le pays le plus touché d'Afrique de l'Ouest par la THA (Trypanosomose Humaine Africaine, ou maladie du sommeil). Sur les îles de Loos, archipel situé à 4 km de Conakry, le vecteur des Trypanosomoses *G. palpalis gambiensis* a été identifié en fortes densités, alors que des cas de THA ont été signalés jusque dans les années 1980 et qu'une forte activité économique repose sur l'activité d'élevage porcin, menacée par la trypanosomose animale. De plus, une récente étude basée sur la génétique des populations a conclu à un isolement génétique des tsé-tsé de cet archipel par rapport à celles présentes en mangrove continentale.
Face à cette situation, le PNLTHA (Ministère de la Santé) et la DNE (Ministère de l'Elevage) Guinée, en collaboration avec le réseau LTTRN, le MAEE (FSP-REFS), des Instituts de Recherche comme l'IRD, le CIRDES, l'IPR et de compagnies privées (Vestergaard-Frandsen) ont lancé un programme d'éradication des tsé-tsé dans les îles Loos. Pour atteindre ces objectifs, une combinaison des méthodes suivantes est mise en place : traitement des porcs au « pour on », utilisation de filets imprégnés autour des parcs à porcs, utilisation des écrans et pièges imprégnés d'insecticide, pulvérisation d'insecticide au sol limitée aux refuges des tsé-tsé en saison sèche. Les résultats actuels sont prometteurs avec des taux de suppression respectivement égaux à 99,8%, 100%, et 92,5% dans les 3 principales îles de Kassa, Room et Fotoba. Ces résultats sont discutés au vu des notions de suppression et d'éradication, et de leur durabilité.

SI_O6. *Sodalis glossinidius* and vector competence of field tsetse flies in two historical human African Trypanosomiasis (HAT) foci, Southern Cameroun.

O. Farikou, F. Njiokou, T. Asonganyi, P. Simarro, G. Cuny, & A. Geiger

Key words: *Sodalis glossinidiu;* Vectorial competence; Human African Trypanosomiasis; Historical foci; Cameroon
Address: pfarikoumar@yahoo.fr

Endosymbiotic bacteria are found in a wide range of insect taxa, where they provide supplementary nutritional components essential for the survival of insects that feed on a restricted diet such as plant sap or blood. *Sodalis glossinidius* is a maternally transmitted endosymbiont of Glossina and its presence has proved to be involved in the vector competence of tsetse flies by favoring parasite establishment in the midgut and consequently favour trypanosome infection. Our previous investigations shown that the presence of *S. glossinidius* favour trypanosome infection of insectary reared tsetse flies and could possibly be of major factor in the transmission process of the disease but not an absolute condition. Given that, flies multiplying in the insectary may undergo specific selection pressure, the possibility that field environmental conditions of HAT foci may lead to alternative results could not be excluded. Therefore, epidemiological surveys have been conducted in two historical and still active HAT foci, Bipindi and Campo, in Cameroon. 7 flies were randomly chosen among the flies trapped in each of the six villages. After species identification, flies were dissected and the presence of both the symbiont and the trypanosome was tested by specific PCR methods. Furthermore, the species of the fly infecting trypanosomes were identified. Statistical significant differences were recorded between the prevalence of *S. glossinidius* and that of flies infection by the trypanosomes. The prevalence of, respectively, *S. glossinidius* and fly infection, differed also significantly between the two foci. Of interest was that, despite the high difference in prevalence between the two foci, the rate of infected flies harbouring the symbiont was similar in both foci. This result suggests fly's infection to be favoured by the presence of the symbiont; according to the recorded data, the probability for flies harbouring the symbiont to be infected by the trypanosome was about 3 fold higher than for flies devoid of the symbiont. In addition, all the flies that were infected by *T. brucei gambiense* (responsible for the HAT), were symbiont positive. This finding suggests that the symbiont could constitute an interesting target for controlling parasite transmission and thus the sleeping sickness disease. So, such studies on tripartite interactions should be pursued and developed with the objective of controlling fly vectorial competence and possibly sleeping sickness.

SI_O7. A new transmission Risk Index of Human African Trypanosomiasis and its evaluation in the Fontem focus

G. R. Njitchouang , F. Njiokou , H. N. Djeunga , T.Asonganyi , P. F. Moundipa , G. Cuny & G. Simo

Key words: HAT; ADT; Transmission Risk Index; *Trypanosoma brucei gambiense*; Fontem
Address: njitchouang@yahoo.fr

In order to develop a new transmission risk index to better estimate the transmission risk of sleeping sickness, two entomological surveys were carried out at the end and at the beginning of the rainy season. A total of 2695 tsetse flies, all belonging to the sub-species *Glossina palpalis,* were trapped and 1596 dissected. PCR allowed the detection of
75 (4.7%) *Trypanosoma brucei s.l.* midgut infections and 9 (0.6%) of them were *T. b. gambiense* group 1; this finding suggests the circulation of human infective parasite in the Fontem focus. The apparent density (the number of flies per trap per day: ADT) was 0.66 during the first survey and 4.85 during the second one. Of the 1596 flies dissected, 184 blood

meals were identified and 55.10% of them were from pigs, 25.23% from humans, 17.6% from wild animals and 1.2% from goats. Among the blood meals taken from humans, 98.5% were in sites distant from pig sties whereas only 1.5% was taken near pig sties. At the end of the rainy season, the ADT was low and similar between biotopes distant and close to pig sty. However, the risk of transmission was higher in the biotopes distant from pig sties. This suggests that pigs constitute a protective barrier for human. Comparing the new Transmission Risk Index (TRI) with the previous one, we observed a strong statistical correlation between the two TRI, suggesting that both can be used to evaluate the level of transmission of the disease. Remarkably, the new index take into account some parameters involved in the transmission of the disease, suggesting that it might be better to evaluate the risk of transmission. At the beginning of the rainy season, the ADT and the transmission risk indices were generally higher due to the relative abundance of flies. These two parameters were higher in sites near pig sties because during this period, the relative high temperatures might favour the propagation of odours from the pig sties that attract tsetse flies.

SI_O8. Prospects of fungus-contaminated device for the control of tsetse fly.

N. K. Maniania, S. Ekesi, A. Odulaja, M. A. Okech & D.J. Nadel

Key words: Entomopathogenic fungus; autodissemination; *Glossina fuscipes*; control

Address: nmaniania@icipe.org

The prospects of the fungus *Metarhizium anisopliae* applied in contamination devices (Cds) to control tsetse fly *Glossina fuscipes fuscipes* was tested in a field experiment in Lake Victoria from March 1999 to March 2000. One hundred and sixty (160) pyramidal traps

mounted with Cds were deployed along the lakeshore and rivers on Mfangano. Cds were loaded with 1.5-2.0g of dry conidia/Cd. In the second island, Nzenze Island, three pyramidal traps fitted with plastic bags were deployed and served as the conventional trap and kill Population suppression method. A third island, Ngodhe Island, remained untreated

and served as a control. Fungal conidia were recharged monthly by removing the old ones. Plastic bags were also changed monthly. The apparent changes in population density were monitored weekly using biconical traps set at random on the three islands. To assess the

incidence of *M. anisopliae* in tsetse flies in Mfangano Island, flies captured during monitoring were maintained in the laboratory and their mortality recorded. Fly population was reduced to 82.4 and 95.8% relative to untreated control in Mfangano and Nzenze islands, respectively, during the experimental period. Compared to fungus-treated island, the

number of flies caught in monitoring traps increased considerably in trap kill treatment 5 months after the treatments were removed. The incidence of *M. anisopliae* in fly populations was low during the 12 weeks following the initiation of the experiment but increased afterward up to the termination of the treatment. Fungus was still present in fly

populations 5 months after the end of the treatment although the incidence was low. The results of this study have showed that fungus used in a contamination device can give a control comparable to trap and kill technology.

SI_O9. Importance de la morphométrie géométrique dans le choix de la stratégie de lutte contre les tsétsés.

D. Kaba, P. Solano, G. Acapovi-Yao, K. Allou, A. Diarrassouba, M.T.Seck, J. Bouyer, S. Ravel, K.E. NGoran & J-P. Dujardin

Mots clés : morphométrie géométrique; tsé-tsé; lutte; stratégie

Adresse : kaba_dramane@yahoo.fr

Les trypanosomoses demeurent un problème préoccupant de santé publique et de développement socio-économique en Afrique sub-saharienne, où elles affectent la santé humaine et animale, réduisent l'utilisation des terres et engendrent de plus en plus de pauvreté. La lutte contre le vecteur, la mouche tsé-tsé, reste un des moyens sûrs pour contrôler ces maladies. Or, si des outils de lutte anti vectorielle efficaces et simples existent contre le vecteur, le choix de la stratégie la plus efficace pour un résultat plus durable est en revanche discuté entre l'éradication et la suppression. L'éradication étant la création de zones exemptes de tsé-tsé tandis que la suppression consiste à réduire les densités de glossines pour casser le cycle de transmission de la maladie. Telle que définie, l'éradication bien que plus coûteuse et difficile à obtenir, serait plus rentable, si les populations ciblées sont isolées par rapport aux populations adjacentes afin d'éviter une ré-invasion de la zone assainie. Les marqueurs ADN microsatellites sont capables de détecter des populations isolées, mais sont relativement onéreux et non accessibles à tous. La morphométrie géométrique, appliquée aux ailes de glossines, se présente alors comme une alternative moins couteuse et plus accessible. Dans le but de tester la capacité de ce nouvel outil, la morphométrie géométrique, à détecter les populations isolées et à aider les programmes nationaux de lutte au choix de la stratégie et de la méthode de lutte à appliquer pour obtenir un assainissement plus durable voire définitif des zones ciblées. Notre étude s'est déroulée dans deux pays d'Afrique de l'ouest, au Sénégal dans la région des Niayes et de Missirah, et en Côte d'Ivoire à Abidjan, sur les 3 sites du Banco : la forêt du banco et ses reliques forestières à l'Université d'Abobo-Adjamé et au Zoo. Elle suggère que la morphométrie géométrique montre des différences significatives entre des populations isolées (cas de la région des Niayes au Sénégal), et ne se différencie pas des populations en absence d'isolement (cas de la zone du Banco à Abidjan). Cette approche est rapide et peut aider ainsi au choix de stratégies de lutte adaptées et différentes dans ces deux cas.

SI_O10. Test de compétitivité de *Glossina palpalis gambiensis* mâles irradiés vis-à-vis des mâles sauvages.

A. Sow, I. Sidibé, Z. Bengaly, J. Bouyer, P. Solano, Z. Bancé, & P. V. den Bossche

Mots clés : *Glossina palpalis gambiensis*; Technique de l'insecte stérile; PATTEC ; BurkinaFaso
Adresse : wosamada@yahoo.fr

Des lâchers expérimentaux de mâles irradiés *Glossina palpalis gambiensis* ont été effectués de janvier à février sur une distance de 3km le long de la rivière Layessa. Sur les 16000 mâles irradiés lâchés, 15008 (93,80%) ont effectivement pris leur envol. Des enquêtes entomologiques périodiques ont permis de déterminer les ratios des mâles irradiés et les mâles sauvages, les densités apparentes des mâles lâchés, des mâles et femelles sauvages, les taux d'avortements et les taux de remplissages des spermathèques. Ces enquêtes ont révélé qu'il y avait une différence significative entre les taux d'avortements des femelles disséquées avant les lâchers (3,3%) et celles après les lâchers. Ainsi de la deuxième à la dixième semaine, les taux d'avortement étaient restés supérieurs au taux d'avortement de la période avant les lâchers avec une différence statistiquement significative (p=0,000). Les taux de remplissages n'ont montré aucune différence significative ni entre les différentes classes d'âge des glossines, ni entre les femelles disséquées avant et après les lâchers. Par contre, les taux de remplissage des spermathèques furent plus importants chez les femelles sauvages que chez les femelles vierges de l'insectarium accouplées avec des mâles irradiés (p=0,015). Les mâles irradiés ont montré une bonne dispersion, les pièges de contrôle ont capturé presque les mêmes proportions de mâles irradiés. La demi-vie des mâles irradiés a été assez faible soit 4,58 ± 1,26 jours. Tout le long de l'expérience, le ratio mâles irradiés/ mâles sauvages a été inférieur à 2. Cependant, l'impact en terme d'avortement des jeunes pares a été significative. Aussi les mâles irradiés de l'insectarium du CIRDES, peuvent être utilisés dans la Technique de l'insecte stérile pour l'élimination des mouches tsétsé dans la zone d'intervention de PATTEC Burkina.

Posters

SI_P1. Caractérisation de la réponse cellulaire *ex-vivo* chez des individus infectés par *Trypanosoma brucei gambiense.*

H. Ilboudo, V. Jamonneau, P. Holzmuller, G. Cuny, A. Garcia, B.Bucheton & D. Courtin

Mots clés : *Trypanosoma brucei gambiense*; maladie du sommeil; susceptibilité; cytokines
Adresse : david.courtin@ird.fr

La description classique de la THA ne correspond pas exactement à ce qui est observé sur le terrain. En effet, dans les foyers de THA, les équipes de dépistage sont confrontées à une situation complexe faisant intervenir plusieurs catégories d'individus : des sujets sains qui vivent depuis longtemps dans le même environnement que les malades, des sujets malades présentant des formes chroniques classiques de THA à *T. b. gambiense*, des sujets malades semblant présenter des formes différentes de THA à *T. b. gambiense*, allant de formes asymptomatiques à des formes aiguës, et des sujets présentant une réponse positive lors des tests sérologiques (diagnostic indirect) chez lesquels des recherches répétées de parasites restent négatives (sujets séropositifs). Etant donné l'importance des cytokines, au niveau immunologique et génétique, dans le contrôle de la THA, notre objectif a été de comparer les niveaux de cytokines d'intérêt sécrétés par les cellules issues des différents groupes d'individus. Matériel et méthodes : L'étude a été réalisée en Côte d'Ivoire dans les foyers de Sinfra et Bonon. Nous avons déterminé par ELISA les niveaux d'IL-6, IL-10, TNF-a et IFN-g, avant et après stimulation par des trypanosomes, des cellules du sang total issues d'individus : (1) témoins (n=23); (2) anciennement malades (en phase 1 (n=18) ou en phase 2 (n=21)); (3) séropositifs (n=10) et (4) asymptomatiques (n=8). Résultats : Les cellules des individus asymptomatiques ont sécrété, après stimulation avec des trypanosomes, un niveau IFN-g significativement plus élevé que celui des cellules issues des autres groupes d'individus (p=0,0001, Kruskall-Wallis). Il est également important de noter que la production d'IFN-g par les cellules des individus séropositifs, après stimulation, est significativement plus élevée que celle du groupe témoin (p=0,0093 ; Kruskall-Wallis) et du groupe des anciens malades en phase 1 (p=0,044 ; Kruskall-Wallis).

Ces résultats confirment que la THA entraîne une forte stimulation du système immunitaire avec des sécrétions importantes de cytokines. Ils indiquent aussi que l'IFN-g pourrait jouer un rôle important dans le contrôle de la maladie et dans le phénomène de séropositivité sans confirmation parasitologique. Ils suggèrent donc l'implication de

L'immunité dans cet aspect particulier de l'épidémiologie de la THA.

SI_P2. Does resistance to pentamidine offer a new challenge in the fight against human african trypanosomiasis?

L. K. Tongué, P.Mallaye, A. Diarra, R. Mimpfoundi, M.P. Barrett & F.J. Louis

Key words: Resistance; pentamidine; Human African Trypanosomiasis; Mandoul; Chad
Address: lisette_oceac@yahoo.fr

The control of Human African trypanosomiasis (HAT) depends to a large extent on the availability of successful drugs. The second stage of the disease is particularly difficult to manage because current drugs are highly toxic (melarsoprol) or difficult to administer (eflornithine). Drugs used for the first stage are less toxic and resistance of *T. b. gambiense* to pentamidine in the field is rare. Indeed, pentamidine has been used for over fifty years against stage 1 with few reports of resistance. However, we have recently observed a significant incidence of treatment failure using pentamidine in the Mandoul focus of Chad. A follow-up of previously treated patients using the method of white blood cell count was performed during a mass screening survey. Amongst 54 patients treated with pentamidine,

22 came for follow-up and the white blood cell count (WBC) of 18 of them were found in increase. These relapses cases were treated with melarsoprol; to the next follow-up, only 11 of those melarsoprol treated cases presented and were examined. Their WBC was in decline but a PCR analysis (using the *T. brucei* sl (177 bp) TBR primers) amplified trypanosome DNA from the blood of the majority of these. To date, a mechanism for resistance to pentamidine has not been identified in field isolates, although an association with the loss of the P2 aminopurine transporter has been documented in the laboratory. We are currently analyzing the status of the TbAT1 gene that encodes the P2 transporter, in the field isolates, aiming to understand the problem of drug resistance and a view to managing its spread.

SI_P3. Long term follow-up of sleeping sickness patients refusing treatment and aparasitemic seropositive individuals: implications for control.

V. Jamonneau , J. Kaboré , H. Ilboudo, D.Courtin, A. Garcia , B. Coulibaly , P. Solano , P. Büscher & B. Bucheton

Key words : *Trypanosoma bruce ;* diagnosis; seropositive; individual susceptibility; Côte d'Ivoire
Address: vincent.jamonneau@ird.fr

During control programs carried out in the Sinfra (1995 1997) and Bonon foci (2000 2004) in Côte d'Ivoire, several hundred HAT cases but also seropositive and aparasitemic individuals were identified. While most of HAT cases have been treated, some of them refused immediate treatment. In order to convince them to be treated, they were regularly
followed up. Serological (CATT and trypanolysis test), parasitological, and clinical investigations were performed on these occasions to assess the evolution of the disease. Seropositive individuals were also followed up. Results of these long term follow ups show two patterns of HAT evolution: (i) a classical evolution to the second stage of the disease
leading to treatment or death or (ii) a long term asymptomatic period with progressive disappearance of parasites. This disappearance was associated either with a serological response progressively becoming negative or with a strong and stable serological response i.e. such as seropositive individuals. Among the seropositive but aparasitemic individuals followed up, whereas the serological tests became progressively negative for part of them, others maintained a long term strong serological response to the CATT and/or trypanolysis test. Among them, only a few subjects have been parasitologically confirmed. These results are in agreement with the existence of an individual susceptibility to infection (human trypanotolerance or self cure). Furthermore, they also suggest that seropositive individuals may represent potential parasites reservoirs and should not be neglected in the context of HAT elimination strategies.

SI_P4. Evaluation de la réponse anticorps dirigée contre les antigènes salivaires de tsétsé chez l'homme.

E. Dama, M. B. Somda, H. Ilboudo, A. Poinsignon, F. Remoue, M. Camara, V. Jamonneau, R. Kambire, Z. Bengaly & B. Bucheton.

Mots clés : Salive de glossine; Contact homme/glossine; Marqueur d'exposition
Adresse : dama_emilie@hotmail.fr

La trypanosomose humaine africaine (THA), est causée par deux sous-espèces de *Trypanosoma brucei* transmises par la mouche tsé-tsé. A l'heure actuelle, la lutte contre la THA repose principalement sur deux volets : le dépistage-traitement des malades et la lutte anti-vectorielle. D'une part, les tests diagnostiques utilisés sur le terrain manquent de

spécificité et de sensibilité et d'autre part, l'évaluation entomologique par la technique de piégeage est lourde et très coûteuse, ce qui rend difficile la couverture de vastes zones. L'objectif de cette étude est de développer un nouvel outil basé sur la détection chez l'homme
d'anticorps IgG anti-salive de glossines permettant l'évaluation direct du contact homme-glossines. L'échantillonnage des plasmas analysés a porté sur 256 individus provenant de deux foyers actifs de la THA en Guinée Conakry (Forécariah, Dubreka-Boffa) et de deux anciens foyers au Burkina Faso (Loropéni, Batié). Parmi ces individus il y avait 115 sains, 64 malades et 35 suspects sans confirmation parasitologique. Notre cohorte de témoins négatifs était composée de 18 individus de la ville de Bobo-Dioulasso. Le test ELISA indirect anti-salive de glossines a été effectué sur tous les plasmas. Comme résultats nous avons noté en premier lieu des différences significatives de l'intensité de la réponse anti-salive dans les différents foyers étudiés. Cette réponse était élevée et comparable dans les populations des 2 foyers actifs de Guinée et celle de Batié, contrairement à celle des populations de Bobo et de Loropéni au Burkina qui présentent une faible réactivité sérologique aux antigènes salivaires de glossines. Par ailleurs, une relation entre la fréquentation quotidienne des cours d'eau pour l'approvisionnement en eau et une réponse anti-salive élevée a été établie dans la zone de Batié, un ancien foyer de la THA qui abrite toujours des glossines. De plus, il a été noté une réponse significativement plus faible chez les malades de la Guinée en deuxième phase avancée. En conclusion, nous retenons que la réponse anti-salive des glossines est un bon marqueur sérologique d'exposition aux piqûres des glossines mais manque de spécificité dû à l'utilisation de la salive totale. Il convient de l'améliorer par l'utilisation de protéines salivaires immunogènes plus spécifiques.

SI_P5. Immunoprotective potency of gamma irradiated glossina-borne *trypanosoma brucei* against *Trypanosoma brucei* infection in guinea pigs.

E. I. Awaridhe, M. I. Balarabe & I. H. Nock.

Key words: Gamma irradiation; *Trypanosoma brucei*; infection; Guinea pigs; immunity.

This study determined the immunoprotective potency of gamma irradiated Glossina borne *Trypanosoma brucei* against *Trypanosoma brucei* infection in Guinea pigs. *Trypanosoma brucei* infective *Glossina palpalis* flies were exposed to doses of gamma irradiation (5000, 10000, 15000cGy). The irradiated infected flies were fed on uninfected Guinea pigs and twenty days post the infective Glossina bites, the animals were screened for bloodstream trypanosomes before each was challenged, by syringe passage, with 1.1 x 106 unirradiated
trypanosomes (*T. brucei*). Control Guinea pigs included those exposed to the bites of the irradiated infective flies but not challenged with unirradiated trypanosomes and those challenged with 1.1 x 106 unirradiated trypanosomes only (positive control). Parasitaemia was monitored daily in all the Guinea pigs by microscopic examination. The Guinea pigs fed upon by flies exposed to gamma irradiation at 5000cGy developed infection 10 days post-challenge with unirradiated trypanososmes. Guinea pigs fed upon by flies exposed to gamma irradiation of higher doses (10000, 15000cGy) developed immunity to subsequent *T. brucei* challenge showing no parasitaemia even after 60 days post challenge. Similarly, all control
Guinea pigs exposed to irradiated flies but not challenged with trypanosomes failed to show any parasitaemia; however the positive control set was parasitaemic 8 days post challenge with trypanosomes. The results suggest that metacyclic trypanosomes in the Glossina vector when exposed to high doses of gamma irradiation (10000. 15000) are capable of conferring immunity against *Trypanosoma brucei* infection.

SI_P6. Approche préconisée pour la lutte contre la THA en milieu urbain et périurbain

P. D. Mansinsa, V. B. Kande & E. M. Mwamba

Mots clés : Trypanosomiase Humaine Africaine ; risque de transmission ; lutte anti vectorielle ciblée; Dépistage actif; Dépistage passif
Adresse : diabakana@yahoo.fr

Depuis près de deux décennies, on observe une recrudescence de la trypanosomiase humaine africaine (THA) dans les foyers historiques. On observe également une émergence des foyers présentant un contexte épidémiologique nouveau en milieu urbain En République Démocratique du Congo, le Programme National de lutte a pu expérimenter une approche qui lui a permis de réduire l'endémicité dans le milieu en s'attaquant au problème sous trois volets. Un dépistage actif intensif dans les poches où le problème s'est manifesté avec acuité, une responsabilisation plus accrue des structures de santé de base et une lutte anti vectorielle ciblée après une enquête qui a pu déterminer les endroits à risque élevé de transmission de la THA en milieu urbain et périurbain. Cette approche à permis de réduire l'endémicité à un niveau compatible avec une vie productive normale des populations urbaines menacées par la maladie.

SI_P7. Xénodiagnostic expérimental de la trypanosomose humaine africaine (THA).
W. T. C. Michel, S.Philippe & B. Bucheton

Mots clés : Xénodiagnostic expérimental, THA, Glossines, Trypanosomes
Adresse : claudetoukam@yahoo.fr

En zone d'endémie de la Trypanosomose Humaine Africaine (THA), il existe des suspects positifs en sérologie chez lesquels le diagnostic parasitologique ne permet pas la détection du parasite. Ces sujets n'étant pas traités, ils posent un problème fondamental au niveau individuel (évolution mortelle de la maladie) et au niveau épidémiologique (ils sont de potentiels réservoirs de trypanosomes). L'objectif de notre étude était d'actualiser le xénodiagnostic de la THA en infectant expérimentalement des animaux (rongeurs, bovins, porcs) par T. congolense et *T. brucei gambiense* sur lesquels des glossines (*Glossina morsitans submorsitans* et *G. palpalis gambiensis*) de l'élevage du CIRDES étaient nourries lors de faibles parasitémies. Nos résultats ont montré que : i) les glossines s'infectent mieux avec les trypanosomes si elles sont nourries directement sur l'hôte, que si elles sont nourries sur un système de membrane ; ii) la colonie de *G. p. gambiensis* montre une compétence vectorielle très faible; iii) le jour optimal de mise en œuvre du xénodiagnostic se situe à J2 et J3 post-repas infectant. ; iv) *T. b. gambiense* n'a pu être mis en évidence que chez *G. m. submorsitans* avec l'infection sur porc, jamais chez *G. p. gambiensis* (sur plus de 1 000 disséquées). De ces premiers résultats il ressort qu'il est envisageable d'appliquer le xénodiagnostic de la THA sur le terrain pour la recherche.

SI_P8. La trypanosomiase humaine africaine au Sénégal (1900-1977) : Approche multidisciplinaire pour une identification des régions à risque.
Syll Massamba

Aujourd'hui la trypanosomiase représente toujours un problème de santé publique dans quelques pays d'Afrique subsaharienne, même si on reconnait de manière générale que la situation s'est nettement améliorée à l'instar de l'Afrique de l'ouest. Cependant la situation dans la sous région reste floue puisque très peu, voire pas d'informations sont disponibles pour 6 pays, comme le Sénégal qui autrefois abritait des foyers actifs de trypanosomiase. En plus de nombreuses régions d'Afrique occidentale n'ont pas été visitées depuis les indépendances.
Au Sénégal l'histoire de la THA remonte à la période coloniale. Les premiers cas de trypanosomiase furent signalés sur la Petite Côte par Sice qui les décrit sous le nom de "Nélawane". De nombreux cas furent observés de 1900 à la veille des indépendances, dans les régions contaminées comme le Delta du Sénégal, les Niayes, la Petite Côte, la Casamance et le Sénégal oriental. La maladie avait presque

disparu dans les années 60, période à laquelle elle était alors considérée comme résiduelle. Le dernier cas enregistré au Sénégal date de 1977 chez un jeune homme de 18 ans né à Mbour un ancien foyer actif. Depuis lors, aucun cas de maladie du sommeil n'a été signalé, mais aussi aucun programme de surveillance épidémiologique n'a été mis en place. Seul un projet de lutte contre les glossines dans les Niayes et une partie de la petite côte est en cours d'exécution.
Cependant après avoir revisité les principaux foyers historiques du Sénégal, nous avons étudié la distribution et les densités de glossines en passant par le degré de développement des aménagements hydrauliques ainsi que le réseau hydrographique et la pluviométrie dans les régions autrefois contaminées par la THA. L'analyse de ces différents facteurs en plus du lien migratoire avec les pays infectés ont permis d'identifier les régions à risque de THA à savoir : la Basse et la Haute Casamance, l'embouchure du Sine Saloum, la zone de Kédougou et les Niayes. Ainsi une réactualisation des données épidémiologiques et entomologiques s'impose.

SI_P9. Sheep, goats and pigs as potential reservoir hosts of *Trypanosoma brucei* gambiense in sleeping sickness foci in Cameroon.

H. Nimpaye, F. Njiokou, T. Njine, T. Asonganyi, G. Simo, G. R. Njitchouang, G. Cuny & S. Herder

Key words: PCR; sleeping sickness foci; domestic animal reservoir; *T. brucei* s.l. *T. b. gambiense* ; South-Cameroon
Address: msanze2@yahoo.fr

To understand the implication of domestic animals in the epidemiology of sleeping sickness in four Human African Trypanosomiasis (HAT) foci (Bipindi, Campo, Doumé and central sub focus of the Fontem focus) with different epidemiological features, 875 domestic animals were sampled in 24 villages. Blood was collected from 267 sheep, 264 goats, 307 pigs and
37 dogs and analysed using parasitological (QBC®), immunological (LiTat 1.3 CATT®) and molecular (PCR) tests. The QBC® test revealed trypanosomes in 34 (3.88%) animals while 194 (22.1%) were sero-positive to the antigen LiTat 1.3 with the lowest value in dogs. Specific primers allowed the detection of 174 (19.88%) domestic animals habouring T. brucei s.l DNA. This species complex was found in each of the four domestic animal species and in the four sleeping sickness foci. The infection rates of T. brucei s.l were significantly differed between the animal species and foci ($p < 0.0001$). Of the 174 domestic animals habouring T. brucei s.l DNA, 6.74% of sheep, 3.03% goats and (0.32%) of pigs were infected by *T. b. gambiense.* The DNA of this trypanosome was found in 4.83% of domestic animals from the Campo focus (Ipono and Akak), 3.92% from the Bipindi focus (Bidjouka and Ebimimbang), 2.59% from central sub focus of the Fontem focus (Nsoko) and none from Doumé focus. The *T. b. gambiense* infection rates were significantly differed between the animal species ($p = 0.0001$), but any significantly different was observed between the three foci where *T. b. gambiense* was identified ($p = 0.51$). The detection of *T. b. gambiense* in domestic animals
from the three foci correlated with the intensity of HAT transmission and was in line with results of medical survey where HAT patients were detected in the same villages, suggesting the probable implication of domestic animals in the HAT epidemiology in Cameroon. To improve the control of the HAT, potential host animals of *T. b. gambiense* should be taken into account in all strategies of HAT control.

SI_P10. Analysis of the domestic animal reservoir at a micro-geographical scale, the fontem sleeping sickness focus (south-west Cameroon)

G. R. Njitchouang , F. Njiokou , H. C. N. Djeunga , P. M. Fewou , T. Asonganyi , G. Cuny & G. Simo

Key words: domestic animals, *T. b. gambiense*, sleeping sickness, animal reservoir
Address: njitchouang@yahoo.fr

To better understand the epidemiology of sleeping sickness in two Human African Trypanosomiasis (HAT) sub foci (central and northern sub foci) of the Fontem focus where diversity in the prevalence of *T. b. gambiense* was reported in domestic animals and man, 397 domestic animals were sampled in eight villages. Parasitological tests revealed
trypanosomes in 86 (21.60%) animals. The CATT test was positive in 254 (64%) animals with the lowest value in dogs. The PCR test revealed *T. b. gambiense* in 11.55% of pigs, 3.45% of goats and 15.38% of sheep. The *T. b. gambiense* infection rates were not significantly different between the two sub foci. However, *T. b. gambiense* was found in animals from all villages of the Northern sub focus while only animals from Menji and
Nsoko (Central sub focus) revealed this infection. The detection of *T. b. gambiense* in animals of the central sub focus was in line with results of medical surveys where HAT patients were detected in the same villages. The absence of patients in the northern sub focus despite the circulation of *T. b. gambiense* in animals from all villages of this sub focus since several years is surprising and needed more investigations.

SI_P11. Présence de vecteurs potentiels des trypanosomoses dans la ville d'Abidjan : cas de diptères stomoxyinae.

K. P. Yao, K. E. Ngoran & G. Duvallet

Mots clés : Trypanosomoses, vecteurs mécaniques, Stomoxes, zones anthropisées
Adresse : patrick.yao@univ-cocody.ci

En Afrique, les trypanosomoses sont causées par des protozoaires appartenant au genre Trypanosoma et le vecteur le plus célèbre connu à ce jour est la glossine ou mouche Tsé-tsé. D'autres vecteurs interviennent cependant dans l'épidémiologie de ces maladies ; il s'agit
de vecteurs dits mécaniques. Dans ce dernier groupe, les plus importants reconnus sont des insectes, les tabanides et les Stomoxes. Leur rôle de potentiels vecteurs de zoonoses, plus particulièrement de la trypanosomiase a été maintes fois signalé. Contrairement aux deux espèces de Diptères Stomoxiynae rencontrées en 2005 dans le nord de la Côte d'Ivoire, une étude menée dans la bande forestière au cœur de la ville d'Abidjan (parc zoologique d'Abidjan (Zoo), forêt à l'intérieur de l'université de d'Abobo-Adjamé (UAA) et forêt du
Banco(Banco)) a révélé la présence de 7 espèces et sous-espèces de Stomoxes. Il s'agit de : *Stomoxys calcitrans, Stomoxys Niger Niger, Stomoxys Niger bilineatus, Stomoxys oméga, Stomoxys taeniatus, Stomoxys pallidus* et *Stomoxys sitiens.* Parmi ces espèces, une seule a été observée dans les 3 zones et dans presque tous les sites de capture ; il s'agit de *S. Niger*, représentée par 2 sous espèces. *S. n. Niger* a été observée dans 87% des sites de capture, représentant ainsi la sous-espèce la plus répandue dans la bande forestière d'Abidjan. Deux espèces ont été moyennement observées ; ce sont *S. calcitrans* et *S. omega*, respectivement dans 40 et 47% des sites de capture. *S. taeniatus, S. sitiens* et *S. pallidus* sont des espèces que l'on pourrait qualifier de minoritaire dans la bande forestière, objet de notre étude. L'analyse des données en rapport avec le niveau d'anthropisation de chacune des zones de prospection indique que *S. n. Niger* est une espèce ubiquiste, se retrouvant aussi bien en zone à forte présence humaine (Zoo et UAA) qu'en zone caractérisée par une forêt dense faiblement anthropisée (Banco). *S. oméga* est également présente dans toutes les zones mais avec une préférence pour la zone forestière dense. Quant à *S. calcitrans*, elle se rencontre dans une forêt plus clairsemée avec une forte présence humaine.

SI_P12. Epidemiology of human african trypanosomosis in the Forecariah mangrove focus (guinea): clinical and diagnosis

B. Bucheton, H. Ilboudo, O. Camara, F. Courtin, J. Kabore, B. Coulibaly, L. NDri, P. Büscher, M. Camara & V. Jamonneau

Key words: Human African Trypanosomiasis Epidemiology Follow-up
Asymptomatic carriers
Address : bruno.bucheton@ird.fr

The mangrove area in Guinea is probably the most affected focus of *Trypanosoma brucei gambiense* (Tbg) HAT in West Africa. Although most cases these last years were reported from the Dubreka focus north from Conakry, few information were available from the Forecariah area, south of the country. The results of medical surveys performed between
Nov 2007 and Oct 2008 indicate that although the disease appears less epidemic than in the Dubreka focus, HAT also occurs in Forecariah with several geographically defined areas where transmission seems to be active. A diagnosis specificity of HAT in the Forecariah area compared to the Dubreka and Côte d'Ivoire foci is that almost all patients have parasitologically positive lymph node aspirates whereas almost all are negative to mAECT. In addition to the 28 HAT patient diagnosed in the frame of this study, 32 serological suspects with CATT plasma titers > 1/8 but no parasitological confirmation were identified. Follow up of these subjects by PCR (Tbr1/Tbr2) and by the Trypanolysis (TL) serological test (a Tbg specific test) was performed to clarify their role in the epidemiology of HAT. PCR positivity was higher in TL+ than in TLserological suspects (50% vs. 18% respectively) and whereas CATT plasma titers decreased both in treated HAT patients and TL- serological suspects, TL positive suspects maintained high serological CATT titers
throughout the follow up and three individuals were confirmed as HAT patients by parasitological tests. These results indicate that TL positive serological subjects are asymptomatic carriers and should thus be taken into account in the frame of HAT control or elimination programs

RESUMES / ABSTRACTS SESSION 2

Conséquences du paludisme chez la femme et le nouveau-né

Consequences of malaria in pregnant women and associated fœtal outcomes

Conférences scientifiques /Scientific conferences
SII_C1. Quels outils pour éradiquer le paludisme ?
A. Massougbodji

SII_C1. Malaria vaccines: now or never?
A. J. F. Luty

Key words: Malaria; *P. falciparum*; vaccines; pregnancy
Address: adrianluty@ymail.com

I will discuss past and current efforts aimed at developing a vaccine for Plasmodium falciparum malaria. Currently the most advanced candidate, RTS, S, developed by GSK, originally in collaboration with the US Army, is now in a multi-centre Phase III trial across sub-Saharan Africa. This is designed as an anti-infection vaccine and is intended primarily to protect infants through immunization at or just after birth. Other efforts aimed at developing an anti-disease vaccine are less well-advanced as the most promising candidates have encountered problems of lack of efficacy in early stage (Phase II) clinical trials in the field. Separately, the knowledge gained specifically about malaria during pregnancy over the last decade has led to the widely-held belief that a vaccine designed to protect mothers and their unborn babies from the effects of placental infection with *P. falciparum* is a plausible option. I will discuss recent progress in this particular field.

Communications orales / Oral communications
SII_O1. Traitement préventif intermittent (TPI) pendant la grossesse : intérêt de la méfloquine et alternative.
V. Briand, H. Kossou, B. Fayomi, P. Ayemonna, N. Fievet, A. Massougbodji & M. Cot

Mots : Paludisme; Grossesse; Prévention; Essai clinique randomisé
Adresse : valerie.briand@gmail.com

En Afrique sub-saharienne (ASS), le paludisme gestationnel est une cause majeure de faible poids de naissance (FPN) et d'anémie maternelle. Sa prévention repose sur le traitement préventif intermittent (TPI) par la sulfadoxine-pyriméthamine (SP), l'utilisation de moustiquaires imprégnées d'insecticide et le traitement des cas symptomatiques. Toutefois, face à l'augmentation des résistances à la SP en Afrique, l'évaluation d'autres molécules utilisables dans le cadre du TPI est devenue nécessaire et urgente. Entre 2005 et 2008, nous avons mené au Bénin, dans la ville de Ouidah, le premier essai clinique évaluant la méfloquine (MQ) pour le TPI. L'essai a été conduit sous la forme d'un essai d'équivalence, randomisé et ouvert. Les femmes ont reçu soit deux doses de SP (1500/75mg) soit deux doses de MQ (15mg/kg), au 2nd et 3eme trimestre de la grossesse. Le principal critère de jugement était le pourcentage de faible poids de naissance (<2500g). Au total, 1601 femmes ont été randomisées

(802 (MQ) et 799 (SP)). Il y a eu 5% de perdues de vue et 8% d'écarts au protocole. En intention de traiter, les taux de FPN étaient de 8% et 9.8% dans les groupes MQ et SP (-1.8% [-4.8, 1.1]), établissant l'équivalence entre les deux traitements. Ce résultat a été confirmé en analyse per protocole. La méfloquine a été plus efficace que la SP pour prévenir les infections placentaires (1.7% vs. 4%, P=0.005), et l'anémie maternelle (Hb<10 g/dL) (16% vs. 20%, P=0.09) de façon marginalement significative. Davantage d'effets indésirables ont été déclarés sous MQ (78% vs. 32%, P<10-3), essentiellement des
vomissements, nausées et vertiges, de courte durée. Un seul épisode psychiatrique a été notifié (sous MQ). Ainsi, cet essai a montré que la méfloquine était une option intéressante
pour le TPI. Il a conduit à la mise en place d'un nouvel essai évaluant la MQ dans cinq pays d'ASS. En parallèle, d'autres molécules et associations médicamenteuses, ainsi que d'autres stratégies de prévention que le TPI ont été proposées et sont actuellement évalués. Leurs avantages et inconvénients seront discutés en comparaison à ceux de la méfloquine.

SII_O2. Evaluation du TPI chez la femme enceinte – Mise en œuvre sur le terrain et impact sur le poids de naissance depuis 2005.

T. d'Almeida, G. Cottrell, M. A. Agboton-Zoumenou ; Y. Imorou, A. Garcia & A. Massougbodji.

Mots clés : paludisme; TPI; SP; femme enceinte; poids de naissance
Adresse : niaren@yahoo.fr

Dans le cadre d'un partenariat REFS/IRD/PNLP, une étude d'évaluation du Traitement Préventif Intermittent (TPI) du paludisme de la femme enceinte à la Sulfadoxine Pyriméthamine (SP) institué au Bénin depuis 2006 a été réalisée. Le TPI consiste à administrer au moins 2 doses d'un antipaludique donné (ici la SP) à partir du deuxième trimestre de la grossesse avec un intervalle d'au moins un mois entre les prises chez les femmes enceintes, malades ou non. Cette étude visait à :
- évaluer la bonne mise en œuvre du TPI (degré de couverture, nombre de doses reçues pendant la grossesse) ;
- mesurer l'impact du TPI à la SP (comparaison entre la période avant et après TPI) depuis son démarrage jusqu'en décembre 2009 sur un indicateur de santé publique qu'est le poids de naissance ;
- évaluer le niveau actuel de connaissance des femmes enceintes ;
- évaluer les difficultés rencontrées dans l'application sur le terrain.

Quatre sites ont été identifiés pour ce travail, répartis au sud et au nord dont 2 urbains et 2 ruraux : Cotonou et Tori Bossito pour le Sud, Malanville et Guéné pour le Nord, à raison de 2 maternités par site soit un total de 8 maternités. La taille de l'échantillon (tiré au sort dans les registres des maternités) concernant surtout la partie rétrospective est de 2500 femmes pour les cinq années de l'étude. L'étude a comporté un versant rétrospectif constitué par le recueil D'informations liées au suivi de la grossesse et à l'accouchement fournies par les registres de consultations prénatales et d'accouchement, et un versant transversal qui a consisté en l'interrogatoire de plusieurs dizaines de femmes enceintes et du personnel des maternités de l'étude. Les données ont été recueillies sur le terrain de Janvier à Juin 2010 et
sont à ce jour en cours d'analyse statistique.

SII_O3. Cohorte de Tori-Bossito : caractérisation moléculaire des clones plasmodiaux infectant la mère et l'enfant.

C. Dechavanne, E. Renard, A. Le Port, G. Cottrell, A. Garcia, A. Massougbodji & F. Migot-Nabias

Mots clés : paludisme *Plasmodium falciparum* ; grossesse; nouveau-né; tolérance immunitaire
Adresse : celia_dv@yahoo.fr

Quatre études menées au Cameroun, en Tanzanie, au Gabon et au Kenya ont montré que les enfants issus de mères infectées par *Plasmodium falciparum* à l'accouchement seraient plus susceptibles aux infections palustres pendant leur enfance que les enfants issus de mères non impaludées. Ces études ont conclu à une hypothèse de tolérance immunitaire de ces nouveau-nés qui serait induite par l'exposition du fœtus à des antigènes palustres solubles. Les nouveau-nés ne développeraient alors pas de réponse immunitaire efficace contre les antigènes parasitaires qu'ils ont rencontrés in utero. Notre étude explore cette hypothèse au niveau moléculaire parasitaire. La comparaison des gènes codant pour des antigènes de stades sanguins des parasites infectant la mère à l'accouchement et l'enfant lors de ses premières infections plasmodiales pourrait apporter quelques réponses. Parmi une cohorte de 647 nouveau-nés résidant au Bénin, zone de méso-endémie palustre, 66 (10.2%) sont issus de mères impaludées à l'accouchement. Des échantillons de sang périphérique de la mère et de sang placentaire ont été récoltés sur papier filtre à l'accouchement. Des échantillons de sang périphérique d'enfants ont été réalisés, selon le même mode, mensuellement de 0 à 18 mois (pour détecter les cas de portages asymptomatiques) ainsi qu'à chaque évènement fébrile (pour détecter les cas de portages symptomatiques). L'ADN est extrait à partir des confettis avec la méthode du Chelex®. Les gènes msp2 et glurp de *P. falciparum* sont caractérisés par analyse de fragments. L'analyse statistique tiendra compte des mesures environnementales (taux d'inoculation entomologique, température, hygrométrie) relevées régulièrement. L'existence d'associations entre les clones infectant la mère à l'accouchement et leurs enfants sera évaluée et permettra de valider l'hypothèse de tolérance immunitaire émise par les épidémiologistes. Cette approche de caractérisation moléculaire apportera l'information sur l'hypothèse de tolérance immunitaire dont la connaissance est primordiale pour l'orientation de la stratégie de contrôle du paludisme.

SII_O4. Survenue des premières infections palustres au Bénin : rôle de l'infection placentaire et de facteurs environnementaux

A Le Port, S. Ouedraogo, G. Cottrell, C. Dechavanne, A. Bouraima, A. Massougbodji, B. Fayomi, F. Migot-Nabias, A. Garcia & M. Cot

Mots clés : paludisme; prévention; placenta; nouveau-né; moustiquaire
Adresse : agnesleport@yahoo.fr

Etudier l'association entre l'infection placentaire pendant la grossesse et la survenue des premières infections palustres pendant les 12 premiers mois de vie, à Tori Bossito, une zone rurale du Bénin. Méthode : 550 enfants suivis une fois par semaine de la naissance jusqu'à l'âge de 12 mois ont été inclus dans l'analyse. Les covariables dépendantes du temps « saison » (pluvieuse/sèche) et « anophèles » (nombre d'anophèles collectés pendant la capture de moustiques la plus proche de la 1ère infection palustre, au maximum toutes les 3 semaines, codé en 3 classes : absence d'anophèles, nombre d'anophèles moyen, nombre d'anophèles élevé), ont été inclus dans deux modèles de Cox, selon si la mère déclarait posséder une moustiquaire ou non (hypothèse des risques proportionnels non respectée). Résultats : Parmi les enfants dormant sous moustiquaire, ceux nés de placenta infecté avaient un HR=2.19 [1.28-3.76], p<10-2 de présenter une première infection palustre plus tôt que ceux nés de placenta non infecté. La covariable dépendante du temps était associée au risque de faire une infection palustre avec un HR=3.50, [2.13-5.74], p<10-4) si le niveau d'anophèles était moyen et un HR=6.10 [2.96-12.56], p<10-4) si le nombre d'anophèles était élevé, comparé à l'absence d'anophèles. La saison a également un effet sur la survenue de la première infection palustre avec un HR= 2.09 [0.99-1.37], p=0.05). Parmi les enfants ne dormant pas sous moustiquaire, il n'y avait pas d'association entre l'infection placentaire et la survenue des premières infections (p=0.64), bien que la saison et l'exposition aux anophèles soient à la limite de la significativité. En conclusion, les enfants nés de placenta infecté sont deux fois plus à risque de présenter leur première infection palustre plus tôt pendant leur 12 premiers mois de vie que les enfants nés de placenta non infecté. Cet effet est visible

uniquement chez les enfants dormant sous moustiquaire, l'effet étant masqué par une plus grande exposition aux anophèles chez les enfants ne dormant pas sous moustiquaire.

SII_O5. Conséquence du paludisme gestationnel sur les réponses cytokiniques à des ligands des TLRS chez les nouveau-nés.

K. Gbédandé, S. Ezinmegnon, S. Ibitokou, C. Agbowai, A. Massougbodji, S. Varani, M. Troye-Blomberg, A. J.F. Luty, P. Deloron & N.Fievet

Mots clés : TLR; CPA; cytokines; *P. falciparum*; nouveau-nés
Adresse : venubiens@yahoo.fr

Le paludisme associé à la grossesse constitue un problème de santé publique. Chez la femme enceinte et pendant la petite enfance, il existe une susceptibilité accrue aux maladies infectieuses due à une immuno-modulation qui inhibe et contrôle les réponses pro-inflammatoires. La mise en place d'une réponse immune protectrice résulte de la reconnaissance, de la présentation de l'antigène et de l'activation des cellules immunitaires via les TLR (Toll-Like Receptors) présents à la surface des CPA (cellules présentatrices d'antigènes). Ces TLR activés induisent la sécrétion de cytokines et l'expression des molécules de costimulation pour réguler la présentation de l'antigène aux lymphocytes T. Chez les nouveau-nés, les CPA via la stimulation des TLRs sont impliqués dans la mise en place progressive des réponses inflammatoires pendant les premiers mois de vie et notre hypothèse est que le paludisme gestationnel altérerait ce développement. L'étude est une partie du projet STOPPAM réalisée à Comé sur une cohorte de 200 femmes enceintes et leurs enfants suivis de la naissance jusqu'à 12 mois. Pour apprécier l'impact de l'infection palustre sur l'immunité néonatale, des données cliniques et parasitologiques (parasitémie et anémie) sont collectées chez la mère pendant la grossesse et chez l'enfant. Le sang total des enfants (0, 3, 6 et 12 mois) est stimulé par 4 ligands des TLRs (polyI.C, LPS, Résiquimod, CpGODN) spécifique de différentes CPA (monocytes, cellules dendritiques myéloïdes et plasmacytoides) et aussi avec les globules rouges parasités ou non par *P. falciparum*. Les cytokines pro-inflammatoires (IL-6, IFN, TNF) et anti-inflammatoire (IL-10) sont dosées dans les surnageants de culture des enfants par cytométrie en flux afin d'étudier un des mécanismes de régulation de la présentation des antigènes chez le nouveau-né. Nous avons réalisé les stimulations et les dosages des cytokines sur 100 échantillons du sang cordon, 75 enfants de 3 mois, 52 enfants de 6 mois et 45 enfants de 12 mois. Les analyses statistiques sont en cours et les résultats seront présentés.

SII_O6. Antibodies that inhibit binding of *Plasmodium falciparum* infected erythrocytes to CSA correlate with improved pregnancy outcomes.

J. Doritchamou, F. Viwami, L. Deneoud, A. Moussiliou, S. Gnidehou, A. Massougbodji, A. Salanti, P. Deloron & N. T. Ndam

Key words : Pregnancy; malaria; vaccine; antibody VAR2CSA
Address : jdorichamou@yahoo.fr

Pregnant women are infected by *P. falciparum* parasites presenting with unique adhesion properties that allow them to specifically bind Chondroitin sulphate a proteoglycan in the placenta. Acquisition of protective immunity over successive pregnancies is attributed to
antibodies that block the adhesion of infected erythrocytes to CSA. In this study we analysed plasma samples of women of various parity enrolled from their first trimester of pregnancy till delivery in the ongoing STOPPAM project in Benin. The plasma level of anti-VSA antibodies and adhesion inhibitory activity were measured on two parasite lines selected

for CSA binding on Bewo cells (FCR3 and HB3). Specific antibodies to var2csa were measured on recombinant proteins of the DBL5 domain and the full-length extracellular part of the VAR2CSA. The majority of primigravidae had low levels or no anti-adhesion
antibodies at enrollment compared to multigravidae. Women who experienced a detected parasitemia during the follow up significantly increased their levels of anti-VSA, anti-var2csa as well as their plasma binding-inhibitory activity, between enrolment and delivery. However a difference in the kinetics of antibody production was observed between primigravidae and multigravidae following an infection. Women infected with HIV displayed an antibody acquisition pattern similar to that of HIV negative primigravidae.
Overall a significant correlation was found between the plasma level of anti-VSA and anti-var2csa IgG and the plasma anti-adhesion activity. High levels of such antibodies correlated with improved pregnancy outcomes in our study population. The results from this study suggest that the anti-adhesion antibodies play a significant role in the protective immunity acquired against pregnancy malaria and should be considered a priority in strategies aiming at developing a vaccine against this pathology.

SII_O7. Prévention du paludisme chez la femme enceinte infectée par le VIH au Bénin : ESSAI PACOME.

L. Denoeud, C. Fourcade, J. Akakpo, F. Atadokpede, A. Afangnihoun, L. Dossou-Gbete RX. Perrin, PM Girard, M.Cot & DM. Zannou

Mots clés : prévention; paludisme gestationnel; VIH; cotrimoxazole; méfloquine
Adresse : camillefourcade@hotmail.com

Le paludisme gestationnel (PG) est une cause de faible poids de naissance (FPN), responsable de mortalité infantile. Chez la femme séropositive pour le VIH, plus vulnérable, l'OMS distingue deux stratégies préventives possibles : soit une prophylaxie quotidienne par cotrimoxazole si elle doit la recevoir pour son VIH ; soit un traitement préventif intermittent (TPI) renforcé en trois prises. L'efficacité du cotrimoxazole n'a jamais été évaluée en prévention du PG. Cet essai randomisé a pour objectif d'évaluer l'efficacité du cotrimoxazole en prévention du PG chez la femme séropositive, comparativement au TPI renforcé par méfloquine. Méthodes : Les femmes sont recrutées à Cotonou et Porto-Novo au
deuxième trimestre de grossesse. Celles éligibles au cotrimoxazole sont tirées au sort pour recevoir soit le cotrimoxazole seul, soit l'association cotrimoxazole et TPI. Les femmes non éligibles reçoivent soit le cotrimoxazole seul, soit le TPI seul. Toutes reçoivent une moustiquaire imprégnée. Le critère de jugement principal est la prévalence de l'infection palustre placentaire. Les stratégies de prévention de la transmission mère-enfant du VIH sont appliquées. Le suivi s'achève aux 4 mois de vie de l'enfant. Résultats préliminaires : Au 15 juin 2010 (6 mois de recrutement), 90 femmes étaient incluses, 43 sous TPI. L'âge moyen était de 29 ans, 11% étaient primigestes. En médiane, la durée du VIH était de 19 mois, le taux de CD4 de 290/mm3. Sur 30 enfants, 24% présentaient un FPN. Aucune apposition placentaire n'était impaludée. Cinq paludismes sont survenus, deux prévalent, et trois pendant le suivi (taux d'incidence : 20/100PA). Une femme est décédée du SIDA et trois avortements se sont produits. La moitié des prises de méfloquine ont été suivies d'effets secondaires, essentiellement des vomissements et des vertiges résolutifs sous 48h. Sous cotrimoxazole, sont survenues deux anémies sévères, mais aucun rash. En conclusion, l'incidence palustre mesurée est comparable à celle retrouvée chez des adultes séropositifs sous cotrimoxazole au Kenya. Les effets secondaires à la méfloquine ne diffèrent pas de ceux observés chez la femme séronégative, ils sont fréquents mais bénins. L'efficacité des deux stratégies sera mesurée ultérieurement, le recrutement se poursuit pour atteindre un effectif de 500 femmes.

SII_O8. Etudes pangénomiques de susceptibilité au paludisme : discordances et conséquences.
A. Garcia, J. Milet & A. Sabbagh

Mots clés : Paludisme; Etudes pangénomiques; Cartographie génétique; Polymorphismes
Adresse : Andre.Garcia@ird.fr

Les progrès récents dans les domaines de la génétique moléculaire et de la statistique ont permis la multiplication des études pan génomique (ou génome entier) de susceptibilité aux maladies multifactorielles. Dans le domaine du paludisme 4 études ont, à des degrés divers, utilisé ces approches pour différents phénotypes, accès palustre simple, accès sévère, anémie ou portage asymptomatique. Trois d'entre elles ont utilisé des puces de types Affimétrix® de 10 000, 250 000 et 500 000 polymorphismes simples de l'ADN (SNP), la quatrième utilisant une carte de 400 microsatellites. Les résultats de ces études sont intéressants mais discordants. Seule la région q31-q33 du chromosome 5 et la région 12q22 sont retrouvée par deux de ces études. Pour expliquer ces discordances plusieurs hypothèses sont proposées : hétérogénéité génétique, complexité des phénotypes étudiés et de la prise en charge des facteurs de confusion éventuels, prise en compte des tests multiples ? Une étude très récente met en avant et démontre clairement l'existence d'une autre hypothèse selon laquelle les outils de génotypage actuellement disponibles pour ces études pangénomiques ne seraient pas adaptés aux populations africaines. En effet, les puces à ADN utilisées sont générées par le projet HapMap basé sur des SNPs initialement découverts sur des populations d'origine européenne. Dans les populations africaines les taux de déséquilibre de liaison sont beaucoup plus faibles. Si elle se révélait exacte, cette hypothèse pourrait avoir de très lourdes conséquences sur les résultats passés et sur les études à venir. Je propose de présenter les principaux résultats de ces différentes études, leurs discordances et les différentes hypothèses classiques explicatives. Enfin je terminerai en exposant les problèmes posés par les outils de génotypage et les différentes possibilités de prise en compte.

SII_O9. Stratégies d'Elimination du paludisme basées sur la dynamique saisonnière de transmission dans les pays sahéliens de la CEDAO: "transmission targeted malaria elimination strategies" in ECOWACS région
K. D. Ogobara

Mots clés. Paludisme; saisonnalité; sahel; régionale; CDEAO.
Adresse *:* okd@icermali.org

La lute contre le paludisme connait actuellement un regain d'intérêt local, national et international avec de nombreuses initiatives (RBM, PMI, GMP, etc.…). Depuis son lancement à Seatle par Bill & Melinda Gates, de plus en plus de voix et d'initiatives, évoquent la possibilité nouvelle d'Elimination/Eradication du Paludisme. La restitution du programme MalEra en mars 2010 aux partenaires de recherche et de lutte contre le paludisme à Washington a permit de proposer des axes de recherche innovants pour accompagner l'initiative de l'Elimination/Eradication du paludisme. Les continents, les pays endémiques s'organisent sur le plan régional pour dégager des stratégies adaptées. Les pays de la SADEC ont mis en place des structures régionales et des moyens conséquents de financements et de ressources humaines et prédisent d'éliminer la transmission autochtone du paludisme dans les états membres d'ici 2020-2025. Les pays de la CEDEAO pourraient s'organiser aussi en tenant compte du caractère saisonnier de la transmission et imaginer des stratégies innovantes d'élimination sous-régionale du paludisme. Les traitements intermittents préventives: IPTp, IPTi ont été recommandés par l'OMS et s'intègrent harmonieusement dans les stratégies nationales. Nous avons

testé dans le cadre d'un consortium ouest africain, financé par la Bill and Melinda Gates Fondation, en collaboration avec la London School of Tropical Médical Hygiène; une nouvelle stratégie que nous avons nommée IPTc, base sur le caractère saisonnier de la transmission dans les pays du Sahel. Les résultats obtenus au Mali depuis son initiation en 2003-2004, au Sénégal, au Burkina Faso et en Gambie; montrent une efficacité de 60-80% sur les indicateurs majeurs malariamétriques sur déjà une saison de transmission. Ces évidences scientifiques ont été présentés à l'OMS Genève en juillet 2010 afin d'engager un processus de "guideline" stratégique. La prise en compte de cette particularité saisonnière de la transmission dans les pays sahéliens peut permettre de développer une stratégie régionale innovante et adaptée d'élimination du paludisme dans la région sahélienne Ouest Africaine.

SII_O10. L'infection par *Schistosoma haematobium* affecte les réponses IgG anti-*Plasmodium falciparum* associées à la protection palustre

A. D. Saïah, J. Milet, V. Soulard, O. Gaye, F. Migot-Nabias, R. Sauerwein, A. Garcia, A. J.F. Luty & D. Courtin.

Notre étude menée dans la région de Niakhar au Sénégal a montré que les individus présentant des réponses élevées d'anticorps dirigées contre les antigènes candidats vaccins MSP-1, MSP-2 et GLURP avaient un risque diminué de développer un accès palustre ou de fortes parasitémies. Dans la présente étude, nous avons évalué l'influence possible de l'infection par *Schistosoma haematobium*, fréquente dans cette région, sur les réponses IgG anti-Plasmodium falciparum précédemment associées à la protection palustre. Les résultats obtenus montrent des niveaux d'IgG1 dirigés contre MSP-1 et GLURP plus faibles chez les individus porteurs d'helminthes. Ces derniers présentent également des niveaux d'IL-10 plus élevés que les individus non infectés par *Schistosoma haematobium*. Cette modulation des réponses immunes et en particulier des IgG1 spécifiquement dirigées contre *Plasmodium falciparum* chez les porteurs d'helminthes pourrait donc augmenter le risque d'infection ou de maladie palustre.

Posters

SII_P1. L'importance des Clubs de santé dans la gestion des conséquences du Paludisme chez la femme enceinte et le nouveau né dans la perspective de développement d'un vaccin pour la femme enceinte.

A. Damien, K. Ida & K. Paulin

Mots clés : Légumes et fruits traditionnels; nutrition; santé; recherche; communication
Adresse : apretectra@yahoo.fr

Promotion de la santé à partir de la consommation accrue des fruits et légumes traditionnels. Recherche sur la production et la consommation et soutien à la vulgarisation; disponibilité de semences, d'informations et de formations, partages avec d'autres pays: Kenya, Tanzanie, Togo et Sri Lanka

SII_P2. Données manquantes et censure dans l'analyse de données longitudinales : application aux données immunologiques de Tori Bossito.

D. Thiam

Mots clés : Données longitudinales; censures; modèles mixtes

Adresse : djenaba.thiam@gmail.com

En zone endémique, l'enfant non immun, est la principale cible du paludisme. Les anticorps maternels persistent dans le sang circulant 6 mois environs ; protégeant ainsi l'enfant durant cette période pendant laquelle il commence à développer sa propre immunité. Le suivi de la
cohorte de 600 nourrissons mises en place à Tori Bossito permettra une meilleure compréhension de la cinétique de l'acquisition de la réponse immune grâce à des mesures répétées des taux d'Ac spécifiques de *P. falciparum*. Cette étude intègrera des facteurs de risques environnementaux et comportementaux (infection placentaire, risque environnemental) Cependant les données longitudinales issues d'étude de cohorte sont soumises à de nombreux types de problèmes tels que les données manquantes (décès, abandon) ou les données non observées (dues à des problèmes techniques). D'autre part, il est important aussi prendre en compte la variabilité liée à chaque individu de la population étudiée. Il existe divers outils statistiques pour gérer ces types de problèmes et ainsi optimiser les résultats. Nous avons utilisé les modèles linéaires à effet mixtes pour analyser les données immunologiques. Il s'agit de modèles adaptés aux données répétées et permettant de prendre en compte la variabilité liée à chaque individu. Ce type de données présente 2 types de censures. Une censure à gauche correspond au cas où les taux d'anticorps sont en dessous du seuil de détection et une censure à droite au cas inverse (taux au dessus d'un certain seuil). Une approche classique consiste à remplacer ces censures par certaines valeurs (ex, la moitié du seuil de détection pour les censures à gauche). Cependant ces méthodes sont connues pour introduire du biais dans l'estimation des paramètres, ce qui a été vérifié par des simulations. Nous avons implémenté un algorithme d'imputation multiple de type Stochastique Expectation Maximisation (SEM) afin de gérer les censures dans notre modèle. Ces deux méthodes sont comparées. Une fois la prise en compte des censures optimisée, la prochaine étape sera d'inclure dans la modélisation les variables environnementales et comportementales et d'utiliser des modèles de types classes latentes pour étudier l'hétérogénéité de la cinétique d'acquisition de la réponse immune.

SII_P3. Conséquences du paludisme pendant la grossesse : apport de l'échographie au sein d'une cohorte au Bénin.

B.T.Huynh, N. Fievet, G. Gbaguidi, A. Massougbodji, P. Deloron & M. Cot

Mots clés : paludisme; grossesse; cohorte; âge gestationnel; petit poids de naissance
Adresse : bichtrambe@hotmail.com

En zone d'endémie, les conséquences du paludisme gestationnel sont l'anémie maternelle et le petit poids de naissance (PPN) du nouveau-né (<2500g). En Afrique Sub-saharienne, les femmes se rendent tardivement en consultation prénatale (CPN) et l'âge gestationnel (AG) n'est pas précisément mesuré. Ainsi, l'influence de la période de survenue des infections palustres en termes de conséquences sont mal connus. De plus, les mécanismes de constitution du PPN (prématurité ou retard de croissance intra-utérin (RCIU)) sont difficiles à distinguer. Une cohorte prospective de femmes enceintes a été constituée au Bénin. Durant son suivi, une femme se rend en moyenne à 4 CPN et peut consulter en urgence. Quatre échographies sont effectuées pour déterminer l'AG et surveiller la croissance fatale. A chaque CPN et chaque visite d'urgence, un examen gynécologique, des prélèvements sanguins et un test de diagnostic rapide du paludisme (TDR) sont faits. Chaque femme reçoit un traitement préventif intermittent et une moustiquaire. Actuellement, 1036 femmes ont été incluses et 990 ont terminé leur suivi. Le dernier accouchement est prévu pour Aout 2010. Vingt-trois pour cent des femmes ont été incluses durant leur premier trimestre de grossesse. Durant le suivi, nous avons relevé 11% de TDR positifs se répartissant ainsi: 9.3% durant le premier trimestre, 65.2% durant le deuxième et 25.5% durant le troisième. Au moment de l'accouchement, sur les 853 femmes ayant donné naissance dans le cadre du projet, 9.3% ont eu un TDR positif. Sur les 845 nouveau-nés pesés, 13.1% ont un PPN et parmi eux 45.9% sont nés prématurément (avant 37 SA). Ce travail permettra d'identifier les moments-clés qui ont le plus de conséquences pour la mère et son nouveau-né et de connaitre la part attribué à la

prématurité ou au RCIU devant un PPN. Ces résultats pourraient changer les actions de santé publique en encourageant les femmes à venir consulter à des moments privilégiés et appuyer les approches de lutte intégrée afin de protéger la femme durant toute sa grossesse.

SII_P4. Decreased *in vitro* sensitivity to dihydroartemisinin.

A.Aubouy, A. Wakpo, J.F. Faucher, S. Houze, S. Ezinmegnon, A. Massougbodji, P. Deloron & J. Le Bras

Key words : *falciparum*, traitement, artémisinine
Address: agnes.aubouy@ird.fr

Artemisinine derivative-based combination therapies (ACT) have been adopted by many *P. falciparum* malaria endemic countries as first-line treatment of uncomplicated malaria. The inefficiency of chloroquine and sulfadoxine-pyrimethamine in most areas, and the efficiency and safety of such combinations prompted the World Health Organization (WHO) to recommend in 2006 the use of ACT in all malaria endemic areas for uncomplicated malaria. Efficiency of ACT seems to maintain a high level in Africa and Asia, although in vivo failures are not rare during monitored clinical assays, frequently around 5%. A greater loss of efficiency would raise a serious problem for malaria control. So that it is of high importance to report and study resistance cases. Here we report three cases of decreased in vitro sensitivity to dihydroartemisinin after therapeutic failure with ACT. Materials and methods the efficacies of arthemeter-lumefantrine and artesunate-amodiaquine for the treatment of uncomplicated malaria were compared during an unsupervised therapeutic assay described elsewhere. *P. falciparum* isolates were collected from children < 5 years old between May and November 2007 in southern-Benin. Sampling was performed both at inclusion and during in vivo follow-up in case of treatment failure. Parasitized blood samples obtained in vivo were washed and cultured in vitro in drug-coated plates for 42 hours before freezing. The in vitro assays were revealed by pLDH ELISA production with the commercial kit ELISA-Malaria antigen test (DiaMed AG, Cressier s/Morat, Switzerland). Our results show an immediate decreased in vitro sensitivity to DHA of the infecting isolates. Data on multiplicity of infection, blood drug levels, and fifty percent inhibitory concentrations (IC50) have been taken into account to discuss the results. In conclusion, artemisinine resistant *P. falciparum* clones could be selected in the years to come in African endemic areas. Alternative treatments need to be evaluated, and efficacy surveillance should be actively performed in endemic falciparum areas.

SII_P5. Evaluation d'une stratégie de traitement du paludisme du nourrisson limité aux cas confirmés par TDR.

J. Bouscaillou & A.Garcia

Mots clés : Paludisme; Test de diagnostic rapide; Nourrisson; Stratégie de traitement
Adresse : julie.bouscaillou@gmail.com

Afin d'éviter l'émergence de résistances, l'abandon du traitement présomptif des accès palustres non compliqués, pour un traitement à base d'artémisinine limité aux cas confirmés par TDR est maintenant une recommandation de l'OMS et tend à entrer dans les protocoles nationaux des pays concernés par une forte transmission de paludisme. Il persiste néanmoins quelques réticences liées à un manque d'évaluation de cette stratégie et des performances des TDR chez les enfants de moins de 5 ans, principales victimes des décès liés au paludisme. Cette stratégie de traitement des seuls cas confirmés par TDR a été utilisée durant les 18 premiers mois de vie d'une cohorte de 600 nourrissons à Tori Bossito, Bénin. Six cents nourrissons ont été suivis au cours de leurs 18 premiers mois, dans l'objectif initial d'étudier les déterminants génétiques, biologiques et

environnementaux de la survenue des premiers accès palustres. Au cours de ce suivi, toutes les consultations au dispensaire ont été enregistrées. Les nourrissons consultant pour fièvre ont bénéficié systématiquement d'un TDR ainsi que d'une goutte épaisse et n'ont reçu un traitement à base d'artémisinine qu'en cas de TDR positif. Les nourrissons étaient invités à reconsulter en cas de non amélioration et bénéficiaient en plus d'une visite hebdomadaire par les relais communautaires. Les performances du TDR utilisé, ainsi que le devenir des faux négatifs au TDR ont pu être évalués. Au cours de ce suivi, 2142 consultations pour fièvre ont été enregistrées, toutes ont bénéficié d'un TDR et d'une goutte épaisse. Le test de diagnostic rapide parascreen a montré en comparaison à la goutte épaisse une sensibilité de 95.5% et une spécificité de 89.4%. Au total, 28 nourrissons ont eu un TDR faussement négatif et n'ont initialement pas été traités. Aucun d'entre eux n'est décédé ou n'a été victime de complications nécessitant une référence à l'hôpital. En conclusion, les résultats suggèrent que l'utilisation de TDR au paludisme pour décider d'un traitement à base d'artémisinine peut être performante chez le nourrisson.

SII_P6. Relation entre densité anophélienne et facteurs environnementaux à Tori Bossito (Bénin).

B. Kouwaye, G. Cottrell, C. Pierrat, A. Bouraima, A. Massougbodji, N.Fonton, N. Houkonnou & A. Garcia

Mots clés : Paludisme; Densité anophélienne; Modèles mixtes; Facteurs environnementaux
Adresse : kouwaye2000@yahoo.fr

L'hétérogénéité de l'espace de vie des jeunes enfants occasionne des variations au niveau de la densité anophélienne, l'anophèle étant le principal vecteur du paludisme. L'étude de l'exposition au vecteur dans les lieux où vivent les enfants est indispensable pour comprendre la dynamique et de l'expression clinique des premières infections. Pour comprendre les facteurs intervenant dans la survenue des premières infections palustres chez le nouveau-né, il a été constitué une cohorte de 600 nourrissons suivis de la naissance jusqu'à l'âge de 18 mois. Cette étude a été réalisée à Tori-Bossito zone rurale du sud Benin. L'objectif de notre travail est de déterminer les facteurs environnementaux pouvant expliquer variabilité de la densité anophélienne dans la zone d'étude au niveau du village et à une plus petite échelle au niveau de la maison. Pour caractériser l'exposition au vecteur à la quelle sont soumis les nourrissons, 19 missions de capture de moustiques ont été réalisées dans les 9 villages à raison de 4 maisons par village. Pendant toute la période du programme, différentes variables ont été mesurées : climatiques (pluviométrie, saison?), caractéristiques du milieu (l'indice de végétation, type de sol, présence de cours d'eau...) caractéristiques de l'habitat (type de toit, nombre d'ouvertures) comportements des habitants (nombre de personnes dormant dans une chambre, possession de moustiquaire) Le modèle statistique que nous avons mis en œuvre est un modèle mixte de Poisson à 3 niveaux (capture, maison, village) avec interceptes aléatoires au niveau village et au niveau maison, où le nombre d'anophèles capturés par maison et par mission est expliqué par les variables environnementales. Les facteurs environnementaux significativement associés à un risque d'exposition ont été : la saison, la pluviométrie mais aussi des facteurs caractérisant l'environnement immédiat de la maison : le type de sol, la présence de cours d'eau, l'indice de végétation. Nous avons ainsi montré l'importance de l'environnement immédiat dans le risque d'exposition au vecteur auquel sont soumis les jeunes enfants. Ce modèle permettra d'affecter un risque spatio-temporel d'exposition au vecteur à tous les enfants du programme qui sera pris en compte dans l'analyse des déterminants des premières infections chez le nouveau-né.

SII_P7. Caractéristiques individuelles de la réponse anticorps anti palustre chez la femme enceinte, et conséquences pour le nouveau né

F.Migot-Nabias, C. Dechavanne, E. Guitard, A. Bouraima, D. Courtin, A. Le Port, G. Cottrell, A. Massougbodji, J. M. Dugoujon & A. Garcia

Mots clés : Paludisme; *Plasmodium falciparum*; Immunoglobulines; Allotypes Gm et Km; Nouveau-né
Adresse : florence.migot-nabias@ird.fr

Les allotypes Gm et Km sont des déterminants antigéniques polymorphes des immunoglobulines (IgG) conduisant à 18 allotypes Gm possibles au niveau des chaînes lourdes gamma-1, -2 et -3 constitutives des iso types IgG1, IgG2 et IgG3, et à 3 allotypes Km possibles pour les chaînes légères kappa. La combinaison pour une même immunoglobuline de plusieurs allotypes définit des phénotypes Gm et Km, qui peuvent être associés à des particularités de la réponse immunitaire. La pression de sélection opérée par les agents infectieux contribue à maintenir le polymorphisme génétique de ces déterminants,
mais très peu d'études ont envisagé leur rôle dans le cas des maladies infectieuses parasitaires. Dans une précédente étude menée au Bénin, nous avons conclu à l'association d'un phénotype Gm particulier à la protection contre l'accès palustre non compliqué à *Plasmodium falciparum* chez l'enfant. La présence de ce phénotype était corrélée positivement au taux d'IgG1 et d'IgG3 dirigées contre un antigène de *P. falciparum* associé à la physiopathologie du paludisme. Dans la présente étude, nous recherchons si les allotypes Gm et Km des immunoglobulines contribuent de par leur diversité au contrôle génétique de la réponse anticorps spécifique du paludisme chez la femme enceinte. Si certains déterminants sont associés à une production accrue d'anticorps anti palustres liés à la protection clinique, ils pourraient être impliqués dans une diminution de la pression parasitaire exercée in utero par les antigènes parasitaires solubles. Il s'ensuivrait une plus faible sensibilisation immunitaire du fœtus, qui serait accompagnée d'une apparition plus tardive des premières manifestations cliniques du paludisme chez le jeune enfant. Au Bénin, dans une zone endémique pour le paludisme, 647 femmes ont été recrutées au moment de l'accouchement, 66 d'entre elles (10,2%) présentant une infection palustre placentaire. Leurs IgG plasmatiques totales, IgG1 et IgG3 dirigées contre 7 antigènes de *P. falciparum* candidats à l'inclusion dans un vaccin polyvalent contre le paludisme, ont été déterminés. Les relations chez la mère entre les allotypes Gm et Km des immunoglobulines et les taux d'anticorps spécifiques sont analysées, ainsi que leurs répercussions sur les nouveau-nés, suivis de 0 à 18 mois sur les plans clinique, parasitologiques et immunologique.

SII_P8. Impact des polymorphismes maternels de gènes de cytokines sur les paramètres biologiques et immunologiques de la mère et du fœtus dans le cas du paludisme placentaire.
L. G. Adjimon, C. Dechavanne, A. Bouraima, D. Courtin, A. Le Port, R. Ladekpo, J. Noukpo, D. Bonou, C. Ahouangninou, F. Loumedjinon, M. Daoudou, A. Garcia & F. Migot-Nabias

Mots clés : Paludisme; grossesse; cytokines; polymorphisme; anticorps
Adresse : gbenalog@yahoo.fr

Certaines mutations ponctuelles de gènes de cytokines sont associées à des modifications de la production des cytokines correspondantes, avec des répercussions sur la réponse anticorps. Nous avons étudié les conséquences de polymorphismes de gènes de cytokines sur le niveau d'anticorps spécifiques de la mère à l'accouchement et du fœtus, en présence ou non d'une infection palustre placentaire. Nous pensons que si des polymorphismes de gènes maternels de cytokines associés à une production accrue d'anticorps anti palustres liés à la protection clinique sont identifiés, ils pourraient être impliqués dans une diminution de la pression parasitaire exercée in utero par les antigènes parasitaires solubles. Il s'ensuivrait une plus faible sensibilisation immunitaire du fœtus, qui serait accompagnée d'une apparition plus tardive des premières manifestations cliniques du paludisme chez le jeune enfant. Notre étude porte sur 576 paires d'échantillons de mères et d'enfants provenant du sud-ouest du Bénin, où le

paludisme est endémique. A l'accouchement, le sang périphérique des mères a été prélevé, ainsi que le sang du cordon correspondant. Onze pour cent des mères avaient un
placenta infecté par *Plasmodium falciparum.* A partir de l'ADN génomique maternel, 5 mutations ponctuelles dans les gènes codant pour l'IL-4, l'IL-10 et l'IL-13 ont été déterminées par PCR quantitative. Les génotypes suivants étaient très représentés : IL-4 -590 TT (62, %), IL-4 +33 CT (50%), IL-10 -1082 AA (53%), IL-10 -592 AC (51%) et IL-13 -1055 CT (51%). Nous avons évalué l'influence de ces polymorphismes de gènes de cytokines 1) sur la capacité des cellules mononucléées maternelles et fatales à produire les cytokines d'intérêt, suite à des stimulations mitogéniques et 2) sur les taux d'IgM et d'IgG plasmatiques maternelles et fatales dirigées contre des antigènes de *P. falciparum* candidats à
l'inclusion dans un vaccin polyvalent contre le paludisme. L'analyse des relations entre les polymorphismes de gènes de cytokines maternelles, les productions des cytokines maternelles correspondantes, et la production d'anticorps spécifiques chez la mère et le fœtus, contribuera à la compréhension des interactions immunologiques entre la mère et l'enfant pendant la grossesse, en présence ou non d'une infection palustre placentaire.

SII_P9. Paludisme associé à la grossesse : caractérisation des cellules présentatrices d'antigènes chez la femme enceinte.

S. Ibitokou, N. Fievet, B. Vianou, C. Agbowai, S. Ezinmegnon, M. Oesterholt, A. Massougbodji, T.Theander, S. Varani, M. Troye-Blomberg, A. J.F. Luty & P. Deloron

Mots clés : *Plasmodium falciparum*; grossesse; cellules présentatrices d'antigènes
Adresse : ibitokou_samad@yahoo.fr

Le paludisme associé à la grossesse (PAG) est un problème de santé publique. Caractérisé par l'accumulation des hématies parasitées dans le placenta, il est responsable d'anémie et d'avortements maternels. Les cellules présentatrices d'antigènes (CPA), interviennent dans
L'initiation et la régulation des réponses immunitaires. Les cellules dendritiques myéloïdes et plasmacytoides (mDC et pDC) constituent des cellules professionnelles dans la présentation d'antigènes et interviennent dans la régulation des réponses inflammatoires pendant la grossesse. STOPPAM est un suivi de cohorte de 1000 femmes enceintes qui se déroule en Tanzanie et au Bénin. Des données cliniques et immunologiques sont collectées afin de quantifier les effets du PAG et proposer un candidat vaccin. Nous avons inclus 2 groupes de 150 et 106 femmes, respectivement en début de grossesse et à l'accouchement pour les études d'immunité cellulaire. Nous avons estimé les fréquences et mesuré les niveaux d'activation des CPA à travers la moyenne d'intensité de fluorescence (MIF) de l'expression des molécules d'activation (HLA-DR) et de costimulation (CD86). En début de grossesse, les femmes infectées par *P. falciparum* présentent une diminution significative d'expression de HLA-DR sur les CPA et une augmentation de CD86 sur les pDC et lymphocytes B par rapport aux femmes non infectées : HLA-DR mDC (649, 3 vs 1113,9) ; HLA-DR pDC (143,3 vs 316,2) ; HLA-DR Monocytes (216,7 vs 273,8) ; CD86 pDC (8,5 vs 6,73) et CD86 Lymphocytes B (10,0 vs 5,83). A L'accouchement, les fréquences de mDC et de pDC des femmes infectées sont significativement réduites par rapport aux femmes non infectées (%mDC : 92,44 vs 94,94 et %pDC : 69,74 vs 71,45). De façon générale, le PAG modifie soit les fréquences des CPA, soit leur niveau d'activation chez la femme enceinte. L'incapacité des CPA à exprimer de manière concomitante le HLA-DR et le CD86 pendant le PAG pourrait induire l'altération des réponses immunologiques de la femme enceinte. La faible fréquence de CPA déjà décrite pendant la grossesse serait accentuée au cours du PAG. Les CPA seraient alors mobilisées par les organes profonds comme le placenta pour augmenter l'efficacité des réponses.

SII_P10. Etude de la réponse IgG anti *Plasmodium falciparum* au niveau qualitatif et quantitatif chez des jeunes enfants béninois.

A. Djilali-Saïah, C. Deschavane, A. Bouraima, Firmine XXX, G. Cottrell, A. Le Port, F. Migot-Nabias, A. Garcia & D. Courtin

Mots clés : GIA; RGIA; *P. falciparum*; vaccin; jeunes enfants.
Adresse : dsaek82@yahoo.fr

De nombreuses études séro-épidémiologiques ont étudié la relation entre les niveaux de réponses IgG et la protection palustre. Certaines études ont montré une association entre des réponses élevées d'IgG spécifiquement dirigées contre les antigènes AMA1, MSP1, MSP2, MSP3 et GLURP et la protection palustre, d'autres n'ont pas retrouvé l'association entre les niveaux d' IgG et la protection palustre. Les discordances observées pourraient être liées à l'absence de prise en compte de la fonctionnalité des IgG. Notre étude a pour objectif d'étudier le rôle quantitatif et qualitatif des réponses IgG de jeunes enfants dans la protection contre le paludisme. L'étude a été menée dans la région de Tori Bossito au Bénin. Un suivi parasitologique et clinique a été réalisé sur 550 enfants de la naissance à 18 mois. Ce suivi a permis de définir différents groupes d'individus en fonction de leur phénotype clinique (nombre d'accès palustre). Le prélèvement de plasma réalisé en fin de suivi (18 mois) a permis de quantifier les niveaux d'IgG spécifiquement dirigés contre les antigènes MSP1, MSP2, MSP3, AMA1 et GLURP et d'isoler des IgG pour estimer leur rôle fonctionnel, in vitro, en utilisant des tests d'inhibition de la croissance parasitaire. Les IgG issues des enfants ayant développé un seul accès palustre au cours du suivi ont une capacité d'inhibition de la croissance parasitaire plus élevée ($p=0{,}0009$; Kruskal-Wallis) que ceux provenant d'enfants ayant eu plusieurs accès palustres pendant le suivi. Ces derniers ont pourtant des niveaux d'IgG anti-MSP1, MSP2, MSP3, AMA1 et GLURP plus élevés. Les IgG dirigées contre AMA1 représentent une forte proportion (55 %) de l'inhibition de la croissance parasitaire (souche FCR3) observée en utilisant les IgG totaux. En conclusion, la majorité des études séro-épidémiologiques ont étudié l'association entre des quantités d'anticorps et la protection palustre. Nos résultats montrent que la qualité (fonctionnalité) des IgG est également un facteur important à prendre en compte, au même titre que la quantité, dans les études séro-épidémiologique. L'importance du rôle fonctionnel joué par IgG anti-AMA1 montre que cet antigène est un bon candidat vaccin.

SII_P11. Etiology of anemia during pregnancy and consequences on the infant in a malaria endemic area.

S. Ouedraogo, M. Accrombessi, A. Massougbodji & M. Cot

Key words : Anemia; Pregnancy; Etiology; Consequences
Address: accrombessimanfred@yahoo.fr

More than 50% of the pregnant women are affected by anemia in developing countries, the main cause being iron deficiency. Our first hypothesis is that iron deficiency represents over 50% of the causes of anemia in Beninese pregnant women. Anemia in pregnancy has consequences on the child. Our second hypothesis is that anemia in pregnancy is associated with anemia in the first months of life. Rational anemia during pregnancy is an important issue in developing countries where 5-10% and more than 50% of pregnant women develop severe and moderate anemia, respectively. Severe anemia seems to increase mortality and morbidity in mothers, but little is known on its risk factors and especially on its impact on both fetuses and infants. Malaria and iron deficiency are important well-known risk factors for anemia during pregnancy. Pregnancy is associated to a significant increase in the amount of iron required to increase the red cell mass, expand the plasma volume and to allow for the growth of the fetal placental unit; in Africa the need in iron is not covered. Other risk factors include helminthiases, genetic disorders, micronutrients deficiency and undernutrition. Early anemia has been associated with an increase of preterm delivery. Allen suggested three potential mechanisms whereby maternal iron

deficiency anemia might give rise to preterm delivery: hypoxia, oxidative stress, and infection. For placental malaria, another important contributor to maternal anemia in sub-Saharan Africa, immunological factors are thought to play a role, particularly placental TNF release and monocyte infiltrates. Available studies, even showing a link between anemia during pregnancy and preterm and low birth weight babies, have seldom taken into account risk factors for anemia like infections which might have led to these outcomes and none, to our knowledge, has studied the long-term consequences on the children. Our main objective is to determine the links between anemia in pregnancy and anemia in infants of six months of age, in a malaria endemic area. Secondarily, we will estimate the different types of anemia during pregnancy, focusing on iron deficiency anemia. During this meeting we will present the preliminary analysis which deals with the etiology of anemia in pregnancy.

SII_P12. Spontaneous postpartum clearance of *Plasmodium falciparum* parasitaemia in Beninese women.

J. Bottero, V. Briand, C. Agbowai, J. Doritchamou, A. Massougbodji & M. Cot

Key words : Pregnancy; Malaria; Postpartum; Africa
Address: valerie.briand@gmail.com

The effects and consequences of gestational malaria are well documented, still little is known about malaria in the immediate postpartum. A single study published in the 1980s demonstrated that women who were parasitaemic at delivery cleared their parasitaemia spontaneously within 48 hours postpartum. To confirm this phenomenon we investigated the spontaneous early postpartum evolution of malaria infections at delivery in Beninese women. Methods Women were part of a large clinical trial which aimed to compare the efficacy of sulfadoxine-pyrimethamine and mefloquine for intermittent preventive treatment. Women who were infected with *Plasmodium falciparum* at delivery had a control of their parasitaemia in the early postpartum, as soon as the infection was detected. No antimalarial drugs were given unless women were symptomatic. Giemsa-stained thick blood smears were used to estimate parasite densities. Smears were recorded as negative if no parasite was detected after the examination of 200 microscope fields. 1601 women were recruited for the trial. Of them, 1346 (84%) had a peripheral thick blood smear at delivery. Thirty five (2.5%) women were infected with *P. falciparum* at delivery. Parasitological follow-up could not be interpreted in 15 of them as they were treated for symptoms, 18 cleared parasitaemia spontaneously within 5 days postpartum, and had a strong decrease of parasitaemia before getting treated. In conclusion, all women infected with *P. falciparum* at delivery who did not receive an antimalarial drug cleared their parasitaemia spontaneously within 5 days after delivery. This result supports the idea that, being a privileged site for the sequestration and multiplication of parasites, the placenta facilitates the persistence of parasitaemia during pregnancy, and its elimination induces a rapid clearance of parasites postpartum.

SII_P13. Conséquence du paludisme gestationnel sur les réponses immunologiques du nouveau-né : étude des lymphocytes T régulateurs.

O. Nouatin, C. Agbowai, S. Ibitokou, S. Ezinmegnon, N.Fievet, A. Massougbodji, S.Varani, M.Troye-Blomberg, A. J.F. Luty & P. Deloron

Mots clés : *Plasmodium falciparum*; Treg; nouveau-né
Adresse : nouatinodilon@yahoo.fr

Caractériser les facteurs associés à une plus grande susceptibilité des nouveau-nés à l'infection à *P. falciparum* est une priorité pour la validation d'un vaccin. Chez les nouveau-nés, la réponse pro-inflammatoire est en partie contrôlée par les lymphocytes T régulateurs (Treg). Très importants pour le maintien de l'homéostasie, le rôle principal des Treg est de réprimer l'activité des cellules de

l'immunité. Les Treg sont caractérisés par le phénotype : CD4+ CD25+ CD127- Foxp3. Ils représentent 2 à 10% des lymphocytes. Leur prévalence est plus importante dans les échantillons de sang de cordon issus d'un placenta parasité, entrainant des conséquences sur l'efficacité de la réponse immune. Ces résultats suggèrent une stimulation in utéro par des antigènes solubles de *P. falciparum* et une altération des réponses pro-inflammatoires. Le projet STOPPAM réalisé dans la zone sanitaire de Comé, effectue un suivi longitudinal de 1000 femmes enceintes parmi lesquelles 200 femmes et leurs nouveau-nés seront pris en compte dans le cadre de cette étude. Des données cliniques et parasitologiques sont collectées au cours de la grossesse et chez l'enfant afin de définir les conséquences de l'infection palustre pendant la grossesse et au cours de la première année de vie. Notre objectif est de confirmer les résultats observés sur les Treg à la naissance et de les compléter en suivant l'évolution des prévalences des Treg au cours de la première année de vie. Les marquages sont faits à partir du sang total avec des anticorps monoclonaux anti-CD4, anti-CD25, anti-CD127, anti-Foxp3 et l'acquisition se fait par cryométrie en flux Nous avons réalisé les marquages sur des échantillons provenant de 200 cordons, 200 enfants de 3 et 6 mois, et de 120 enfants de 12 mois. Les résultats en cours d'analyse seront présentés.

SII_P14. Epidémiologie moléculaire de Plasmodium spp dans une population d'enfants d'âge scolaire du sud Bénin

A.Moussiliou, J. Doritchamou, A. Aubouy, A. Massougbodji, J-F. Faucher & N.TuikueNdam

Mots clés : Plasmodium; diagnosti;, qPCR; Bénin
Adresse : azizathmoussiliou@yahoo.fr

Le paludisme demeure la parasitose la plus importante et touche particulièrement les enfants et les femmes enceintes. En Afrique, il tue un enfant toutes les trente secondes et représente 18% de cas d'hospitalisation au Bénin. Des approches existent sur le plan vaccinal et des progrès ont été réalisés sur le plan thérapeutique avec l'élaboration de molécules nouvelles pour contourner des résistances. Cependant l'élimination de cette parasitose dans des régions endémique comme l'Afrique Sub-saharienne ne sera possible sans la maîtrise de la mortalité et de la morbidité associées. Ainsi des améliorations sont-elles indispensables dans le diagnostic pour assurer un meilleur suivi et une meilleure évaluation de l'ensemble des efforts de lutte. Peu de données sont disponibles sur la prévalence des infections à Plasmodium sp au Bénin. Le besoin d'une technique de diagnostic très sensible et efficace, devient indispensable pour faire un état des lieux des différentes espèces qui circulent dans la région. Dans cette étude nous avons développé une technique de PCR en temps réel multiplex pour évaluer la prévalence des différentes espèces de Plasmodium dans une cohorte d'enfants du Sud Bénin. Sur des échantillons sanguins provenant d'une cohorte de 552 enfants, nous avons réalisé un diagnostic de Plasmodium sp par microscopie, nested-PCR et comparé à la détection par PCR quantitative en temps réel (qPCR). La meilleure sensibilité des techniques PCR est confirmée, avec un seuil de quantification à 8 copies de gène. La présentation détaillée des résultats et leur interprétation feront l'objet de cette communication.

SII_P15. Risque palustre : quels déterminants environnementaux de la transmission ? Etude chez des nouveau-nés en milieu rural, Bénin.

C. Pierrat, A. Bouraima, G. Cottrell, A. Le Port, C. Dechavanne, A. Massougbodji, S. Ouedraogo, A. Garcia & M. Tabeaud

Mots clés : Paludisme; risque environnemental; SIG
Adresse : charlotte.pierrat@gmail.com

L'une des spécificités des pays d'Afrique Sub-saharienne comme le Bénin est leur climat subtropical, chaud et humide. Ces caractéristiques climatiques permettent l'existence d'une transmission endémique du paludisme. Cependant, à très fine échelle, les gens ne sont pas tous exposés de la même manière à ce risque sanitaire. Il est ainsi nécessaire de comprendre quelles caractéristiques de l'environnement sont déterminantes à micro-échelle, par la conduite d'un diagnostic territorial. Il a été mené dans une zone rurale du sud du Bénin composée de neuf villages, Tori Bossito. Le but a été de spatialiser le risque de transmission, en identifiant d'une part les zones où le vecteur *Anophèles gambiae* peut se reproduire, et d'autre part quels individus de la cohorte (600 nouveau-nés) sont exposés à ce vecteur. Le diagnostic territorial comprend une collecte d'indicateurs environnementaux multi-échelles (région, village, maison), et une analyse de géographie humaine sur les pratiques d'utilisation des moyens de protection (moustiquaires, insecticides), la structure de l'habitat, l'existence de champs cultivés, zones de travaux à proximité des maisons étudiées. L'enjeu était de mieux comprendre à la fois la variabilité temporelle du risque (comment l'alternance de saisons sèches et humides influence le vecteur), et sa variabilité spatiale (rôle des conditions sociales et de l'occupation du sol). La modélisation de ces variables dans un SIG (Système d'Information Géographique), précédée d'un travail de terrain et d'enquêtes, a permis de mieux comprendre les déterminants de la transmission palustre dans une approche systémique à fine échelle. L'originalité de cette étude tient à la prise en compte non seulement de l'aléa climatique (qui explique la variabilité temporelle du risque), mais aussi et surtout les conditions sociales qui entraînent la vulnérabilité des individus au paludisme. Cette méthode pourrait s'avérer intéressante pour la lutte antipaludique, par la prise de conscience de l'importance des modes de vie dans le risque palustre.

SII_P16. Prévention du paludisme pendant la grossesse : évaluation de l'effet du Traitement préventif intermittent distribué au Bénin

A. Le Port, G. Cottrell, C. Dechavanne, V. Briand, A. Bouraima, A. Massougbodji, B. Fayomi, F.Migot-Nabias, A. Garcia & M. Cot

Mots clés : paludisme; prévention; grossesse
Adresse : michel.cot@ird.fr

Les moyens de prévention du paludisme pendant la grossesse ont été comparés dans trois études faites au Bénin, en se basant sur les taux d'infection placentaire et de faibles poids de naissance. La première étude a été faite au moment où la prophylaxie par chloroquine était encore recommandée (étude préclinique), la seconde était un essai clinique sur le Traitement Préventif Intermittent pendant la grossesse (TPIg) comparant la Sulfadoxine Pyriméthamine (SP) à la méfloquine et la 3ème étude était une étude observationnelle après la mise en place du TPIg national par SP. Nous avons montré un impact du TPIg sur la réduction du FPN (10% avec le TPIg national et 8.7% dans l'essai clinique, vs 15.7% dans l'étude préclinique). L'effet sur l'infection placentaire était meilleure dans l'essai clinique (2.9% vs 11.2% et 16.7%, pour le TPIg national et l'étude préclinique). En dépit d'une bonne adhérence générale au TPIg national (avec 84% de femmes prenant au moins une dose de SP), des écarts au schéma des prises supervisées persistent. Ces écarts pourraient être atténués par une meilleure formation des agents de santé.

SII_P17. Etude ethnobotanique et ethno pharmacologique des plantes vendues sur les marchés au sud Togo contre le paludisme.

K. Koudouvo, K. Kokou, K. Essien, D. Karou, K. Aklikokou, A.I. Glitho, J. Simpore, R. Sanogo, & M. Gbeassor

Mots clés : Ethnobotanique; Plantes antipaludiques; herboristes;Togo
Adresse : kkoudouvo@gmail.com/koudouvo@tg.refer.org

Les études menées au Togo sur l'activité antiplasmodiale des extraits de plantes sur *Plasmodium falciparum in vitro*, l'ont été sans enquêtes ethnobotaniques préalables. Or à côté du recours aux pratiques courantes de la médecine traditionnelle ou au répertoire des herbiers nationaux pour le choix des plantes pour des études pharmacologiques et phytochimiques, les enquêtes ethnobotaniques demeurent le moyen le plus fiable. C'est pour cette raison que la présente étude à été menée au près des herboristes (vendeurs de plantes médicinales) des marchés du sud Togo pour recenser les plantes vendues à leurs clients pour soigner le paludisme. La méthode utilisée est l'"achat en triplet" ou "méthode KKAGGSS" en cours d'élaboration au Togo, couplée avec la méthode classique d'interview semi-structuré. A l'issue des travaux, 137 espèces appartenant à 118 genres et 53 familles ont été recensées. Les familles les plus représentées sont les Caesalpiniaceae, les Euphorbiacées et les Rubiaceae. Ces plantes sont des arbres, herbes, arbustes et lianes vivant dans les savanes, les forêts, dans les champs de culture ou plantées dans des jardins domestiques. Seize (16) plantes ont été citées pour la première fois comme espèces antipaludiques. Les feuilles, les racines, l'écorce et la tige feuillée sont les parties de ces plantes les plus utilisées en médecine traditionnelle togolaise. Les recettes de plantes sont préparées essentiellement par décoction et par macération. La voie orale (79, 28%) est la principale voie d'administration. Une méthode sélective a permis de retenir 14 plantes pour des tests pharmacologiques d'évaluation leurs propriétés antiplasmodiales sur *P. falciparum* in vitro. Des tests préliminaires sur trois d'entre elles (*Opilia amanthacea*, *Pavetta corymbosa* et *Tamarindus indica*) ont donné des résultats significatifs pouvant permettre de confirmer leurs utilisation en médecine traditionnelle togolaise contre le paludisme.

SII_P18. Evaluation de la chimio sensibilité *in vitro* de *Plasmodium falciparum* à la monodéséthylamodiaquine, la quinine.

A.Wakpo, S. Ezinmegnon, S. Houze, JF. Faucher, A. Massougbodji, P. Deloron, J. Le Bras & A. Aubouy

Mots clés : Mots clés : *Plasmodium falciparum*; antipaludiques; efficacité *in vitro*
Adresse : wakpoabel@yahoo.fr

La pharmaco résistance de *Plasmodium falciparum* contribue à l'augmentation de la morbidité et de la mortalité palustre dans les pays endémiques. Dans le cadre de la surveillance de l'efficacité des antipaludiques au Bénin, cette étude avait pour objectif principal d'évaluer la sensibilité *in vitro* de *P. falciparum* à la monodéséthylamodiaquine, la quinine, la méfloquine, la dihydroartémisinine, la luméfantrine et à la pyriméthamine. Les isolats de *P. falciparum* ont été obtenus dans la zone sanitaire d'Allada, à l'occasion d'une étude *in vivo* d'efficacité thérapeutique chez l'enfant. Les niveaux de sensibilité des isolats aux 6 antipaludiques ont été déterminés grâce à la méthode d'immuno détection de la Plasmodium lactate déshydrogénase : EMAT (ELISA malaria antigène test). L'intérêt de la méthode pour la surveillance des niveaux de chimiosensibilité de *P. falciparum* a été discuté et les résultats obtenus *in vitro* ont pu être confrontés à ceux obtenus *in vivo*. Les isolats testés (n=92) ont montré une absence de résistance *in vitro* à la quinine, la luméfantrine et à la dihydroartémisinine. Par contre, une résistance *in vitro* de *P. falciparum* à la méfloquine, la monodéséthylamodiaquine et à la pyriméthamine dans les proportions respectives de 2,6%, 3,8% et 61,8% a été obtenue. La méthode EMAT a montré une facilité de mise en place mais un faible taux de réussite (57,3%). Les résultats des tests *in vitro* étaient cohérents par rapport à ceux obtenus *in vivo*. Les données obtenues dans cette étude pourraient constituer une base de données pour la surveillance de la chimiorésistance *in vitro* de *P. falciparum* au Bénin.

SII_P19. Evaluation de la faisabilité et de l'acceptabilité de l'utilisation de l'Arteméther-Luméfantrine (Coartem®) dans le traitement à domicile du paludisme à Houéyogbé (Bénin).

R. A. Houngnihin, D. Kindé-Gazard, B. Dénakpo, E. Chaffa, H.Kossou, S.Koudoukpo, D. Gbénou, S. B. Sirima & F. Pagnoni.

Mots clés : acceptabilité; Arteméther; Luméfantrine; traitement; paludisme; Houéyogbé
Adresse : roch_houngnihin2001@yahoo.fr

L'utilisation de l'Arteméther-Luméfantrine (Coartem®) dans le traitement à domicile du paludisme est une stratégie visant à accroitre l'accès des populations à risque faiblement couvertes par les services de santé, aux médicaments de qualité. Cette approche s'inscrit dans le cadre du changement de politique antipaludique et implique des défis liés à la disponibilité, l'accessibilité et l'acceptabilité des médicaments et la capacité des populations à les utiliser. La faisabilité et l'acceptabilité d'une telle approche méritent d'être évaluées. L'étude est de type transversal, descriptif et analytique. Elle a porté sur 785 mères dont les enfants ont eu la fièvre dans les deux semaines précédant l'enquête. L'approche a consisté à former les agents de santé et les relais communautaires et à développer des outils d'éducation à l'endroit des mères d'enfant qui sont capables d'utiliser l'Arteméther-Luméfantrine mis en place dans tous les villages. L'évaluation de la faisabilité et de l'acceptabilité intervenue six mois après, a été réalisée au niveau des 30 villages retenus par un tirage aléatoire simple. Les résultats montrent que 97,8% des mères d'enfants de moins de 5 ans ont une bonne connaissance du paludisme identifiée par la fièvre/corps chaud. L'automédication est le premier recours, ce qui n'empêche pas 92,5% des mères de recourir au « médicament du Blanc ». Parmi celles qui ont utilisé le « médicament du Blanc », 52,8% ont eu recours au « médicament pré-emballé de l'étude » (le Coartem®). Environ 70,9% des mères ont obtenu le traitement au niveau du village ; et l'accès aux soins intervient dans une proportion d'environ 75% dans les 24 heures après l'apparition des premiers symptômes. Aussi, 89,1% des mères ont respecté le traitement de la fièvre avec les CTA pendant trois jours, ce qui augure d'une bonne perspective lorsqu'on s'inscrit dans une mise à l'échelle. En conclusion, malgré sa grande rapidité d'action pouvant amener les mères à arrêter le traitement devant une amélioration de l'état clinique de l'enfant, l'Arteméther-Luméfantrine est pris systématiquement pendant toute la durée du traitement (respect de la durée et de la dose). Son utilisation est donc faisable et acceptée par les communautés.

SII_P20. Genetic Diversity of *Plasmodium falciparum* Merozoite Surface Protein-1 block 2 (MSP-1) in sites of contrasting altitudes and malaria endemicities in the Mount Cameroon region.

O. Kengne. J. Arnaud , S. Wanji, E. E. J. Eyong, H. K. Kimbi, C. Charvet, J. L. Ndamukong-Nyanga, N. D. Hugues, C. Bourguinat & N. Tendongfor

Key-words: Genetic Diversity; *Plasmodium falciparum*; MSP-1; malaria endemicities; Mount Cameroon region.
Address : arnaudkengne@yahoo.com

Knowledge of the molecular epidemiology of parasite in an area is essential in understanding the transmission of a disease such as malaria. The present study was performed to appreciate the genetic diversity of *P. falciparum* circulating in sites of contrasting altitudes with different malaria endemicities in the Mount Cameroon region. Methods Blood samples were collected from asymptomatic children from the six localities classified into 3 groups (high, intermediate and low altitudes). Microscopy was used to screen for *P. falciparum* positive samples, to determine the parasite load and the prevalence of the infection in each locality. Landing catch mosquito collection was done and CSP-ELISA performed to determine the entomological indices. Using a PCR-based technique and MSP-1 block 2 as genetic marker, 142 randomly selected *P. falciparum* positive blood samples were genotyped. This was followed by Sequencing to confirm genotyping results for the K-1 allelic variants. The highest malaria prevalence (70.65%) and EIR (2.15 inf. b/n) were recorded at low altitude while the lowest prevalence

(12.33%) and EIR (0 inf. b/n) were obtained at high altitude. Globally, all the allelic variants K-1 (18.75%), MAD20 (13.04%) and RO33 (20%) were less represented at high altitude compared to low altitudes which had the highest frequency for K-1 (46.87%). The highest frequencies for MAD 20 (52.17%) and RO33 (48%) were observed at intermediate altitude. 48 (54.02%) samples had multiclonal infections. There was a trend of increasing mean number of clones per sample with a decrease in altitude. Spearman correlation test showed no significant association between complexity of infection, malaria prevalence, parasite density, and EIR. Sequencing confirmed PCR-results of the allelic variant K-1 and revealed a new K-1 allelic variant. In conclusion, the work showed a positive association between low altitude where transmission is high and MSP-1 block 2 polymorphism as well as complexity of infection. The uneven allelic polymorphisms observed in this work may be a hindrance for malaria vaccine formulations if not considered in vaccine development studies.

Resumes / Abstracts SESSION 3

Approche innovantes de lutte et de gestion des résistances chez les vecteurs du paludisme

Innovative strategies for controlling and managing insecticide resistant malaria vectors

Conférences scientifiques / Scientific conferences

SIII_C1. Place de la lutte anti vectorielle dans le projet d'élimination du paludisme

M. C. Akogbeto

Mots clés : Paludisme; *Anophèles gambiae*; résistance.

Adresse : akogbetom@yahoo.fr

Malgré les nombreux efforts déployés par les états africains pour lutter contre le paludisme, les résultats obtenus dans la dernière décennie sont en deçà de ceux escomptés. Toutefois, la communauté internationale ne baisse pas les bras. Mieux, depuis quelques années, les efforts de lutte sont renforcés en vue de réduire l'incidence de cette affection de manière qu'elle ne soit plus un problème de santé publique. Certes, le programme d'éradication du paludisme des années 50 n'a pas donné des résultats satisfaisants dans les pays africains, mais aujourd'hui, le contexte a changé. En effet, la volonté politique se manifeste de plus en plus aux plus hauts sommets des états en faveur de la lutte contre le paludisme. Par ailleurs, les partenaires financiers investissent de plus en plus dans la recherche et la lutte contre le paludisme. De plus, de nouveaux outils de lutte sont disponibles (MILD, PID, CTA, TDR, TPI). Quel est l'apport de la lutte anti-vectorielle dans cet arsenal? Nous n'avons pas la prétention de comparer ces différentes stratégies entre elles, mais de parcourir les progrès accomplis dans le domaine de la lutte anti-vectorielle depuis la période du programme d'éradication du paludisme. Dans la décennie 1990-2000, l'introduction de moustiquaires imprégnées a entrainé une réduction de la morbidité palustre allant de 40% à 63% chez les enfants au Kenya, en Tanzanie, en Guinée Bissau et en Gambie et 5,5 vies sauvées sur 1000 en Gambie, au Kenya, au Ghana et au Burkina (Len geler et al.

1997). Dans le projet Mékong, une protection massive de plus de 10 millions d'habitants contre les piqûres de moustiques par une combinaison de moustiquaires imprégnées et de pulvérisation intra domiciliaire a réduit, sur une période de 10 ans, la mortalité due au paludisme de 5000 à 0 décès. Selon W. P. O Méhara *et al.* (2010) (Lancet Infectious Diseases, 10 : 8, pp 545-555, 2010), le poids du paludisme est en nette régression en Afrique au sud du Sahara grâce à une combinaison de plusieurs mesures de lutte. L'article indique que dans l'Ile de Bioko en Guinée Equatoriale, l'utilisation simultanée de la PID, des MILD et des CTA a conduit, en 4 ans (de 2003 à 2007), à une réduction de 90% de la positivité des anophèles en antigène circumsporozoïtique de *Plasmodium falciparum*. Dans la même période, la parasité palustre chez les enfants de moins de 5 ans a chuté de 42 à 18% et la mortalité dans le même groupe d'âge a été réduite de 70%. L'utilisation des moustiquaires imprégnées et la pulvérisation intra domiciliaire d'insecticide sont un espoir pour la lutte contre le paludisme. Mais, la lutte anti vectorielle ne peut pas seule conduire à l'élimination du paludisme. Elle doit être associée aux autres stratégies de lutte (CTA, TDR, TPI). En effet, même si certaines stratégies de lutte anti vectorielle comme la PID peuvent induire une réduction de 98% de la capacité vectorielle des anophèles, le taux de reproduction restera supérieure à un (donc pas de diminution de la prévalence de l'infection) dans un contexte de forte agressivité anophelienne (ma = 100). Or, si la PID est la seule méthode capable d'agir efficacement sur le taux de survie des anophèles pour réduire de façon significative la capacité vectorielle, sa mise en œuvre n'est pas à la portée des communautés. Cette mise en œuvre nécessite des compétences techniques car, elle repose sur 4 questions. Quels produits appliquer? Où les appliquer? Quand les appliquer? Comment les appliquer?

SIII_C2. Prospects for use of fungus-based biopesticides in novel strategies of integrated vector management

M. B. Thomas, S. Blanford & A. F. Read

Key words: Fungal entomopathogen; *Beauveria bassiana*; sustainable malaria control; evolution of resistance; Integrated Vector Management.
Address : mbt1965@gmail.com; mbt13@psu.edu

In the last few years there have been a number studies exploring the potential for using entomopathogenic fungi as novel biopesticide interventions against adult malaria mosquito vectors. The research has been motivated by the fact that the effectiveness and sustainability of current insecticide-based strategies to control malaria is being increasingly threatened by the evolution of insecticide resistant mosquitoes. Cross-resistance between the limited number of chemical insecticide classes approved for public health use adds to the problem further. Fungal entomopathogens act via contact so potentially lend themselves to strategies currently used for delivery of chemical insecticides such as indoor residual sprays, treatment of mosquito resting targets and impregnation of materials such as curtains. These approaches exploit the natural behavior of many important malaria vectors to feed and rest in and around the domestic environment and hence contact treated surfaces at some point during the gonotrophic (feeding and reproduction) cycle. However, while existing public health insecticides are all essentially fast acting neurotoxins, the biological processes of spore germination, penetration of the insect cuticle and then growth and proliferation within the insect, mean that fungal pathogens take several days to kill the mosquito following infection. Here we present an overview of current empirical and theoretical evidence to demonstrate that relative slow speed of kill does not necessarily matter from a malaria control perspective. In part this is because the relatively long extrinsic incubation period of the malaria parasite within the mosquito creates a window of several days for the fungus to act. Additionally, a number of side-effects of fungal infection mean that the ability of mosquitoes to transmit malaria can be substantially reduced well before death. Furthermore, theoretical work suggests there may even be evolutionary benefits of slower speed of kill since this enables mosquitoes to achieve at least part of their lifetime reproductive output and so reduces the selection pressure for resistance. These results, together with data demonstrating that fungal

pathogens do not suffer cross resistance with chemical insecticides and can even exhibit insecticide resistance-breaking properties, highlight the potential for use of insect fungal pathogens within novel strategies of integrated vector management. More generally they point to the possibility of malaria control using products other than fast-acting insecticides, potentially creating opportunities for a range of new active ingredients to supplement conventional insecticide approaches.

Communications orales / Oral communications

SIII_O1. Situation de la résistance aux pyréthrinoïdes et aux carbamates chez *Anophèles gambiae* dans les sites cotonniers du Bénin en fonction des stratégies de protection appliquées par les producteurs.

A.Yadouléton, F. Chandre, L. Djogbenou, T. Martin & M. Akogbeto

Mots clés : Coton; *Anophèles gambiae*; Insecticides; résistance
Adresse : anges33@yahoo.fr

Les traitements insecticides destinés à la protection de la culture du coton ont été souvent évoqués comme principal facteur de sélection de la résistance *d'Anophèles gambiae*, principal vecteur du paludisme, aux insecticides. Pour vérifier cette hypothèse, nous avons évalué la résistance d'*An. gambiae* s.s dans les zones cotonnières du Bénin, selon trois programmes de protection contre les ravageurs: le programme calendaire avec utilisation de fortes quantités d'insecticides, le programme à lutte étagée ciblée (LEC) avec très peu d'insecticide et le programme biologique où aucun insecticide chimique n'est utilisé. Dans un premier temps, nous avons effectué une enquête sur la nature des pesticides utilisés contre les ravageurs de coton, leur origine et les doses appliquées. Dans un second temps, nous avons étudié la sensibilité des anophèles issus des sites d'étude aux papiers imprégnés de deltaméthrine (0,05 %), de perméthrine (0,75%) de DDT (4%), et du bendiocarb (0,1%) et enfin, nous avons procédé à la caractérisation moléculaire et à l'identification des mécanismes de résistance chez *An. gambiae.* Il ressort des résultats que les planteurs de coton utilisent plusieurs familles d'insecticides (les pyréthrinoides, les organochlorés, les organophosphorés et les carbamates). Dans les sites à traitement biologique, une décortission de feuilles de Neem ou de papayer en combinaison avec le piment et le savon local est utilisée pour traiter les plants de coton. Concernant le statut de résistance des moustiques dans les champs de coton, *An.gambiae* apparaît résistant aux pyréthrinoïdes (75% de mortalité) et au DDT (48%), mais sensibles à la deltaméthrine et au bendiocarb (97%) dans les zones à programme calendaire et à lutte étagée ciblée (respectivement, zones à forte et faible utilisation d'insecticides). Par contre, dans les zones de coton biologique, *An.gambiae* a été trouvé sensible à l'ensemble des insecticides sauf au DDT. Selon les résultats de l'analyse moléculaire (PCR), le complexe *An. gambiae* des zones d'étude est composé d'*An. gambiae* s. s (65%), et *d'An. arabiensis* (35%) et de deux formes moléculaires: S (15%) et M (85%). La fréquence du gène Kdr, principal mécanisme de résistance observé en Afrique de l'Ouest chez *An. gambiae* est de 66,7% dans les moustiques issus des sites à insecticide et 33,3% dans les sites sans insecticide. Cette étude confirme une fois encore que les traitements insecticides sont responsables de la sélection de la résistance aux pyréthrinoïdes chez *An.gambiae* en Afrique de l'Ouest.

SIII_O2. Impact des stratégies de protection du coton sur l'homme et l'environnement: sélection de la résistance des vecteurs du paludisme aux insecticides

M. Namountougou, K.R. Dabiré, F. Simard, T. Baldet, M. Thibaud, A. Diabaté.

Mots-clés: *Anopheles gambiae*, paludisme, lutte antivectorielle, résistance, insecticides, coton.
Adresse : a_diabate@hotmail.com

La lutte anti vectorielle basée sur l'utilisation des moustiquaires imprégnées d'insecticides et/ou de pulvérisations intra domiciliaires est l'une des stratégies majeures de prévention contre le paludisme. Dans sa mise en œuvre à travers les programmes nationaux de lutte contre le paludisme, deux difficultés majeures apparaissent: i) la faisabilité opérationnelle (acceptabilité et utilisation correcte de ces outils) et surtout ii) la résistance des vecteurs aux pyréthrinoides, principaux insecticides utilisés en santé publique. La résistance des vecteurs a été souvent associée à l'utilisation intensive des insecticides en agriculture notamment dans la lutte contre les ravageurs du cotonnier. L'objectif de la présente est d'étudier l'évolution de la résistance d'*An. gambiae* s.l. en fonction des degrés d'utilisation d'insecticides et de l'ancienneté des zones cotonnières au Burkina Faso. Des tests de sensibilité au DDT 4%, à la perméthrine 1%, la deltaméthrine 0,05% et au bendiocarb 1% ont été réalisés en tube OMS avec des spécimens d'*An. gambiae* sl collectées sur 26 sites entre 2008 et 2009. L'identification des espèces, des formes moléculaires M et S et la détection des mutations *kdr* et *ace-1*^{R} ont été effectuées par PCR sur les spécimens vivants et morts issus de l'exposition aux différents insecticides. Les résultats ont montré que les populations d'*An. gambiae* s.l. étaient résistantes à tous les insecticides testés dans tous les sites à l'Ouest du pays tandis qu'elles montraient une résistance intermédiaire à la deltaméthrine et au bendiocarb dans les zones cotonnières les plus récentes. La fréquence allélique du *kdr* a été observée à un niveau très élevé chez la forme S d'*An. gambiae* variant entre 98 à 80 % dans le bassin cotonnier classique. Elle a été observée à un niveau moyen de 40% à l'échelle du pays (avec un maximum de 95% à l'Ouest du pays) alors qu'elle n'avait été observée chez cette forme que dans une seule localité à VK7 à une fréquence de 4% en 2000. Une augmentation de la fréquence du *kdr* chez *An. arabiensis* a été aussi atteignant 30% vs 1 spécimen en 2000 avec une extension dans de nouvelles zones géographiques. La mutation *ace-1*^{R} a été détectée dans les deux formes moléculaires d'*An. gambiae* ss à une fréquence variant entre 60 et 5% seulement dans l'ancienne zone cotonnière avec un taux d'utilisation d'insecticides plus élevé. Le rôle de l'utilisation intensive des pesticides dans la protection du cotonnier comme source de sélection de la résistance des vecteurs est discuté.

SIII_O3. The complexity of DDT-pyrethroid reistance in *Anopheles gambiae* Giles: an investigation of alternative insecticides for use on bednets and the development of long lasting formulations for indoor residual spraying in malaria endemic settings.

R.N'Guessan, P. Boko, A. Odjo , E. Vignonou, H. Adje, A. Asidi, M. Akogbeto & M. Rowland.

Key words: Resistance; pirimifos methyl; Chlorfenapyr; Indoxacarb; experimental huts
Address : raphael.n'guessan@lshtm.ac.uk

A combination of pyrethroid resistance mechanisms, including kdr, metabolic detoxifiers and emerging cell defence mechanisms are increasingly being detected in the malaria parasite vector *Anopheles gambiae*. This is having a serious impact on control of this vector in some
Western parts of Africa. As we move into the malaria eradication era, it is vital that we look into alternatives to replace or supplement pyrethroids for indoor residual spraying and treatment of bednets. The London School of Hygiene & Tropical Medicine with support from the IVCC is evaluating a number of candidates in Benin and some of them have shown promising results worth reporting. Under laboratory and field conditions in experimental huts, two novel insecticides, chlorfenapyr, a pyrrole and indoxacarb, an oxadiazine showed greater control potential of pyrethroid resistant *An. gambiae* and *Culex quinquefasciatus* Say than pyrethroids. Both are slow acting and lack irritancy property, making their operational mode of action more suitable for IRS than for ITN. For use on bednets their deployment will require combination with pyrethroids but longer lasting formulations of these molecules are desirable to sustain vector control. DDT is the most cost effective and long lasting residual insecticide for IRS programmes but has a negative environmental impact.

Two micro encapsulated formulations (CS) of pirimifos methyl, an organophosphate applied as an IRS treatment at 0.5 g/m2 and 1 g/m2 were evaluated as alternatives in experimental huts in southern Benin where *An. gambiae* and *Cx. quinquefasciatus* are resistant to pyrethroids but susceptible to organophosphates. Both CS formulations induced high mortality of wild *An. gambiae* and Cx. *quinquefasciatus* in mud and cement huts, and prolonged residual activity for several months. In mud huts 1 g/m2 was clearly superior to 0.5 g/m2 and induced >50% mortality of *An. gambiae* for more than 10 months. In cement walled huts 0.5 g/m2 induced high levels of control for almost one year. Pirimifos methyl CS was also found to be highly effective against Culex, giving between 90-50% control for up to 10 months at 1 g/m2 on cement surfaces and for 6 months on mud surfaces. The limited environmental persistence and residual activity makes pirimifos methyl CS a more attractive prospect than DDT or pyrethroids for IRS programmes and would enable pyrethroids to be restricted for ITNs and slow down the acceleration of pyrethroid resistance. A cost effective alternative to DDT, giving year-round transmission control in endemic rural African settings is now a realistic prospect.

SIII_O4. Première évaluation de l'efficacité parasitologique de moustiquaires imprégnées et/ou des bâches plastiques imprégnées vs les aspersions intra domiciliaires à Balombo (Angola).

P. Besnard, L. Le Mire, V. Foumane, J-C. Toto, A. I. Da Silva, A.Soyto, M. A. Dos Santos, F. Quinda, F. Fortes & P. Carnevale.

Mots clés : Moustiquaires imprégnées; bâches plastiques imprégnées; aspersions intra domiciliaires; gouttes épaisses; indice d'endémicité
Adresse : prbesnard@yahoo.fr

Depuis 2007 nous menons, dans 8 villages de la région de Balombo (Angola) une étude comparative de l'efficacité des bâches plastiques imprégnées de deltaméthrine dites « Wall lining » (170 mg ma ?/m2) par rapport aux moustiquaires classiques PermaNet® 2.0 (55 mg ma ?/m2) et aux aspersions intra domiciliaires AID (lambdacyhalothrine à la dose ciblée de 25 mg ma/m2) et à l'association «bâches imprégnées » de type « Zéro Fly®» (360.mg ma /m2) + PermaNet®2.0 au dessus des lits. En 2008 nous avons recueilli les données de base et mis en œuvre les opérations de lutte (en décembre 2008) évaluées en 2009. L'évaluation est conceptuellement pluridisciplinaire, entomologique, parasitologique, immunologique, sociologique etc. Nous rapportons ici les résultats parasitologiques obtenus par confection et lectures de gouttes épaisses (# 5000/an) réalisées lors d'enquêtes transversales (6/an) par passages régulièrement répétés sur les échantillons randomisés chaque fois (« cross sectional surveys »). En se concentrant sur l'indice, classique en paludologie, d'endémicité(= prévalence plasmodiale chez les enfants 2- 9ans) en 2009 par rapport à 2008 on remarque:
- Une diminution générale de l'ordre de 53% des indices d'endémicité dans les 8 villages (de 38,9% en 2008 à 18,3% en 2009) ;
- Une diminution significative et comparable dans les 8 villages ;
- Une diminution significative, quelle que soit la méthode LAV employée : avec les moustiquaires imprégnées P2.0 (de 36,2% à 20,1% soit : 44,5%) ; les WL (de 33,3% à 16,2% soit : 51,3%), les AID (de 40,5% à 18,8% soit : 53,6%) et la réduction la plus importante a été obtenue avec l'association ZF + P2.0 (de 45,7% à 17,6% soit : 61,5%)
- Un ratio de risque (RR) entre 2008 et 2009 parfaitement comparable pour les Wall Lining et les aspersions intra domiciliaires (respectivement RR = 2,05 [1,70-2,49] et RR = 2,14 [1,83-2,51] alors que pour les moustiquaires imprégnées seules le RR est de 1,80 [1,53-2,11] et pour l'association moustiquaires imprégnées + Zéro Fly le RR est le plus élevé RR= 2,60 [2,16-3,10].

SIII_O5. Phase III. Evaluation of insecticide treated Durable Lining in Rural African Houses for control of malaria vectors in western Kenya

R. Allan, S. Munga & J. Vulule

Key words: Durable Lining; IRS; LLIN; Resistance; Innovative
Address: richard@mentor-initiative.net

Conventional, insecticide treated bed nets (ITNs) and now long lasting impregnated nets (LLINs) and IRS have become the primary tool for national malaria control programs in Kenya and elsewhere in Sub-Saharan Africa. However, achieving high net coverage and adherence to net use remains a major challenge. IRS is effective, but short lasting (3-9 months) and therefore difficult to sustain. Material durability if LLINs is also now proving to be much shorter than first expected. Parasite resistance to the many of the insecticides currently used in IRS and LLINs further threatens our ability to achieve and sustain malaria control/elimination. Thus there is an urgent need for alternative and innovative long lasting tools and insecticides for community based malaria prevention. In this study, we assessed the efficacy of insecticide treated Durable Lining (DL) in reducing malaria transmission and DL acceptability in 10 paired rural villages in western Kenya. Five villages were used as control and the remaining five were used as treatment. We installed treated and untreated durable wall linings in the sleeping rooms of the treatment and control houses respectively. The indoor resting densities of mosquitoes were monitored at regular time points in 10 randomly selected houses from each village. Further, we monitored malaria prevalence in young children from control and intervention villages respectively. Overall, indoor resting densities of *An. gambiae* s.l. and An. funestus in villages with DL were respectively 70% ($p = 0.02$) and 60% ($p = 0.005$) lower compared to the control villages over the study period. Malaria prevalence was significantly lower ($P<0.001$) amongst households with DL than households with control linings. Similarly, lower aneamia levels were recorded in DL villages compared to the control villages. Further, a significantly higher number of the households 95% (475/500) perceived DL to be beneficial in controlling malaria vectors. In conclusion these results confirm the acceptability and efficacy of DL as a effective and suitable malaria prevention tool. DL provides and alternative to IRS and may be used in combination with, or as an alternative to LLINs depending on the context.

SIII_O6. Evaluation épidémiologique de la lutte anti vectorielle contre le paludisme.

C. Rogier & M-C. Henry

Mots clés : Paludisme; évaluation; lutte antivectorielle
Adresse : christophe/rogier@wanadoo.fr

La mise au point des moustiquaires imprégnées d'insecticide à longue durée d'action et le renouveau d'intérêt pour les aspersions intra domiciliaires d'insecticides, en association avec les combinaisons thérapeutiques comprenant un dérivé de l'artémisinine, permettent de contrôler le paludisme et d'envisager son élimination d'une proportion de plus en plus importante des zones d'endémie. Au delà du caractère réaliste ou irréaliste de l'objectif d'élimination dans la plupart des zones d'endémie, la lutte contre les anophèles lance un défi à la communauté scientifique et aux programmes nationaux de lutte contre le paludisme, celui de l'évaluation de ces interventions…et de l'utilisation optimale des fonds importants engagés pour ces moyens de lutte. Quelles qu'elles soient, la mise en oeuvre, l'adaptation et l'évaluation des stratégies de lutte anti vectorielle font appel à des compétences paludologiques en entomologie et en épidémiologie. L'évaluation épidémiologique de la lutte anti vectorielle contre le paludisme fait appel à une méthodologie moins bien connue et moins utilisée que l'évaluation entomologique. Elle est pourtant indispensable pour évaluer l'impact de la lutte anti vectorielle sur la santé des populations. Cette lutte concerne l'ensemble des moustiques qui piquent une communauté humaine. L'ensemble de cette communauté peut donc bénéficier, directement ou indirectement, de l'intervention. Les évaluations doivent prendre en compte cette dimension communautaire et reposer sur des indicateurs paludométriques mesurés chez l'Homme.

SIII_O7. Evaluation communautaire de stratégies de lutte anti vectorielle contre le paludisme combinant deux traitements insecticides : étude pré intervention.

B.G.Damien, A. Djènontin, C. Rogier, V. Corbel, F. Chandre, M. Akogbéto & M-C. Henry

Mots clés : paludisme; transmission; maladie; infection; moustiquaires imprégnées.
Adresse : barikiss2000@yahoo.fr

En prélude à l'évaluation de l'impact sur le paludisme de plusieurs stratégies de Lutte Anti Vectorielle (LAV) associant des moustiquaires imprégnées de deltaméthrine à longue durée d'action et de bendiocarb en aspersion intra domiciliaire ou en imprégnation de bâches murales, une étude épidémiologique du paludisme a été réalisée dans le sud Bénin. Cette étude a démarré juste après la distribution de masse de Moustiquaires Imprégnées d'Insecticide à Longue Durée d'action (MILD) aux enfants de moins de 5 ans et aux femmes enceintes. Elle a été réalisée dans 28 villages de la zone sanitaire de Ouidah – Kpomassè -Tori Bossito (sud-Bénin) d'octobre 2007 à mai 2008. Du point de vue entomologique, la dynamique de la population anophélienne, son taux d'infection sporozoïtique et le niveau de résistance lié au gène *kdr* ont été déterminés. Du point de vue parasitologique et clinique, une surveillance longitudinale de la maladie par détection active des cas et une étude transversale de l'infection ont été menées chez les enfants de 0-5 ans. Un suivi de la couverture et de l'utilisation des MILD a été effectué. L'analyse des données entomologiques, parasitologiques et cliniques a été effectuée. Elle a permis de définir les critères pris en compte dans l'attribution aléatoire des interventions de LAV aux villages de l'étude. Les principaux anophèles vecteurs ont été *Anopheles gambiae* et *An. funestus* qui représentent 2 % de la population de moustiques. Leurs taux d'agressivité respectifs ont été de 4,4 et 7,9 piqûres/personne/mois. Le nombre moyen de piqures infectantes par homme et par 100 nuits (TIE) a été de l'ordre de 2,0. La fréquence allélique du gène *kdr* a été d'environ 28 % de décembre 2007 à mai 2008. L'accès palustre a été défini sur base d'une fièvre associée à une densité parasitaire>2000 parasites/µL. La densité moyenne d'incidence clinique a été de 9 (5,6 – 13,9) accès palustres par 100 enfants-mois. La densité parasitaire moyenne des infections a été de 532 (448 – 631) formes asexuées de *Plasmodium falciparum* par µL de sang et la prévalence moyenne de l'infection entre 30,8 (26,2 – 35,4) %. Le taux moyen de couverture des moustiquaires était resté stable (92 %). Le taux moyen d'utilisation des moustiquaires a diminué de 75 % à 50 % en 7 mois d'étude. Bien que l'étude de pré intervention ait eu lieu essentiellement en saison sèche, le paludisme apparaît de type méso endémique avec un faible niveau de résistance des vecteurs aux pyréthrinoïdes. L'étude a montré une grande hétérogénéité spatiale entomologique, parasitologique et clinique. Le taux d'utilisation des moustiquaires a chuté au cours de l'étude malgré la sensibilisation périodique. L'attribution aléatoire des interventions aux villages a été basée sur le principal critère de jugement qui est l'incidence des accès palustres. Elle a permis de constituer quatre groupes d'intervention ayant des indicateurs palustres comparables de transmission (TIE), d'infection et de maladie.

SIII_O8. Evaluation communautaire de stratégies de lutte anti vectorielle contre le paludisme combinant deux traitements insecticides : étude post intervention.

A. Djènontin, B.G. Damien, M-C. Henry, F. Chandre, C. Rogier, R. Osse, J. Chabi, M. Akogbéto & V. Corbel

Mots clés : paludisme; *Anophèles gambiae*; lutte anti vectorielle; résistance, moustiquaires imprégnées à longue durée d'action; bâches murales imprégnées de bendiocarb; évaluation communautaire
Adresse : armeldj@yahoo.fr

Actuellement, les combinaisons de produits insecticides résiduels dans les habitations sont préconisées pour une meilleure efficacité de la lutte anti vectorielle. Ces combinaisons visent à interrompre la

transmission et à gérer la résistance aux insecticides chez les vecteurs. Ici, l'impact sur le paludisme d'une stratégie associant des moustiquaires imprégnées à longue durée d'action (deltaméthrine) et du bendiocarb en aspersion intra domiciliaire ou en imprégnation de bâches murales, a été évaluée au niveau communautaire, en terme de transmission et chez les enfants de 0 à 5 ans en termes d'infection et de maladie. L'essai contrôlé randomisé a été effectué dans 28 villages au sud du Bénin (population d'environ 14 000 personnes) où la résistance des vecteurs aux insecticides pyréthrinoïdes est modérée. Quatre stratégies de lutte anti vectorielle ont été mises en place : (1) une couverture sélective en MILD des enfants de 0 à 5 ans selon les recommandations du Programme National de Lutte contre le Paludisme (PNLP) (bras PNLP, témoin), (2) une couverture universelle en MILD (bras MILD), (3) une combinaison de la couverture universelle en MILD avec des bâches murales imprégnées régulièrement (tous les 4 mois) de bendiocarb à la dose de 200 mg/m^2 (bras MILD + BMIB), (4) une combinaison de la couverture sélective en MILD des enfants de 0 à 5 ans avec une Pulvérisation Intra Domiciliaire annuelle de bendiocarb à la dose de 400 mg/m^2 (bras PID). Le contrôle de qualité des interventions (mise en place et utilisation) et de la collecte des données a été périodiquement réalisé. L'analyse multi-variée des données entomologiques, parasitologiques et cliniques prenant en compte l'effet de l'âge et du type d'échantillonnage a été effectuée à l'aide de différents modèles de régression. Les taux d'inoculation entomologique observés dans les 4 bras ont été comparables, variant de 5,7 à 15,5 piqûres infectantes/personne/an ($p>0,20$). Quel que soit le traitement, la fréquence allélique du gène kdr a augmenté au cours des 17 mois d'étude (de 29% à 84% en moyenne). Toutefois, cette fréquence allélique a été significativement plus faible en fin d'étude dans le traitement MILD+BMIB (72%) que dans les 3 autres traitements (>86%) ($p<0,02$). Du point de vue clinique et parasitologique, les densités moyennes d'incidence clinique ajustées sur l'âge ont été comparables dans les 4 bras, variant entre 5,0 et 6,6 accès palustres par 100 enfants-mois ($p=0,35$). La prévalence moyenne de l'infection a été la même dans les bras PNLP, MILD et MILD+BMIB ($p>0,12$). Cette prévalence a été trouvée plus élevée dans le bras PID, comparé au bras témoin PNLP (OR=1,43 (1,05-1,93), $p=0,02$). Les densités parasitaires moyennes des infections ajustées sur l'âge ont été comparables entre les bras, variant entre 647 et 707 trophozoïtes *Plasmodium falciparum*/µL ($p=0,46$). En ce qui concerne le taux de couverture des MILD, il s'est révélé satisfaisant (80%), mais le taux d'utilisation (40%) était bien inferieur au seuil fixé dans l'étude (70%). L'essai contrôlé randomisé réalisée au sud du Bénin n'a pas montré de bénéfice en termes de protection contre le paludisme quand deux traitements insecticides résiduels à l'intérieur des habitations sont combinées. Plusieurs facteurs peuvent expliquer ces résultats comme des facteurs anthropo-sociologiques (perception et utilisation des traitements), opérationnels (effet résiduel du bendiocarb, couverture des bâches), biologiques (comportement des vecteurs) et épidémiologiques (faible niveau de transmission dans la zone d'étude). Il est nécessaire de reproduire de tels essais contrôlés randomisés dans d'autres faciès épidémiologiques afin de renseigner les acteurs de santé publique sur les stratégies à mettre en œuvre pour améliorer les actions de la lutte contre le paludisme.

SIII_O9. Modéliser le risque de transmission du paludisme dans un contexte de lutte anti vectorielle : enjeux et méthode.

N. Moiroux, V. Corbel & H. Guis.

Mots clés : Modélisation; vecteurs; Lutte anti vectorielle; SIG; Télédétection
Adresse : nicolas.moiroux@ird.fr

Les mesures des contacts hôtes-vecteurs (captures) et de l'impact parasito-clinique (morbidité) du paludisme réalisées dans le cadre du projet REFS (évaluation de méthodes de lutte anti-vectorielle en phase 3) montrent une grande hétérogénéité spatiale (intra et inter-villages). Particulièrement, les captures révèlent une variation spatiale (semble-t-il assez ségrégative) de la diversité spécifique (2 espèces et 2 formes moléculaires de vecteurs aux compétences vectorielles différentes). Par ailleurs, la résistance aux insecticides (et notamment la mutation génétique L1014F de résistance aux pyréthrinoïdes) n'est pas présente aux mêmes fréquences chez les différentes espèces et formes

moléculaires d'Anophèles. Nous observons également une répartition spatiale hétérogène de cette mutation sur notre zone d'étude. Cette hétérogénéité est également décrite à une échelle nationale (Djogbenou, *et al.* 2010, Yadouleton, *et al.* 2010) ou continentale (Ranson *et al*, 2010. A paraître). Nous souhaitons ici élaborer une méthode de modélisation spatiale ayant les objectifs suivants :
- Améliorer les connaissances sur les preferenda écologiques de 4 espèces et formes moléculaire de vecteurs.
- Comprendre la répartition des la mutation L1014F.
- Mesurer les effets des différentes méthodes de lutte anti-vectorielle.

Nous proposons d'identifier les indicateurs du risque de transmission lié aux différentes espèces et formes moléculaires de vecteurs anthropophiles. Les modèles statistiques utilisés seront construits sur la base de données spatialisées de terrain, de laboratoire et dérivéesd'images satellites décrivant l'environnement, les vecteurs (résistance) et les hôtes (lutte anti-vectorielle). Les indicateurs identifiés seront intégrés dans un modèle mathématique global spatialisé du risque de type Capacité Vectorielle.

SIII_O10. Réponse anticorps anti-protéines salivaires d'Anophèles : nouveau bio marqueur évaluant l'efficacité de la lutte contre les vecteurs du paludisme.

P.M. Drame, A. Poinsignon, S. Cornelie, P. Besnard, J. Le Mire, J-C. Toto, M. Sembene, F. Fortes, P. Carnevale & F. Remoue.

Mots clés : Réponse anticorps; protéines salivaires; bio marqueur; efficacité; lutte anti vectorielle.
Adresse : papa-makhtar.drame@ird.fr

Pour un meilleur contrôle du paludisme, plusieurs efforts sont déployés pour développer de nouveaux outils permettant d'évaluer l'efficacité des stratégies anti-vecteurs. Il a été montré récemment que l'étude de la réponse anticorps (Ac), chez l'homme, dirigée contre les protéines salivaires d'Anophèles représente un marqueur d'exposition aux piqûres de cet arthropode hématophage. L'objectif de cette étude était de vérifier si ces réponses IgG spécifiques aux protéines salivaires d'Anophèles pourraient représenter de nouveaux indicateurs d'efficacité de stratégies de lutte anti-vectorielle (LAV), et notamment de l'utilisation des moustiquaires imprégnées d'insecticides (MIIs). Cette étude clinique (phase III) évaluant l'efficacité de MIIs (Permanet®) a été menée en Angola (localité de Lobito) chez 21 familles (n=230 ; enfants/adultes) suivies pendant deux ans (mars 2005 ? janvier 2007). Leurs niveaux de réponses IgG anti-salive totale d'Anophèles gambiae et spécifique au peptide P1 de la protéine salivaire gSG6 (spécifique à Anophèles) ont été évalués (par ELISA), puis comparés aux données entomologiques (captures d'*An. gambiae*) et parasitologique recueillies (prévalence et densités parasitaires). Nous avons ainsi constaté, avant la mise en place des MIIs: 1) d'importantes variations, chez toutes les tranches d'âge (0-6 ans, 7-14 ans ou >14 ans), des réponses IgG anti-salive totale d'*An. gambiae* et anti-gSG6-P1 en fonction de la saison de transmission; 2) une bonne association entre les résultats IgG anti-salive totale et anti-gSG6-P1 et les données entomologiques et parasitologiques. Juste après la mise en place des MIIs, une forte baisse des réponses IgG a été observée et était associée à la chute de la parasitémie. De manière très claire, les résultats des dosages d'IgG anti-gSG6-P1 montraient une chute des niveaux individuels d'IgG spécifiques plus marquée après la mise en place des MIIs. De plus, une différence significative de niveau de réponse IgG entre les classes d'âge n'a été observée qu'avec le peptide gSG6-P1 lors des périodes de forte exposition. En conclusion, l'obtention d'un tel bio marqueur peut, en plus d'être un marqueur d'exposition à la piqûre du vecteur, servir à évaluer l'efficacité des MIIs et plus largement des différentes stratégies anti-vecteurs du paludisme (REFS) et autres maladies vectorielles. Il pourrait aussi servir à la cartographie des zones à risque palustre.

Posters

SIII_P1. Optimization of odour-baited resting boxes for sampling malaria vector, *Anopheles arabiensis* Patton in Africa

E. J. Kweka, B. J. Mwangonde & A.M. Mahande

Key words: rest boxes; odour; *Anopheles arabiensis*; Tanzania
Address: pat.kweka@gmail.com

Odour baited resting boxes are simple, reliable and important tool of sampling malaria vector mosquitoes for surveillance and control programmes in different parts of Africa. To optimize the use of cow urine baited resting boxes for sampling An.arabiensis, community based study was conducted at Lower Moshi irrigation schemes. Latin square experiments involving four houses were conducted overnight for twenty days to assess the effect of different parameters for the collection of mosquitoes using odour baited resting boxes: i) if odour baited traps perform better indoors or outdoors, and or if the people sleeping indoor had any effect on mosquitoes density collected: ii) The best height of collecting mosquitoes indoors and outdoors and: iii) efficiency of trap in three different distances from the house wall outdoors. A total of 8581 mosquitoes were sampled by odour baited resting boxes, of which, 8051 (93.82%) were *An.arabiensis* and 530 (6.18%) *Culex quinquefasciatus.* Light trap collected a total of 12,420 of which 9442 (76.02%) were *An.arabiensis*, 126 (1.01%) *An. funestus*, 230 (1.85 %) *An. rufipes* and 2622 (21.11%) *Cx. quinquefasciatus.* The best height for outdoor mosquitoes sampling was 15 cm and 220 cm while indoors was 105 cm. The difference in mosquito collection between different outdoor and indoor heights was statistically insignificant. The optimal location of odour baited resting boxes from house walls outdoors was 3m. In conclusion the odour baited resting boxes have shown a significant performance in community level for sampling wild populations of *An.arabiensis*, one of the major malaria vector in Africa. The indoor best performance had not been affected by sleeping human odours. This gives opportunity for optimizing this sampling tool in surveillance and control programmes.

SIII_P2. Awareness, Possession and Use of Insecticide Treated Nets for Prevention of Malaria in Children Under-Five in Abeokuta, Nigeria.

O. A. Idowu, S. O. Sam-wobo, A. S. Oluwole & A. S. Adediran.

Key words: Insecticide Treated Net (ITN); Children Under-five; Traditional Birth Home; Hospital; Malaria
Address: tomiwo2@gmail.com

A survey was carried out to assess awareness, possession and use of Insecticide Treated Net (ITN) by mothers in preventing malaria among children under-five. Malaria though was considered dangerous by almost all respondents (98.5%); the level of awareness of ITN as a malaria preventive tool was 75.1% while possession was 45%. Awareness and possession of ITN were positively and significantly influenced by high educational qualification of mothers and attendance of a public hospital for antenatal care. Hospitals were identified as the major source of awareness among respondents; Women that delivered their babies in Traditional birth home displayed least awareness (38.6%) and recorded low possession (10%). There was no significant relationship between ITN usage, birth order and age of child. Heat experienced while sleeping under ITN and problem of how to hang the net were major limitations identified in the use of ITN. The need to involve women receiving antenatal care outside the hospital in malaria control intervention is hereby recommended.

SIII_P3. Innovative tool to evaluate malaria risk: Toward the development of a biomarker of infecting bite

C. Sylvie, M. Rossignol, E. Demettre, M. Seveno, F. Chandre, V. Corbel, I. Morlais & F. Remoue.

Key words: biomarker; malaria; infecting bite; salivary proteins; vector control

Address: sylvie.cornelie@ird.fr

Malaria causative agent, i.e. plasmodium parasite, is transmitted to human during the blood meal of the anopheles mosquito. During blood feeding, the vector injects parasite and saliva into the vertebrate host skin. This saliva contains bioactive components which induced an immune response in the vertebrate host. Our team has previously developed a serological biomarker to assess the human exposure to mosquito bite. This tool is based on the evaluation of human antibody response against mosquito salivary proteins. Here we investigate whether a salivary antigen could be specific of the infecting bite and constitute a biomarker of the risk of disease. To assess this question, we have compared the human antibody response against salivary extracts infected or not by *P. falciparum.* Experimental infections of *An. gambiae* by *P. falciparum* were carried out and salivary glands were dissected 14 days post-infection. The infective status of each salivary gland was confirmed by PCR. Then two-dimensional western-blots were realized with different pools of infected vs. non infected sera. These pools were constituted with sera from Senegalese 1-2 y.o. children leaving in deeply exposed village to anopheles and presenting or not a high parasitemia. The results of 2D-blots showed that immunogenic proteins around 70kDa are detected in both infected and non infected vector. Mass spectrometry analyses identified these proteins as the 5nucleotidase and Apyrase, proteins which inhibiting platelet aggregation. Furthermore one immunogenic protein from infected salivary glands extracts was detected only with sera from infected children by *P. falciparum.* Mass spectrometry analysis on this protein is underway. These results indicated that human immune system could discriminate between an infective bite and a non infective bite. This work opens the way to design epidemiological tools to evaluate the risk of malaria in area of (re) emergence, but also have strong implications for the vector control and monitoring.

SIII_P4. Potential contribution of asymptomatic individuals to malaria transmission in highly endemic areas in Cameroon: Gametocyte prevalences and densities in different age groups.

E. J. Eyong, H. Kimbi, F. Wanji , J. L. Ndamukong-Nyanga, E. Lum, O. J. A.Kengne, N. Tendongfor & S. Wanji

Key words: Asymptomatic; malaria; highly endemic; Gametocyte

Address: jeebanga@yahoo.com

This study was designed to investigate the potential contribution of asymptomatic individuals (excluding children < 5 years and pregnant women) to malaria transmission in highly endemic areas in Cameroon. Additional objectives included the influence of age and sex on the prevalences and densities of both asexual and sexual stages in the sampled individuals. Blood samples were collected by pricking the finger. Thick and thin blood films were prepared and Giemsa-stained. Slides were observed under x 100 objective of the microscope for the detection of asexual and sexual stages of *P. falciparum.* Quantification of asexual stages and gametocytes was done against 200 WBC and 500 WBC, respectively, assuming a WBC of 8000 leucocytes/µl blood. The data was analyzed using SPSS version 15. Overall, 1398 individuals aged 5-80 years were enrolled in the study. The overall prevalence of asexual stages was 80.2% (1121/1398) while that of sexual stages was 11.2% (125/1121). The highest prevalence (99.1%, 113/114) of asexual stages was recorded in the age group 16-20 years, while the lowest prevalence (59.9%, 214/357) was recorded in the age group 5-10 years ($p = 0.00$). The highest prevalence (28.5%, 61/214) and the lowest prevalence (1.5%, 1/68) of sexual stages were recorded in age groups 5-10 years and 31-35 years, respectively ($p = 0.00$). Sex did not influence significantly the

prevalence of asexual stages and sexual stages ($p>0.05$). Age and sex did not influence significantly the GMPD of asexual and sexual stages ($p > 0.05$). The majority of individuals (59.2%, 74/125) who carried sexual stages had asexual densities between 500-5000 parasites/µl blood. The distribution of gametocyte carriers in the different asexual stage groups was not statistically significant ($p = 0.19$). In conclusion in areas of high malaria endemicity of Cameroon, the prevalence of gametocytes is heterogeneously distributed amongst different age groups of asymptomatic individuals (excluding children < 5 years and pregnant women). Individuals of age groups 5-10 and 11-15 years carry more gametocytes and are likely to contribute more to malaria transmission through mosquito bites. Thus, they could constitute the most rational and useful strategic group for malaria interventions.

SIII_P5. Etat des lieux des stratégies de lutte contre le paludisme Bénin 200-2009 et défis pour les OMD.

K. A. T.Daye; S. Bio-Bangana & M. Akogbeto

Mots clés : Paludisme; Défis ; OMD ; Bénin.

Adresse : kwamidaye@yahoo.fr

Maladie infectieuse parmi les plus anciennes dans les sociétés africaines, le paludisme reste encore responsable de bien des souffrances et décès et impose une charge économique bien trop lourde. Chaque année, de 300 à 500 millions de cas de paludisme clinique et plus d'un million de décès imputables au paludisme sont notifiés à l'OMS, à l'échelle mondiale, et environ 80% de cette charge est enregistrée en Afrique, coûtant plus de 12 milliards $US par an et ralentissant la croissance économique de 1,3% par an. En Afrique, l'Egypte, le Maroc et le Cap Vert n'ont qu'une transmission résiduelle du paludisme alors que l'Algérie, le Lesotho, l'Ile Maurice et les Seychelles sont exempts de paludisme. Par contre, le paludisme reste endémique à des intensités variables dans tous les pays de l'Afrique subsaharienne. Au Bénin, le paludisme est un véritable problème de santé publique et constitue la première cause d'hospitalisation, de morbidité et de mortalité maternelle et infantile. Dans le but de vaincre cette maladie, le Bénin à l'instar des autres pays d'Afrique où sévit le paludisme, a adopté la politique d'éradication préconisée par l'OMS. Trois ans après la mise en œuvre de cette politique, force est de constater que malgré les différentes interventions dans la lutte, le paludisme n'est pas éradiqué. Le but de notre communication est de contribuer à une connaissance sur la lutte contre le paludisme en faisant un état des lieux des différentes stratégies adoptées dans la lutte contre le paludisme au Bénin les dix dernières années et les défis à relever pour l'atteinte des Objectifs du Millénaire pour le Développement. Après une présentation du Bénin et son profil épidémiologique, nous exposerons la situation de lutte contre le paludisme, les différentes stratégies adoptées ainsi que les défis à relever. Nous terminerons notre exposé par quelques recommandations dans la perspective de mieux contrôler cette pandémie au Bénin.

SIII_P6. Malaria vectors in the guinea savanna zone of Central Nigeria.

I.S. Ndams, I. H. Nock, C. G. Vajime & J. E. Conn.

Key words: Malaria vectors; *Anopheles gambiae* s; Plasmodium; circumsporozoite; molecular forms

Address: isndams@abu.edu.ng

The composition of anopheline mosquitoes in the guinea savanna zone of Nigeria and their infection with the malaria Plasmodium parasite has been investigated. Adult Indoor resting anophelines were caught using mechanical aspirator from human habitations. Three species of Anopheles were morphologically identified as *An. funestus*, *An. rufipes*, and *An. gambiae* s.l. PCR diagnostic assays delineated *An. gambiae* s.s. and *An. arabiensis* from the gambiae complex and further delineated the M and S molecular forms of gambiae s.s. All the 1058 individuals collected were tested for Plasmodium

sporozoites by ELISA. *Anopheles gambiae* s.s. was the most dominant species (66.64%; n=705) followed by *An. arabiensis* (19.00%; n=201), *An. rufipes* (10.68%; n= 113) while *An. funestus* was the least (3.69%; n= 39). The composition of the S form was dominant (60.39%; n=407) over the M form (39.07%; n=261). 23 (2.17%) of the 1058 anopheline tested were positive to Plasmodium circumsporozoite antigen; 19 (1.80%) were positive for *Plasmodium falciparum* and 4 (0.38%) for *P. malariae.* Species wise, 19 *An. gambiae* s.s were infected with Plasmodium circumsporozoite antigen; 2.27% (n=16) and 0.43% (n=3) tested positive for *P. falciparum* and *P. malariae* respectively. For *An. arabiensis* 4 were positive for circumsporozoite antigen with a 1.49% (n=3) and 0.50% (n=1) for *P. falciparum* and *P. malariae*, respectively. Seven (2.68%) of the 261 M form tested positive to *P.falciparum* only while 9 (2.23%) and 3 (0.74%) of the 407.S form tested positive to *P. falciparum* and *P. malariae*, respectively. This study shows that *An. gambiae* s.s. (the S form in particular) and *An. arabiensis* have been incriminated as the principal vectors of malaria in the guinea savanna vegetation zone of central Nigeria.

SIII_P7. Mosquitocidal and antifecundity effects of Coumarin and betulinic acid isolated from *Cassia siamea* (Fabaceae) stem bark chloroform extract on female *Anopheles stephens* (dipteria cilicidae).

G. F. Nsonde-Ntandou, L. Lucantoni , J.T. Banzouzi , M. Ndounga, S. Yerbanga, J. M. Ouamba, A. Habluetzel, F. Esposito & A. A. Abena

Key words: malaria; mosquitoes; plant; mosquitocidal; antifecundity
Address: nsonde_ntandou@yahoo.fr

Mosquito acts as vector for various pathogens that cause malaria, filariasis, schistosomiasis, Japanese encephalitis, and yellow fever. Mosquito control is the key measure to prevent spreading these diseases are still a major problem in the developing countries. The strategy against malaria in the world includes the destruction by chemical, physical or biological agent of larvae, adult mosquitoes and reducing the number of human mosquito's contacts. In order to develop new tools to fight against malaria, the bark of *Cassia siamea* were subjected to phytochemical investigation, which led to the isolation of coumarin and betulinic acid. We conducted a chronic administration in the form of food from the chloroform extract of the coumarin and betulinic acid at the concentrations of 2000, 800 and 1600 ppm respectively, once every two days, for 21 days corresponding to the sporogonic cycle. The results have shown an efficiency of 100% mortality in the group of mosquitoes treated by coumarin on day 15, an efficiency of 90% mortality in the group of mosquitoes treated with betulinic acid at day 20, finally efficiency 71% mortality in the group of mosquitoes treated with the chloroform extract. The sporogonic cycle duration, was evaluated at 21 days. Coumarin and betulinic acid have reduced the fecundity of females mosquitoes half.

SIII_P8. The efficacy of extracts of the plant *Argemone mexicana* on mosquito species, *Anopheles arabiensis.*

N.A. Alfahal, H. Abdelgader & S. A. Elhossain

Key words: Malaria vector; insecticide resistance; Botanical extract; Sudan; Gezira
Address: Abdelgaderh@yahoo.com

The development of malaria control in irrigated Schemes of Central Sudan has gone through several phases. As a result of agricultural and irrigation practices in the Gezira, *falciparum* malaria transmission became perennial instead of seasonal and the mosquito vector developed resistance to several insecticides. Subsequent failure to maintain control led to serious epidemics. A new control strategy of the vector is essential to help in insecticide resistance management. In the recent study extracts of the plant *Argemone mexicana* were selected to investigate their larvicidal potential against

mosquito. Laboratory reared mosquito species, *Anopheline arabiensis* were used in the study. Bioassay of leaves and seeds extracts of A. Mexicana were tested in the laboratory. The results showed that the LC50 and LC90 of the leaf extract were 0.16% and 0.39%, respectively. The Slop of the Ld-p line was relatively steep (5.44) indicating a homogenous response. The LC50 (0.006%) and LC90 (0.061%) of seed extract (EC formulated) were found to be lower than in leaf extract and the slope of the Ld-p line was 1.27. The mortality observed after exposing larvae of mosquito to
the concentration of 0.25% of seed extract were found to be 0, 25% and 100% after ½ h, 1.5 h and 6.5 hours of exposure. In case of leaf extract the mortality of tested larvae were 0%, 25% and 95% after 1 h, 6.5 h and 18.5 h of exposure.

SIII_P9. Activités répulsives et insecticides de plantes contre les vecteurs du paludisme en zone rurale au Burkina Faso.

D. W. Wangrawa, A. Sanon, A. Badolo, M.W.Guelbeogo, R. Nebie & N.F. Sagnon

Mots clés : huiles essentielles; larvicide; ovicide; répulsif; *Anophèles gambiae*
Adresse : wangrawad@yahoo.fr

Les moustiques sont sources d'énormes nuisances vis-à-vis des hommes. Vecteurs de plusieurs maladies, ils font l'objet de plusieurs luttes. Dans notre étude nous avons évalué l'effet corvicide, larvicide et répulsif de quatre plantes sur l'un des vecteurs majeurs du paludisme qu'est *Anophèles gambiae* s.l. Ces plantes sont *Ocimum canum*, *Hyptis suaveolens*, *Hyptis spicigera* et *Lantana Camara.* Les feuilles et les tiges de ces plantes, cueillies à l'état jeune, ont été utilisées pour l'extraction des huiles essentielles qui ont servi aux tests. Les œufs et les larves soigneusement triés ont été soumis à différentes concentrations. L'évolution de la vie des larves et de l'éclosion des œufs à été suivie pendant 72h. Toutes les huiles ont manifesté une activité larvicide tandis qu'au niveau des œufs seuls *H.suaveolens* et *Lantana Camara* ont manifesté une activité corvicide. A la dose de 200 ppm *L. Camara* tue 100% des larves après 72h de contact, *H. spicigera* et *O.canum* tuent environ 90% des larves et *H.suaveolens* tue 60% des larves après 72h. Aux concentrations de 25 ; 50 et 100 ppm la mortalité est relativement faible attestant ainsi que la mortalité est dose-dépendante avec les larves. Toutes les huiles manifestent une activité larvicide pendant les 72h mais cette activité baisse sensiblement après 48h. L'effet répulsif de ces huiles à été évalué sur 100 moustiques femelles contenus dans une cage selon la méthode des bras séparés. Toutes les huiles ont été répulsives des moustiques surtout à la concentration de 500ppm. L'indice de protection calculé à cet effet est dose dépendante. Les doses efficaces calculées attestent que *H.suaveolens* et *O.canum* sont les plus efficaces pour la répulsion des moustiques femelles *d'Anopheles gambiae* s.l.

SIII_P10. Prévalence du paludisme dans trois zones rurales avec ou sans intervention d'un programme de lutte.

F.A.Ouattara, G.B. Koudou, G. Raso, U. Utzinger & M. Dagnogo.

Mots clés : Paludisme; Prévalence; test diagnostique rapide
Adresse : oufouala@yahoo.fr

Pour évaluer l'impact des interventions du programme national de lutte contre le paludisme par la distribution des moustiquaires imprégnées longue durée (MIILD) et l'approche intégrant l'utilisation des nouveaux tests diagnostiques rapides (TDR) du paludisme à une haute couverture en MIILD, trois villages dont deux ayant bénéficiés des interventions dudit programme et un témoin ont été choisie. Les personnes participant à cette étude ont un âge compris entre 0 et 15 ans. Avec l'aide d'un infirmer, le diagnostique symptomatique du paludisme a été établi individuellement sur la base de la présence de fièvre (température>=37,5°C) couplé à la splénomégalie et la pâleur des conjonctives. Des gouttes

épaisses et des frottis sanguins ont été réalisés et lue au microscope par des techniciens. Simultanément, les tests de diagnostic rapide (TDR) ont été effectués. Les données ont été analysées avec le logiciel R. La prévalence du paludisme obtenue était élevée à Yoho (microscopie : 64,48% ; TDR : 65,52%). Elle variait entre 37,5% de 63,45% pour la microscopie puis entre 46,71% et 67,27% pour le TDR. Le taux le plus élevé était déterminé dans la tranche d'âge de 5 à 10 ans. La prévalence était fortement influencée par le village (p=0,002) et l'âge (p<0,001) des patients lorsque les examens étaient réalisés par microscopie. Cependant, les examens faits par le TDR étaient seulement influencés par l'âge (p<0,001). Il y avait aucune différence significative entre la prévalence des personnes ayant dormis sous MIILD la veille (microscopie p=0,99 ; TDR p=0,91) et entre les sexes (microscopie p=0,63 ; TDR p=0,51). Par ailleurs, la densité parasitaire était influencée par l'âge (p<0,05), la température (p=0,03) et le village (p<0,05). Cependant le fait d'avoir dormis sous MIILD la veille (p=0,41) et le sexe (p=0,60) n'avaient aucune influence sur la densité parasitaire. Certes le TDR est un moyen de détermination prématuré des cas de paludisme, cependant elle pourrait jouer un rôle important dans l'évaluation rapide des méthodes de lutte contre le paludisme et avoir un effet sur la réduction de la prévalence pourvu qu'il soit associé à une autre méthode.

SIII_P11. Profil entomologique du paludisme en Côte d'Ivoire 1956-2009.

Al. A Koffi, L. P Ahoua Alou, M. A Adja, L. Y Konan, M. K San, M. Koné, D.Kouassi, L. Manga, M. Bagayoko & P. Bitsindou

Mots clés : Profil entomologique du paludisme; documents de référence; lutte anti vectorielle; transmission du paludisme; sensibilité aux insecticides
Adresse : koffi_alphonsine@yahoo.fr

La lutte contre le paludisme repose actuellement sur la prise en charge précoce et correcte des cas et la lutte anti-vectorielle (LAV). Toutefois, la LAV nécessite de disposer d'une base d'informations entomologiques fiables dont le système de recueil des données sanitaires ne prend pas en compte. Pour pallier ce déficit, le Bureau Régional de l'Organisation Mondiale de la Santé pour l'Afrique a entrepris la réalisation de document retraçant le profil entomologique du paludisme des pays africains. Ces profils sont des documents de référence pour la planification et l'évaluation des interventions de LAV contre le paludisme. En Côte d'Ivoire, le profil a été réalisé en collaboration avec l'IPR/INSP et le PNLP. Il a consisté à répertorier et à analyser des données bibliographiques provenant de différents supports (rapports, mémoires, thèses, publications scientifiques) relatives à la distribution des vecteurs, leur sensibilité aux insecticides et les indices de transmission du paludisme. Les documents exploités au nombre de 77 ont été produits entre 1956 et 2009. Ces données concernent 206 sites étude inégalement répartis dans les 19 régions du pays. Il est à noter que 8 régions n'ont qu'un ou deux sites d'étude indiquant que ces régions restent à prospecter. La transmission du paludisme est assurée par trois vecteurs : *Anophèles gambiae* s.l. *An. funestus* et *An. nili.* On observe une diversité dans l'épidémiologie du paludisme. La sensibilité aux insecticides a été étudiée essentiellement sur des populations d'*An. gambiae* s.s, vecteur majeur du paludisme. Il en ressort que la résistance aux pyrétrinoïdes est largement répandue sur l'ensemble du pays. Les niveaux de résistance les plus élevés sont observés en zone cotonnière au Nord du pays et dans les milieux urbains de Bouaké, Abidjan et San-Pedro. Ces résultats révèlent des acquis entomologiques dans certaines régions du pays mais également les gaps à combler afin de disposer d'une base de données nationales complètes et actualisées. Avec l'accès universel aux moustiquaires imprégnées d'insecticides, il est nécessaire de mener des investigations nouvelles et/ou d'actualiser certaines informations entomologiques existantes. La surveillance de la résistance des vecteurs aux insecticides est une nécessité au maintien de l'efficacité de la LAV.

SIII_P12. Etude de l'influence du génotype hémoglobinique sur la transmission de Plasmodium de l'homme aux moustiques.

L. C. Gouagna, G. Bancone, F. Yao, B. Yameogo, K. R. Dabiré, C. Costantini, J. Simporé, Jean Bosco Ouedraogo & David Modiano

Mots clés : paludisme; gamétocytes; *Plasmodium falciparum*; moustiques; génotypes
Adresse : Franck Yao: frayao@yahoo.fr

Dans le présent travail, nous avons étudié l'influence des facteurs génétiques de l'homme (notamment le polymorphisme de la chaîne bêta de l'hémoglobine) sur la transmission du Plasmodium de l'homme aux moustiques à Soumousso. Dans la population générale, l'hémoglobine A était majoritairement représenté par le génotype AA (71,9%) suivi du génotype AC (19%), AS (7,3%) et du génotype CC (1,6%) qui était très faiblement représenté. L'exploration des indices parasitaires sur l'examen microscopique des gouttes épaisses nous a révélé une relation, bien que faible, entre les prévalences parasitaires et le génotype de l'hémoglobine. Mais en dehors du génotype hémoglobinique, l'âge a une influence significative sur la prévalence et la parasitémie, mais surtout des gamétocytes. Soixante enfants de génotypes différents ont été sélectionnés puis répartis en groupes de 4 enfants selon leur âge (de 3-12 ans) et leur sexe. Ces enfants ont été ensuite invités à dormir dans les cases sentinelles pendant 4 nuits successives. Après chaque nuit, les moustiques présents dans chaque case étaient capturés à 6h du matin et gardés dans des cages jusqu'au septième jour avant d'être disséqués pour la recherche d'oocystes. Ce xénodiagnostic et les infections expérimentales complémentaires nous ont permis de démontrer que l'infectivité des individus pour les moustiques révélée par la présence d'oocystes dans l'estomac des moustiques était fonction du génotype ($P<0,05$). En définitive, en plus des études précédentes démontrant une protection conférée par l'HbC et l'HbS contre le paludisme, notre étude souligne une coévolution entre l'hôte humain et le parasite responsable du paludisme, *P. falciparum.* Par de simples mutations de l'hémoglobine, un avantage mutuel s'est établi entre le couple hôte-parasite: une résistance plus élevée à la maladie pour l'hôte, et une transmissibilité accrue pour le parasite.

SIII_P13. Statut énergétique des mâles d'*Anophèles gambiae*, état physiologique et source de repas sucré.

M.H.Gouagna, C.Diabaté, A. Simard & F.K. R.Dabire

Mots clés: *Anopheles gambiae*; males, sugar-feeding, survival, energy
Adresse: maigahamid@yahoo.fr

Nutritional reserves accumulated during larval development and from sugar-feeding as adults are critical determinants of adult survival and mating mosquito species. For instance, lipids are required for long maintenance and primarily acquired from feeding during the larval development. Flight is a requirement for mosquito mating and this activity is fuelled by sugars or glycogen derived from sucrose or its components fructose and glucose. Understanding how the larval and adult nutrition of mosquitoes could be managed to produce males with optimal development will be highly beneficial. Current study looked at the variation of energetic reserves in mosquitoes from field or those reared in the laboratory and evaluated the impact of different sugar-plants on *Anopheles gambiae* males survival. Batches of 20 - 25 males derived from insectariums were continuously fed for 6 days on flowers of *Mangifera indica*, *Thevetia nerifolia* and *Senna siamea* for the survival tests. Three groups composed of unfed, glucose 6 % fed and distilled water fed males were used as controls. The energetic reserves (sugars, glycogen, lipids and proteins) were estimated in these mosquitoes using spectrophotometer. Unfed newly emerged males from the field as well as swarming and resting males were collected in the field and tested as well. Males fed on *M. indica* flowers and glucose (6%) had a better survival (respectively 87% and 86%) followed by those fed on *T. nerifolia* (55%) and *S. siamea* (3%).Unfed males and those fed on distilled water did not survive over 3 days. Males that had fed only on distilled water, died quickly after loosing 50 % of their metabolic contents. A significant variation of the energetic

reserves (sugar) was observed in swarming males. Our results suggested that sugars and glycogen are the main energy source involved in flight and sugar and lipids for resting metabolism. This study stresses the role of sugar meals and their quality on males survival and flight activities and is important for the implementation of genetic approaches to control malaria vectors.

SIII_P14. Distribution spatiale, caractérisation des gîtes, dynamique des formes moléculaires d'*Anophèles gambiae* suivant un gradient d'urbanisation à Yaoundé.

B. F. Tene, C. N. Antonio, C. Kamdem, P. Bousses, D. Fontenille, N. J. Besansky & C. Costantini

Mots clés : gîtes larvaires; formes moléculaires M&S d' *Anopheles gambiae*; gradient d'urbanisation
Adresse : billytene@gmail.com

L'urbanisation croissante en Afrique attire l'attention des responsables de santé publique sur le paludisme urbain, et elle soulève la question de savoir si les vecteurs de paludisme ont la capacité de s'adapter aux facteurs de stress environnementaux produits dans les villes densément peuplées. Dans le domaine de forêt du sud Cameroun, les formes moléculaires M et S de l'espèce *Anophèles gambiae* sont réparties le long des gradients d'urbanisation, suggérant qu'un processus d'adaptation pour la forme de M à l'environnement urbain soit en cours (Kamdem *et al.*, soumis). Ce processus est vraisemblablement dû à la capacité des larves de M à se développer avec succès dans les habitats urbains pollués. Pour caractériser les biotopes larvaires de M et S et leur dynamique, nous avons mené une étude longitudinale des gites larvaires d'*An. gambiae* pour évaluer leur distribution et le rapport avec des activités humaines dans la capitale Yaoundé et son voisinage périurbain. Un total de 2449 gites potentiels ont été examinés, dont environ 20% contenant des larves d'*An. gambiae*. Les gîtes larvaires des anophèles étaient plus abondants en milieu urbain comparé aux secteurs ruraux ou suburbains. Des fluctuations saisonnières dans la disponibilité des gîtes ont été davantage prononcées dans le secteur rural qu'urbain. Les torrents ruisselants et les marais étaient associés à des densités larvaires très basses ou nulles. Des activités humaines telles que le maraîchage, le logement dans des secteurs marécageux, et les chantiers de construction ont été associés aux sites de ponte d'*An. gambiae*. Inopinément des larves de *An. gambiae* ont été trouvées dans des gîtes urbaines fortement pollués de matière organique. L'identification par PCR a indiqué que seule la forme moléculaire de M était présente dans les localités les plus urbanisées, tandis que la forme de S était de loin la plus abondante dans les emplacements ruraux, les suburbains étant transitoires entre ces extrémités. Ces résultats fournissent l'évidence que le vecteur du paludisme *An. gambiae* s.s. s'adapte aux eaux urbaines résiduelles, et partitionne clairement la distribution des formes moléculaires M et S entre les secteurs urbains et ruraux.

SIII_P15. Latest update on the biology and genetics of *Anopheles nili* group of malaria vectors in Subsaharan Africa.

C. Ndo, C. Antonio-Nkondjio, I. V. Sharakhov, A. Cohuet, P. Kengne, I. Morlais, P. H. Awono-Ambene, P. Ngassam, D. Fontenille & F. Simard.

Key words: *Anophèles nili* ; malaria; Biology;; subsaharan Africa
Address: cyrndo@yahoo.fr

Mosquitoes from the *An. nili* group are widespread in tropical Africa, where they efficiently transmit malaria to humans, particularly along fast running rivers were their larvae develop. However, despite this important epidemiological role, they remained not well studied. Hence, major gaps in their biology, ecology and genetics still exist and need to be addressed for the development of innovative, sustainable and integrated control strategies toward these vectors. Here, new data on *An. nili* group are presented. In order to determine key factors associated with the distribution of *An. nili* species,

bionomic studies consisting in the sampling and analysis of physico-chemical parameters from over twenty river networks across Cameroon were undertaken. In addition, a large scale population genetic study was conducted across Sub-Saharan Africa using eleven microsatellite markers and sequence variation in four genes within the 28s ribosomal subunit and mitochondrial DNA, and cytogenetic. The distribution of *An. nili* s.s. conformed to that of a generalist species which is adapted to exploiting a variety of environmental conditions, justifing its presence in both forest and savanna areas. Whereas, *An. ovengensis* and *An. carnevalei* appeared as specialist mosquitoes and are confined in forest environment. All the markers used showed high levels of genetic differentiation between species ($Fst>0.281$, $P<0.001$). Within the major vector An. nili s.s., low level of genetic differentiation was observed between populations from Senegal to Cameroon ($Fst<0.022$). However, high genetic differentiation probably resulting from demographic and/or selective events was associated with forest populations (East Cameroon and DRC). These data were consistent with sequence analysis indicating that populations from East Cameroun belong to new molecular forms never described within the group. A new cytological map of *An. nili* s.s was described with two polymorphic inversions in 2R arm found in populations from Burkina Faso and Cameroon. *An. nili* species distribution along river systems was highly correlated with environmental variables. Genetic studies suggested that additional species exist within the *An. nili* group. Availability of the new polytene chromosome map, polymorphic inversions, and physically mapped DNA markers for *An. nili* s.s. will further stimulate population genetic, taxonomic, and genomic studies of this neglected vector.

SIII_P16. Etude de l'environnement physique et sociologique du paludisme en prélude à une pulvérisation intra domiciliaire au Bénin.

G. G. Padonou, G. L. Gbédjissi, H. S.Bankolé, A. Yadouléton, H. Noukpo & M. C. Akogbéto

Mots clés : Paludisme; *Anophèles gambiae*; bendiocarb; habitations
Adresse : pagergil@yahoo.fr

Le paludisme est le principal problème de santé publique en Afrique. Au Bénin, il est la maladie la plus importante enregistrée dans les formations sanitaires et son vecteur *Anophèles gambiae* est résistant aux pyréthrinoides. Pour cela, le Programme National de Lutte contre le Paludisme (PNLP) s'est engagé dans une stratégie de lutte intégrée dont les pulvérisations intra domiciliaires (PID) à base de bendiocarb, dans les communes d'Adjohoun, Dangbo, Misséréte et Sèmè. En prélude à cette pulvérisation, une étude de l'environnement physique et sociologique du paludisme a été effectuée pour identifier les éventuelles limites au succès de cette stratégie.Le protocole est basé sur la collecte de données quantitatives et qualitatives en avril 2008, dans les communes d'Adjohoun, Dangbo, Missérété et Sèmè. 3228 hommes et femmes adultes responsables de leur ménage ont été soumis à des questionnaires portant sur leurs connaissances et différentes pratiques de prévention et de prise en charge du paludisme. Des observations directes sur le terrain ont permis d'étudier le type d'habitat et ses lieux d'entrée et de sortie des moustiques. Les populations ont des perceptions différentes des désagréments du paludisme ($p <0, 05$). Pour la prise en charge à domicile, les pratiques adoptées sont le recours à la médecine traditionnelle (55%), la médecine moderne (4,37%) et la prière (1,2%) ($p> 0, 05$). Par ailleurs, le niveau d'instruction influe sur les pratiques de lutte contre le vecteur. L'acceptabilité de la PID est de 98,7%.5 164 habitations humaines sont recensées dont 54,35% sont en ciment et 30,51% en terre de barre. Ceux construits en bambou et en bois représentent respectivement 11,1% et 3,7% ($p <0, 05$). 71,5% des murs sont lisses et se prêtent bien au traitement insecticide. En conclusion le recours aux mesures de prévention contre le paludisme et la connaissance de la PID ont été influencés par le niveau d'instruction. L'acceptation de la PID est totale. Une frange importante des habitations offrent les conditions d'une PID. Toutefois, nous suggérons la distribution de moustiquaires imprégnées d'insecticides à longue durée d'action pour les populations dont les maisons ne remplissent pas les conditions d'une PID.

SIII_P17. Comportement des anophèles en présence des moustiquaires imprégnées de deltaméthrine et/ou des supports imprégnés de bendiocarb.

R.Osse, F. Chandre, M. Akogbéto, J. Chabi, A. Djènontin & V. Corbel.

Mots clés : Résistance; Anophèles; supports imprégnés; Bendiocarb

Adresse : ossraz@yahoo.fr

De par l'importance médicale et socioéconomique du paludisme en Afrique et dans le contexte actuel de résistance des vecteurs aux insecticides, il est important de connaître le comportement des culicidés en présence des insecticides et surtout de rechercher de nouveaux outils de lutte anti vectorielle en zone de résistance des vecteurs aux insecticides. Le but de cette étude est d'analyser l'impact de trois interventions de lutte sur le comportement des anophèles à savoir : la couverture universelle en moustiquaires imprégnées de deltaméthrine à longue durée d'action (MILD), la couverture universelle en MILD associée à des bâches murales imprégnées de bendiocarb (MILD+Bâches) et la couverture sélective en MILD associée à la pulvérisation intra domiciliaire de bendiocarb (PID). Les moustiques ont été récoltés à l'aide de pièges aux fenêtres et par capture de faune résiduelle matinale après pulvérisation d'insecticide dans les villages traités par les différents outils de lutte anti vectorielle. Il ressort de l'analyse des résultats que l'association couverture sélective en MILD+ PID n'a pas d'effet dissuasif sur les vecteurs, par contre il augmente l'exophilie des culicidés (86,21%) qui entrent dans les cases. Par ailleurs, le taux de gorgement des femelles relevant les maisons traitées A + de 58,62% et la mortalité ?rale de 80,65%. L'association Bâches+ MILD a diminué le contact homme/vecteur. En effet, le taux de gorgement des femelles, l'exophilie et la mortalité sont respectivement de 51,58%, 93,16% et 80,67% dans ce bras. Pour ce qui est des traitements MILD, l'exophilie des culicidés est de 91,21% contre 66,51% dans le control. En ce qui concerne la mortalité elle est de 63,51% au niveau du traitement MILD et de 51,8% dans le control. Le taux de gorgement des femelles obtenu dans les traitements MILD (46,15%) a ? plus faible que celui obtenu dans le control (71,16%).

SIII_P18. Impact de l'environnement sur la sélection de la résistance d'*Anopheles gambiae* aux insecticides.

R. Aikpon, A. Yadouléton & M. Akogbéto.

Mots clés : *Anophèles gambiae*; Environnement; Résistance; Agriculture

Adresse : rockypremier@yahoo.fr

La résistance des vecteurs aux insecticides est un handicap à l'utilisation des matériaux imprégnés. Si l'étude sur la résistance des vecteurs revient à l'ordre du jour en Afrique, c'est dû à l'existence de plusieurs facteurs environnementaux qui induisent la résistance aux populations de moustiques, en occurrence *Anophèles gambiae*, principal vecteur du paludisme en Afrique de l'Ouest. C'est donc pour vérifier l'hypothèse d'une relation entre l'environnement et l'émergence de la résistance, que nous avons étudié la sensibilité de *Anophèles gambiae* aux insecticides. Des tests de sensibilité ont été réalisés avec des papiers imprégnés aux doses diagnostiques perméthrine à 0,75%, deltaméthrine 0,05 %, et du DDT 4% et du bendicarb à 1% dans les milieux écologiques variés : zone urbaine, zone maraîchère, zone rizicole zone cotonnière. Nous avons procédé aussi à une évaluation indirecte à partir de simulation de gîtes dont le but est de vérifier la présence de facteurs négatifs qui pourraient freiner le développement normal des larves de moustiques dans les gîtes larvaires. Par ailleurs, les paramètres physico-chimiques de l'eau des gîtes de développement de *An.gambiae* testés ont été mesurés pour vérifier une éventuelle relation entre ces paramètres et l'apparition de la résistance. Les résultats des ces travaux de recherche ont montré:

i) Sur la base de la réduction des taux de mortalité, *An. gambiae* apparaît résistant aux pyréthrinoïdes et au DDT dans les zones maraîchères urbaines et dans les sites cotonniers à traitement LEC et à traitement calendaire.
ii) Les Anophèles issus des zones cotonnières à traitement biologique sont sensibles aux insecticides agricoles
iii) Les traitements insecticides agricoles ont un impact négatif sur l'éclosion des œufs et la fréquence d'apparition de nymphes
iv) Il existe une relation entre les paramètres physicochimiques de l'eau des gîtes larvaires et la résistance, notamment la conductivité qui connaît une élévation par les traitements insecticides agricoles.
Au terme de ce travail, on peut conclure que les pratiques agricoles contribuent à la modification des paramètres physicochimiques des gîtes larvaires mais aussi à l'émergence de la résistance de population de *Anophèles gambiae*, principal vecteur du paludisme.

SIII_P19. Reduced efficacy of insecticide treated nets against pyrethroid resistant *Anopheles gambiae* in rural households, Southern Benin.

A.Asidi, R. N'Guessan, M. Akogbeto, C. Curtis & M. Rowland.

Key words: *Anopheles gambiae*; pyrethroids; resistance; households; Southern Benin
Address: alex.asidi@yahoo.com

The threat of pyrethroid resistance in West Africa is increasingly becoming a serious concern for the future usefulness of ITNs for malaria prevention. A field trial was carried out at household level in rural Benin to investigate the potential of torn ITNs to provide personal protection to sleepers under them. Four sites were selected with one in the North (Malanville) where *An. gambiae* is susceptible to insecticides and three in the South having *An. gambiae* carrying the knockdown resistance (kdr) gene. Untreated torn nets showed no protection in both the northern and the southern sites. However, at Malanville site after treatment with lambdacyhalothrin 18mg/m2, there was a significant blood feeding inhibition relative to control (65.3%) (P<0.001). By contrast blood feeding by *An. gambiae* was uninhibited at all three sites in the southern areas (P>0.05). Induced mortality of *An. gambiae* collected in households with treated nets from exit window traps fixed to sentinel houses was significantly higher in Malanville (70.1%) compared to that observed in one of the southern village, Fifadji, (10.9%) (p<0.001). Pyrethroid resistance in *An. gambiae* M molecular form in Southern Benin critically threatens to undermine the efficacy of ITNs. The personal protection of ITNs was lost under realistic field conditions and there is certainly no hope that the little amount of mosquitoes killed in the South will impact transmission during large scale control programmer.

SIII_P20. Impact of local washing practices on the efficacy of long lasting insecticide treated nets.

L.T.Pare, K. R. Dabire, A. Iabate, F. Y. Ouattara , M. J. C. Diannio, M. Akogbeto , T. Baldet, & P. Toe

Key words: Long lasting insecticide nets; washing practices; efficacy
Address: lea_toe@yahoo.com

The use of insecticide treated nets has been shown to be an effective preventive tool against malaria. But the re-treatment of net had a long time impeded the efficacy of net. To solve the difficulties related to bed net re-treatment, the long lasting insecticide net had developed to
replace the conventional insecticide treated net. But the concept of long lasting applied to the net, used and washed in field conditions had not sufficiently studied. The level of net efficacy related the duration of net using, and the local practices of washing (i.e. soaps used, frequencies of washing, soaking

process, washing process, rinsing process and drying process) was not known. This study based on socio-anthropological and entomological approaches analyzed long lasting concept by confronting long lasting insecticide nets with the local washing practices. During 36 months, qualitative and quantitative surveys had been conducted to describe community net washing practices. Bioassays and HPLC were realized to evaluate the efficacy of net after using by community and to measure the quantity of insecticide remaining in the net fibres. The main results of the study show that community soak their net with detergent before washing, used the modern soap, washed with hand, rinsed once, dried under sun and shade, washed on average twice per month. Efficacy of net varied according the washing practices and the formulation of insecticide on the nets. One year after net using the efficacy was stable, 15 months after using the efficacy was reduced following the washing practices being also efficacy until 24 months but after 36 months the efficacy decreased notably.

SIII_P21. The distribution of insensitive acetyl cholinesterase (ace-1R) in *Anopheles gambiae* s.l. populations from Burkina Faso (West Africa).

R.K. Dabiré, B. Yaro, M. Namountougou, L. Djogbenou, P. Kengne, F. Simard, T. Baldet, T. Martin & A. Diabaté

Key words: *Anopheles gambiae* s.s; ace.R mutation; organophosphates; carbonates; Burkina Faso
Address: dabire_roch@hotmail.com

Carbonates (CX) and organophosphates (OP) are considered as alternative to pyrethroids (PY) since many species of vectors including *Anopheles gambiae* had developed resistance to the latest ones especially for indoor spraying. But it is a prerequisite to test the susceptibility status of main malaria vectors to these insecticides before their use on large scale. This study is designed to evaluate the resistance status of *An. gambiae* s.l. to bendiocarb 0.1% (but also other insecticides including PY and OP) that is selected by the National Malaria Control Programme of Burkina Faso to be used in IRS in the West region of the country as an alternative to pyrethroid resistance reported in *An. gambiae.* We investigated through transversal sampling at 20 localities across the three different agro-climatic zones of Burkina Faso, the distribution of the acetyl cholinesterase insensitive mutation ace-1R, conferring the resistance phenotype to OP and CM in *An. gambiae* s.l. Specimens were identified by PCR assays and characterized for the ace.1R mutation. The overall collection of adults by indoor aerosol spray was a mix of *An. gambiae* s.s. and *An. arabiensis* across the Sudan (98.3% vs. 1.7%), Sudan-sahelian (78.6% vs. 21.4%) and the Sahel (91.5% vs. 8.5%) ecotypes. The S-form predominated in the Sudan sites from the West (69% vs. 31% for the M form) being very rare in the Sahel (100% of M form). The ace.1R mutation was dispersed throughout the Sudan and Sudan-sahelian localities at low frequencies (<50%) and was absent in the Sahel. It was overall most spread in the S form (0.32 vs. 0.036) but the highest frequency value was recorded in the M form (0.66). Few *An. arabiensis* were detected carrying this mutation in Bobo-Dioulasso town. However this mutation occurred in the two major climatic zones, its distribution overlapped mainly the cotton growing areas dispersed throughout the two zones. In conclusion the role of agricultural uses of insecticides facilitating the spread of the ace.1R mutation is discussed. These results are important at epidemiological level as OP and CM had been proposed to be used alone or in combination with pyrethroids as alternative strategy in vector control programs.

SIII_P22. Evidence of metabolic resistance to carbosulfan in addition to kdr-based mechanism resistance to pyrethroids within *Anopheles gambiae* in urban agriculture area in Benin.

M.B M Cissé, A. Zida, T. Baldet, F. Chandre, J.Chabi, J-M. Hougard, M. Akogbéto, O. A Koita & T.Martin.

Key words: *Anopheles gambiae*; vector control; metabolic resistance; Benin
Address: moussabina@yahoo.fr

Insecticide resistance of the main malaria vector, *Anopheles gambiae* threatens the efficacy of major insecticidal control tools such as Insecticide-Treated Nets and Indoor Residual Spraying. The objectives of this study were to assess the status of insecticide resistance in field populations of *An. gambiae*; then identified the resistance mechanisms implicated either target modification or metabolic detoxification. Anopheles larvae were sampled in Cotonou urban areas at the end of the dry and the beginning of the rainy season. The insecticide resistance status, the synergists PBO and TCPPE tests, and the selection with carbosulfan were carried out by using the WHO diagnostic bioassays kits. Species and molecular forms of *An. gambiae*, ace-1R and Leu-Phe kdr mutations were determined using PCR. Biochemical assays were conducted to confirm ace-1R mutation in individual mosquitoes and to detect any increase in the activity of enzymes involved in insecticide metabolism. All Anopheles sampled belonged to the M form of *An. gambiae* except one specimen of *An. arabiensis*. A strong resistance to DDT, carbosulfan, permethrin, and at a lower level to deltamethrin was detected in local population of *An. gambiae*. The cross resistance to DDT and pyrethroid was consistent with the high allelic frequency of the kdr mutation (87% in average). A significant increase in the amount of oxidases was diagnosed in local population compared to the susceptible reference strain. The level of resistance to carbamates decreased significantly when adult mosquitoes were preliminary exposed to PBO. The mean quantity of oxidase was significantly higher in the local population selected to carbosulfan. Biochemical tests detected a very low allelic frequency of the ace-1R mutation (6%). The scarcity of the ace-1R mutation was confirmed by the molecular diagnosis. This study underlines the multiple resistances to insecticides in the M form of *An gambiae*. The cross resistance to DDT and pyrethroids is mainly due to the Leu-Phe kdr mutation. The specific resistance to carbosulfan without implication of the ace-1R mutation is linked with overproduction of oxidases. Nevertheless, the detection of multiple resistance mechanisms in *An. gambiae* in Benin may represent a threat for the efficacy of vector control in next future.

SIII_P23. Le Master International d'Entomologie médicale et vétérinaire.

T. Baldet

Adresse : Thierry. Baldet@ird.fr

Comites du Symposium / Committees

President du Symposium

- Pr M. Akogbéto, Directeur CREC, Bénin, akogbetom@yahoo.fr

Comité scientifique / scientific committee

- Pr M. Akogbéto, Directeur CREC, Bénin, akogbetom@yahoo.fr
- Dr T. Baldet, IRD UR016 – CREC, Coordinateur MIE, Benin, thierry.baldet@gmail.com
- Dr B. Bucheton, IRD UMR177-CIRDES, Burkina Faso, bruno.bucheton@ird.fr
- Dr. M. Camara, coordonnateur PNLT, Guinée, mamadycamarafr@yahoo.fr
- Dr. V. Corbel, Responsable UR016 IRD-CREC, vincent.corbel@ird.fr
- Dr L. Djogbenou, IRSP, Bénin, luc.djogbenou@ird.fr
- Pr B. Fayomi Benjamin, Directeur de l'ISBA, Benin, bfayomi2@yahoo.fr
- Dr. A. Garcia, IRD UMR216, France, Andre.Garcia@ird.fr
- Pr O. Gaye, Université de Dakar, ogaye@refer.sn
- Dr M.-C. Henry, Coordonnatrice projet REFS, MAEE-CREC, Marie-Claire.Henry@ird.fr
- Dr Y. Imourou-Karimou, Coordonnateur PNLP, Bénin, imorou_yac@yahoo.fr
- Dr S.R. Kambiré, Coordonnateur PNLT, Burkina Faso, sierogerkambire@yahoo.fr
- Pr. D. Kindé Gazard, FSS/UAC, Bénin, kindegazard@yahoo.fr
- Dr J.P. Moulia-Pelat, Conseiller Régional Santé, SCAC-MAEE, Bénin, Jean-paul.MOULIA-PELAT@diplomatie.gouv.fr
- Dr I. Sidibé, Directeur Scientifique CIRDES, Burkina Faso, sambo@fasonet.bf
- Dr P. Simarro, Responsable du contrôle de la THA, OMS Genève, Suisse, SimarroP@who.int

Comite d'Organisation du Symposium (COS) / Organization Committee

- Mme A. Aicheou, secrétaire
- Mr L. Atigli, Assistant administratif CREC, constantlin@yahoo.fr
- Dr. V. Corbel, Responsable UR016 IRD-CREC, vincent.corbel@ird.fr
- Dr M.-C. Henry, Coordonnatrice projet REFS, MAEE-CREC, Marie-Claire.Henry@ird.fr

Partenariat & Sponsors / Partnership

Le Comité d'Organisation du Symposium remercie le partenariat institutionnel et les sponsors pour leur soutien.

The Organization Committee thanks the institutional partnership and the sponsors for their support.

Partenariat institutionnel / Institutional Partnership

CIRDES : Centre International de Recherche Développement sur l'Elevage en zone Subhumide
01 BP 454 Bobo-Dioulasso 01, Burkina Faso
Tél: (00226) 20 97 20 53 / 20 97 26 38; Fax : (00226) 20 97 23 20
Email: directiongenerale@cirdes.org;
www.cirdes.org

CREC : Centre de Recherche Entomologique de Cotonou
Ministère de la Santé, 06 BP 2604 Akpakpa, Bénin
Tél: (00229) 21 33 08 25
Email: akogbeto@leland.bj

FSS : Faculté des Sciences de la Santé de l'Université d'Abomey-Calavi (UAC)
01 BP 188 Cotonou, Bénin
Tél: (00229) 21 30 25 13; Fax: (00229) 21 30 40 96
Email: fsscotonou@yahoo.fr;
www.fss.bj.refer.org

IRD – Bénin: Institut de Recherche pour le Développement
01 BP 4414 Cotonou, Bénin
Tél: (00229) 21 30 03 54

Email: benin@ird.fr;
www.ird.fr/benin

ISBA: Institut des Sciences Biomédicales Appliquées
01 BP : 918 Cotonou, Bénin
Tél: (00229) 21 30 55 65
Email: **isba@internet.bj**

IRSP: Institut Régional de Santé Publique
Ouidah, Route des Esclaves 01 BP 918 Cotonou, Bénin
Tel: (00229) 21 34 16 74 / (00229) 21 34 16 75; Fax: (00229) 21 34 16 72
Email: irspadm@internet.bj;
www.irsp.bj.refer.org

Liberté • Égalité • Fraternité
RÉPUBLIQUE FRANÇAISE

Ministère des Affaires Etrangères et Européennes
Direction Générale de la Mondialisation, du Développement et des Partenariats.
27, rue de la Convention
CS 91533-75732 Paris CEDEX 15
www.diplomatie.gouv.fr

PNLP Bénin: Programme National de Lutte contre le Paludisme
Ministère de la Santé, AKPAKPA 01 BP 882 Cotonou, Bénin
Tél. / Fax: (00229) 21 33 39 30
Email : contact@pnlp.info;
www.pnlp.info

PNLTHA Burkina Faso: Programme National de Lutte contre la Trypanosomose Humaine Africaine
Ministère de la Santé; BP: 7009, Ouagadougou, Burkina Faso
Tél: (00226) 70 26 97 63 /76 52 61 87, Fax: 50 31 54 40
Email: sierogerkambire@yahoo.fr

PNLTHA
Guinée

PNLTHA Guinée: Programme National de Lutte contre la Trypanosomose Humaine Africaine
Ministère de la Santé, BP 817 Conakry, Guinée
Tel: (00224) 60 59 89 46 / (00224) 64 83 71 99
Email: mamadycamarafr@yahoo.fr

Sponsors

Service de Coopération et d'Action Culturelle,
Ambassade de France à Cotonou
Avenue Jean-Paul II - BP 966 – Cotonou, Bénin
Tél: (00229) 21.30.02.25 / 21.30.02.26
Email : scac.cotonou@diplomatie.gouv.fr

Bayer CropScience AG
Alfred Nobel Str. 50, D - 40789 Monheim am Rhein, Germany
www.bayercropscience.com

Sanofi-aventis France
9, bld Romain Rolland, 75159 Paris Cedex 14
Tél: (0033) 1 57 63 33 33
www.sanofi-aventis.fr

IRD – Bénin: : Institut de recherche pour le développement Le Sextant
44, bd de Dunkerque, CS 90009, 13572 Marseille cedex 02
Tél. : (0033) 4 91 99 92 00 ; Fax (0033)4 91 99 92 22
www.ird.fr

Projet FSP REFS : Recherche en Entomologie, Formation et Stratégies de Prévention : le cas du paludisme et de la trypanosomiase humaine africaine
CREC, 06 BP 2604, Cotonou, Bénin.
Tél : (00229) 21 08 26 13 / 97 39 58 17
Email : Marie-Claire.Henry@ird.fr

Vestergaard Frandsen New York
100 Park Avenue, 16th Floor, New York, NY 10017, USA
Tel: (001) 212 984-1026
Email:media@vestergaard-frandsen.com;
www.vestergaard-frandsen.com

SUMITOMO CHEMICAL
27-1, Shinkawa 2-chome, Chuo-ku, Tokyo 104-8260, Japan
Tel: (81) 3-5543-5798
Fax: (81) 3-5543-5947
Email:tanakah10@sc.sumitomo-chem.co.jp;
www.olyset.net

Le Réseau ABC
01 BP 4414 RP Cotonou
Tel: (00229) 21 05 41 41
Email:vincent.corbel@ird.fr
www.mpl.ird.fr

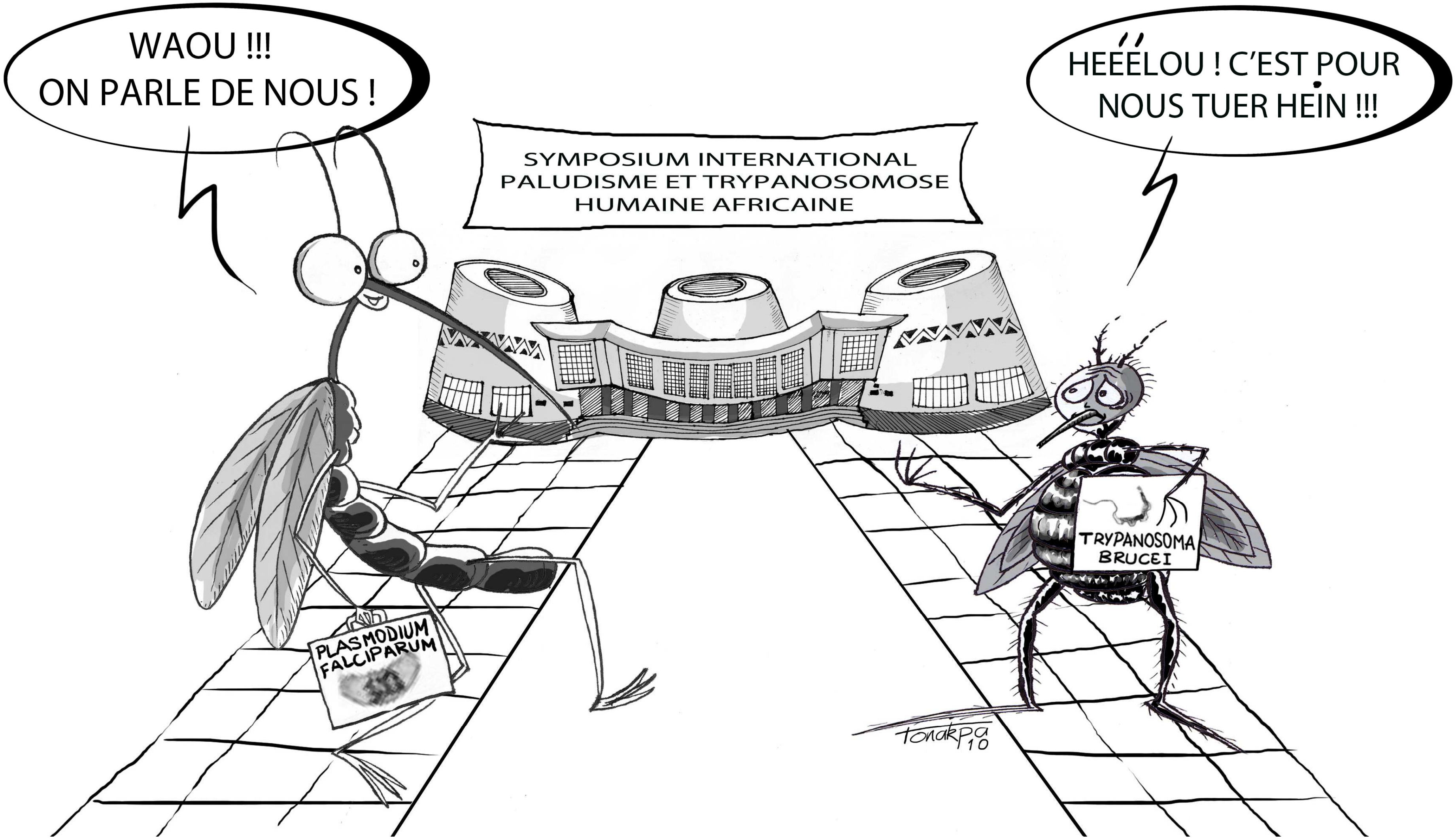
WAOU !!!
ON PARLE DE NOUS !
SYMPOSIUM INTERNATIONAL
PALUDISME ET TRYPANOSOMOSE
HUMAINE AFRICAINE
HEÉÉLOU ! C'EST PỌUR
NOUS TUER HEIN !!!
PLASMODIUM
FALCIPARUM
TRYPANOSOMA
BRUCEI
Tonakpa 10
