# Supplementary material for: Prevention and control of malaria and sleeping sickness in Africa: Where are we and where are we going?
Source: Parasit Vectors. 2011 Mar 16;4:37. doi: 10.1186/1756-3305-4-37 (PMC3065431; doi:10.1186/1756-3305-4-37)
Supplement: Additional file 2 — Scientific Committee. [file 1756-3305-4-37-S2.DOC]

## Additional File 2

## Scientific Committee

**Benin**: Pr M. Akogbeto, Director of CREC, UAC University; Dr T. Baldet, IRD UR016–CREC, MIE coordinator; Dr V. Corbel, UR016 IRD-CREC; Dr L. Djogbenou, IRSP; Pr B. Fayomi, Director of ISBA; Dr M.-C. Henry, coordinator REFS, MAEE-CREC; Dr Y. Imourou-Karimou, coordinator NMCP; Pr D. Kindé Gazard, FSS-UAC; Dr J.P. Moulia-Pelat, Regional Health Adviser, SCAC-MAEE.

**Burkina Faso**: Dr B. Bucheton, IRD UMR177-CIRDES; Dr S.R. Kambiré, coordinator NSSCP.

**France**: Dr A. Garcia, IRD UMR216.

**Guinea**: Dr M. Camara, coordinator NSSCP.

**Senegal:** Pr O. Gaye, University of Dakar.

**Suisse**: Dr P. Simarro, Human African Trypanosomiasis, HTM/NTD/IDM, WHO HQ
